# Transcriptome profile of *Corynebacterium pseudotuberculosis* in response to iron limitation

**DOI:** 10.1186/s12864-019-6018-1

**Published:** 2019-08-20

**Authors:** Izabela Coimbra Ibraim, Mariana Teixeira Dornelles Parise, Doglas Parise, Michelle Zibetti Tadra Sfeir, Thiago Luiz de Paula Castro, Alice Rebecca Wattam, Preetam Ghosh, Debmalya Barh, Emannuel Maltempi Souza, Aristóteles Góes-Neto, Anne Cybelle Pinto Gomide, Vasco Azevedo

**Affiliations:** 1 Laboratório de Genética Molecular e Celular, Departamento de Biologia Geral, Instituto de Ciências Biológicas, Universidade Federal de Minas Gerais, Belo Horizonte, MG Brazil; 2 Departamento de Bioquímica e Biologia Molecular, Instituto de Ciências Biológicas, Universidade Federal do Paraná, Curitiba, PR Brazil; 3 Departamento de Biointeração, Instituto de Ciências da Saude, Universidade Federal da Bahia, Salvador, BA Brazil; 4 Biocomplexity Institute and Initiative, University of Virginia, Charlottesville, VA USA; 5 Department of Computer Science, Biological Networks Lab, Virginia Commonwealth University, Richmond, VA USA; 6 Department of Microbiology, Institute of Biological Sciences, Federal University of Minas Gerais (UFMG), Belo Horizonte, MG 31270-901 Brazil

**Keywords:** *Corynebacterium pseudotuberculosis*, Iron homeostasis, Iron-regulated transcriptional factors, Heme acquisition, Differential gene expression

## Abstract

**Background:**

Iron is an essential micronutrient for the growth and development of virtually all living organisms, playing a pivotal role in the proliferative capability of many bacterial pathogens. The impact that the bioavailability of iron has on the transcriptional response of bacterial species in the CMNR group has been widely reported for some members of the group, but it hasn’t yet been as deeply explored in *Corynebacterium pseudotuberculosis*. Here we describe for the first time a comprehensive RNA-seq whole transcriptome analysis of the T1 wild-type and the Cp13 mutant strains of *C. pseudotuberculosis* under iron restriction. The Cp13 mutant strain was generated by transposition mutagenesis of the *ciuA* gene, which encodes a surface siderophore-binding protein involved in the acquisition of iron. Iron-regulated acquisition systems are crucial for the pathogenesis of bacteria and are relevant targets to the design of new effective therapeutic approaches.

**Results:**

Transcriptome analyses showed differential expression in 77 genes within the wild-type parental T1 strain and 59 genes in Cp13 mutant under iron restriction. Twenty-five of these genes had similar expression patterns in both strains, including up-regulated genes homologous to the hemin uptake *hmu* locus and two distinct operons encoding proteins structurally like hemin and Hb-binding surface proteins of *C. diphtheriae*, which were remarkably expressed at higher levels in the Cp13 mutant than in the T1 wild-type strain. These hemin transport protein genes were found to be located within genomic islands associated with known virulent factors. Down-regulated genes encoding iron and heme-containing components of the respiratory chain (including *ctaCEF* and *qcrCAB* genes) and up-regulated known iron/DtxR-regulated transcription factors, namely *ripA* and *hrrA*, were also identified differentially expressed in both strains under iron restriction.

**Conclusion:**

Based on our results, it can be deduced that the transcriptional response of *C. pseudotuberculosis* under iron restriction involves the control of intracellular utilization of iron and the up-regulation of hemin acquisition systems. These findings provide a comprehensive analysis of the transcriptional response of *C. pseudotuberculosis,* adding important understanding of the gene regulatory adaptation of this pathogen and revealing target genes that can aid the development of effective therapeutic strategies against this important pathogen.

**Electronic supplementary material:**

The online version of this article (10.1186/s12864-019-6018-1) contains supplementary material, which is available to authorized users.

## Background

Caseous lymphadenitis (CLA) is a chronic, debilitating infection caused by the Gram-positive bacterium *Corynebacterium pseudotuberculosis*. CLA affects mainly small ruminants, such as sheep and goats, and is the leading cause of economic loss associated with the reduction of wool, meat and milk production, and also results in carcass and skin condemnation in the majority of sheep and goat production areas [1]. CLA infection is typically initiated by inoculation via oral, respiratory, and membrane wounds [2]. Once inside, the bacteria are phagocytosed by macrophages, which are then drained to local lymph nodes. Once internalized, the bacteria evade the cellular immune response mechanisms, being able to survive and rapidly multiply within the macrophage phagosome. Bacterium proliferation results in the death of the host cell and the subsequent release of bacteria into the extracellular environment, which can then spread through the lymphatic system to regional lymph nodes and internal organs [3]. Clinical signs of the disease include the formation of caseous abscesses either in superficial or internal lymph nodes. While antibiotic therapy is not usually helpful, management of the disease combines the identification and removal of infected animals along with vaccination of healthy animals [2].

In recent years, novel mechanisms have been identified as potential targets to combat infections caused by corynebacteria. Considerable progress has been made in understanding mechanisms involved in iron acquisition and homeostasis in pathogenic species that can potentially be used in the development of strategies to control the pathogen [4]. Iron is fundamental in a variety of cellular functions and its capacity to directly participate in redox reactions, donating or accepting electrons, highlights its central importance in biological processes including DNA synthesis, enzyme and redox catalysis, electron transport, respiration, cellular survival and growth [5, 6]. Given its absolute requirement by all bacteria, iron acquisition is a fundamental step for the establishment of the infection by pathogenic species, and iron withholding is one of the first host defense mechanism used to prevent bacterial growth [7]. Under physiological conditions, free ferric iron is insoluble and therefore is poorly accessible to support microbial growth. Within the host, iron is kept within intracellular proteins such as hemoglobin, cytochrome, ferritin or is found chelated by transferrin or lactoferrin in the extracellular compartment, in an attempt to further decrease iron accessibility to pathogens [8, 9]. To overcome the low bioavailability of the metal imposed by the host, pathogenic bacteria coordinate a complex and sensitive iron-dependent transcriptional regulatory network that controls the expression of genes related to iron homeostasis, including uptake, storage and iron-dependent systems according to the availability of the metal [10]. In pathogenic bacteria, the uptake is the most tightly regulated process in iron homeostasis, which is highlighted by the diverse complement of mechanisms used to efficiently exploit the host iron sources [10, 11].

In a previous report, we described the construction of a *ciuA* mutant in *C. pseudotuberculosis,* designated Cp13 [12]. The Cp13 mutant was generated by using the in vivo insertional mutagenesis of the reporter transposon-based system TnFuZ in the strain T1 [12]. The molecular characterization of the Cp13 mutant showed that the insertion disrupted the *ciuA* gene, which encodes a putative-iron transport binding protein of the *ciuABCD* operon [12]. In *C. pseudotuberculosis*, the *ciuA* gene appears to be organized into the *ciuABCD* operon comprising a six gene-cluster, *ciuABCDEF,* which shares high protein similarity with components of siderophore ABC-type transport systems. The *ciuA* gene shares amino acid similarity to periplasmatic binding proteins from Gram-negative bacteria and 73% sequence identity to a putative iron transport binding protein from *C. diphtheriae* (DIP0582). In *C. diphtheriae*, the predicted product of the *ciuA* gene is a lipoprotein of 298 amino acids responsible for the transportation of iron-siderophore complexes. The *ciuB*, *ciuC* and *ciuD* genes encode proteins possibly related to siderophore ABC-type transporters. Downstream from the *ciuABCD* operon is the *ciuE* gene, whose predicted product shares homology to enzymes responsible for the biosynthesis of siderophore aerobactin [13, 14]. The *ciuF* gene has low similarity to integral membrane efflux proteins and its role in siderophore transportation is still unknown. In addition to the *ciuABCDEF* genes, the genome of *C. pseudotuberculosis* harbors another putative iron siderophore system, formed by the *fagABCD* genes. Similar to *ciu* clusters, the *fagABC* operon and the *fagD* gene encode proteins with similarity to siderophore ABC-type transporters identified in other pathogenic species. It has also been previously demonstrated that the cluster is regulated by iron, being associated with the pathogenesis of *C. pseudotuberculosis* [15]. While the relevance of the *ciu* cluster regarding iron acquisition remained unclear for *C. pseudotuberculosis*, experimental studies conducted by our group with the Cp13 mutant strain have demonstrated a link between the disruption of the *ciuA* gene and virulence of the mutant strain [12]. The Cp13 mutant presented a substantial reduction in virulence with a decrease in abscess formation in experimental CLA infections [12]. Moreover, in a previously reported vaccination trial conducted with the Cp13 mutant, our group has demonstrated that the Cp13 mutant was able to elicit both humoral and cellular responses in immunized mice, with significant production of IgG and IgG2a. In addition, 80% of the mice immunized with the Cp13 mutant strain survived after being challenged with a virulent *C. pseudotuberculosis* strain. This mutant strain also showed reduced intracellular viability in murine cells [16].

As previously stated, iron acquisition is crucial for the growth and development of many bacterial pathogens; however, the concentration that is freely accessible within the host is much lower than the concentration required for bacterial growth [10]. While the iron uptake process has already been well characterized in Gram-negative bacteria, our knowledge regarding iron acquisition in Gram-positive pathogens has only recently been examined. In the *C. pseudotuberculosis* genome, there are twenty putative described genes directly involved in the acquisition and metabolism of iron; of which many have been associated with the pathogenesis of the bacterium [15–17]. The virulence of *C. pseudotuberculosis*, as for many pathogenic species, depends on their ability to survive the harsh iron-limited environment inside the host [10]. However, despite the clear importance of iron during infection, our knowledge regarding *C. pseudotuberculosi*s sensing and regulating of genes involved in iron homeostasis is limited. Driven by the paucity of information on the acquisition of iron in *C. pseudotuberculosis,* our primary goal was to comprehensively characterize the transcriptional iron response of this important pathogen, as well as its association with growth and virulence under a condition similar to the iron-limited environment which is encountered by the bacterium during the infection inside the host. In addition, since siderophore-mediated iron acquisition seems to play a critical role in iron homeostasis for many bacteria pathogens [18], we hypothesized that the *ciuA* gene was also essential for the uptake of iron in *C. pseudotuberculosis*. To address this hypothesis, we compared gene expression and growth profiles between the mutant (Cp13) and wild-type (T1) strains in the presence of the iron chelator 2,2′-dipyridyl-DIP (Low Iron) and in the absence of the chelating agent (High Iron). To our knowledge, this is the first report addressing the complexity of the *C. pseudotuberculosis* response to iron limitation.

## Results

### The effects of iron chelating on the viability and growth of *C. pseudotuberculosis* strains

**Fig. 1:**
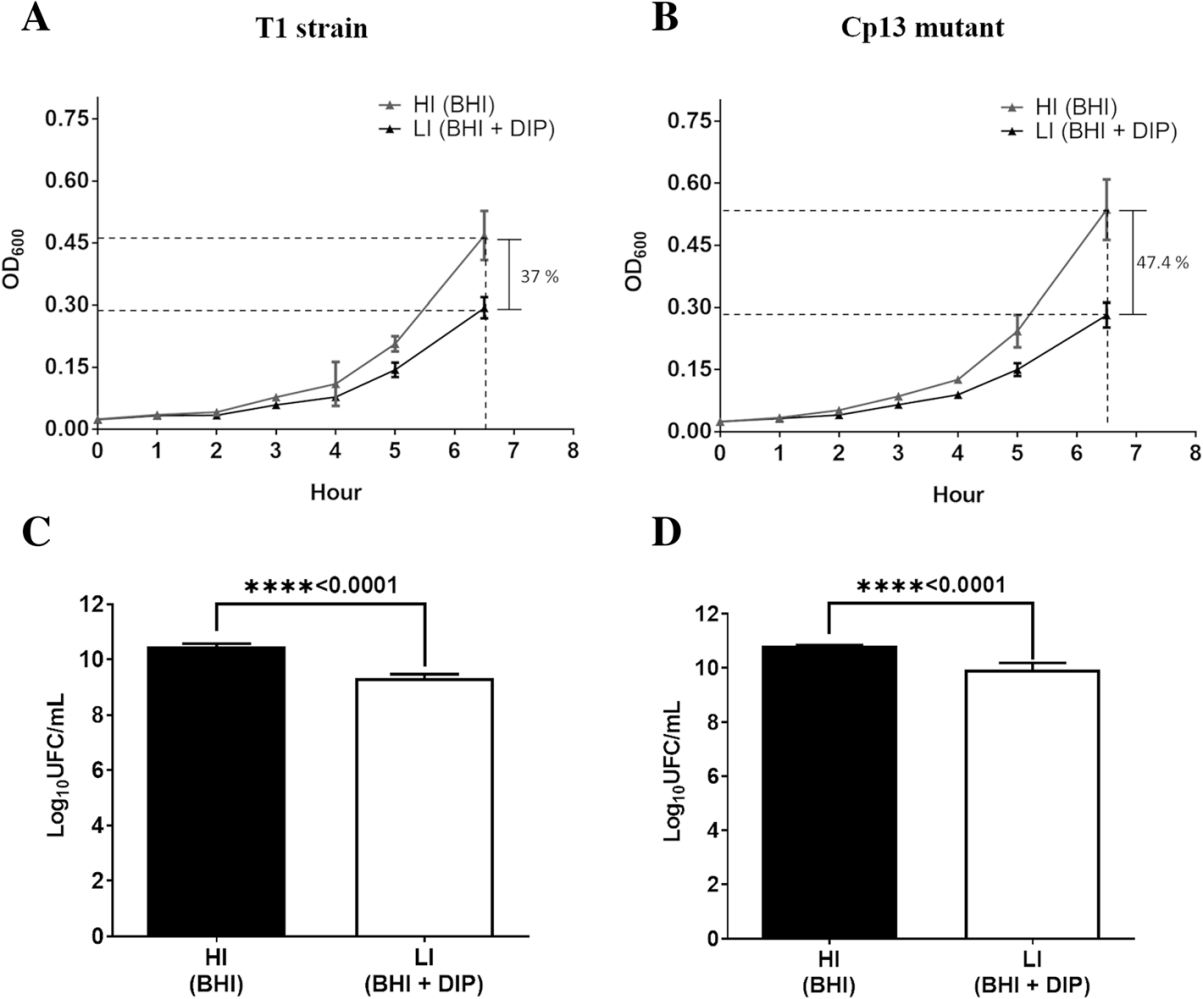
*C. pseudotuberculosis* growth assays. Growth proliferation was determined by measuring the OD of low iron (LI) and high iron (HI) cultures at 600 nm of the wild-type (parent) T1 strain and the Cp13 mutant strain, reflecting the growth dynamics of the bacterial strains in the culture media over the different time points. BHI broth was supplemented with 250 μM of the iron chelator 2,2′-dipyridyl (DIP) to iron stress the bacteria. The stress was confirmed at 6h30min with a significant reduction in the final culture density of both strains. At the 6h30min, both strains presented a final LI culture density of 63 and 52.6% of the bacteria grown in the high iron BHI broth **a** wild-type T1 strain and **b** Cp13 mutant. *C. pseudotuberculosis* viability was measured by determining the number of CFU/mL after 6h30min of incubation in a low iron (LI) dipyridyl-chelated and high iron (HI) non-chelated media. Average log10 (CFU/mL) along with the standard deviation (error bars) for the experimental cultured conditions (HI vs LI) from three individual biological replicates are represented. Differences between HI and LI for the wild-type T1 strain and Cp13 mutant were shown to be statistically significant using a two-tailed *t* test with a *p*-value < 0.001(****). Log10 (CFU/mL) in the **c** wild-type T1 strain and the **d** Cp13 mutant

To assess the effects of iron depletion on bacterial viability and growth we compared the number of CFU/mL and the rate of proliferation for each strain between a low (LI - dipyridyl-chelated) and high (HI - non-chelated) iron condition (Fig. 1). Both cultures showed similar growth characteristics, with no statistical differences observed in the growth rate of the high iron medium cultures (student *t*-test with *p* > 0.05), with the Cp13 mutant achieving cellular density similar to the wild-type T1 strain (Additional file 2: Figure S2). As expected, both bacterial strains were iron stressed in the iron depleted environment with a significant reduction in growth of the Cp13 mutant relative to the wild-type T1 strain (*p*-value = 0.0016) (Additional file 2: Figure S2). Moreover, inhibition of bacterial growth was evident at 6 h 30 min of incubation, resulting in a 47.4% reduction in cellular density of the Cp13 mutant grown in the LI condition compared to the HI condition (Fig. 1b). Similarly, we observed a 37% reduction in bacterial growth of the wild-type T1 strain following the addition of the iron chelator (Fig. 1a). In addition, an approximately 1-log reduction was observed in the CFU counts in the presence of the iron chelator when compared to the high iron media for both strains (Fig. 1c/d).

### Sequencing statistics and data quality assessment

In vitro cultures of the wild-type T1 strain and Cp13 mutant were grown in BHI medium supplemented with the iron-chelator dipyridyl (LI- low iron) as previously described and parallel cultures of BHI medium (HI- high iron) were used as reference (control). High-throughput RNA sequencing (RNA-seq) transcriptome profiling was used to characterize the differential gene expression (DGE) between LI and HI cultures of the wild-type T1 strain and Cp13 mutant. Two rounds of RNA sequencing were performed on the Ion Proton system yielding over 120 million single-end reads. Sequencing data were collected across 4 independent sets of paired biological samples (HI and LI) for the Cp13 mutant and 3 sets of paired samples for the wild-type T1 strain and labeled as batches A-C (See Additional file 1: Table S1 for batch identification). One HI sample from the T1 strain had a low total number of sequenced reads, with less than < 0.4% of the total sequences for its LI paired-sample, 38,405 and 12,560,458 respectively. As a result, both of these paired samples were removed from further analyses. Detailed information on the sequencing statistics of each sample is provided in Additional file 1: Table S1.

The remaining samples were quality checked and had an average read quality Phred score of 24–25 with a mean per base quality score of over 20. An average base quality trimming was performed, with less than 1% of the total reads from each set excluded (Additional file 1: Table S2). Trimmed reads were mapped to the 1002B reference genome. The percentage of reads mapping to the genome ranged from 72.6 to 98.9% (Additional file 1: Table S3). Unambiguously aligned reads were identified by HTSeq-count, which excluded all reads mapped to or overlapping with more than one gene in the reference (Additional file 1: Table S4).

Principal component analysis (PCA) was used to assess the relationship between samples for both strains (Additional file 1: Figure S1). PC1 and PC2 explain 85% of the total variance in the dataset. PC1 revealed a clear separation between experimental conditions (HI and LI). Except for one HI sample, batches B and C of the Cp13 mutant and T1 strain datasets grouped together, indicating low inter-strain variability among replicates of the same experimental batch. PC2 clearly evidenced the variability of the paired-sample 1 of the Cp13 mutant (batch A) batches B-C of the same strain. Batch effect was attributed to sample processing and *batch* effects were therefore controlled for in the subsequent differential expression analysis of the Cp13 mutant by adding batch as a variable into the experimental design in DESeq2 [19] (Additional file 1: Figure S1).

### DE analysis of iron-regulated genes

**Fig. 2:**
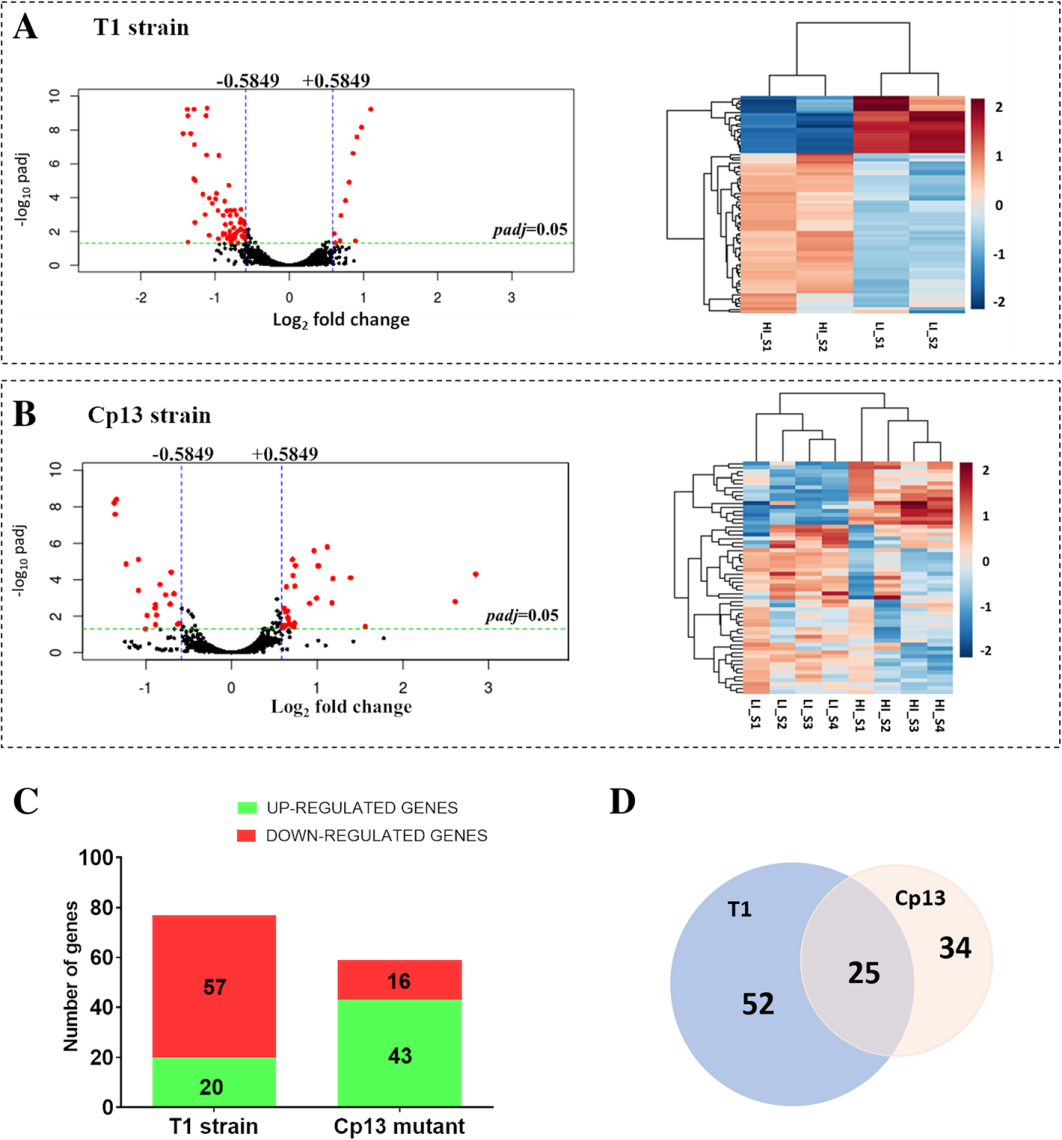
Differential gene expression of *C. pseudotuberculosis* CpT1 strain and Cp13 mutant under iron limitation. Counts were normalized using DESeq2 and differentially expressed genes were filtered using a false discovery rate (FDR) of < 0.05 and a log2fold change > 0.5849 or < − 0.5849 for biological significance. Volcano plots (on the left) of the log2-fold change of each detected gene in relation to their -log10 of adjusted *p* values and heatmaps (on the right) are shown in figure **a** for the 77 differentially expressed genes in the wild-type T1 strain and figure **b** shows 59 DEGs in the Cp13 mutant. In the volcano plots threshold of log2fold change and *p* adjusted value are represented by blue and green lines, respectively. *Rlog*-transformed normalized counts in the heatmap were clustered based on Euclidean distance. Rows indicate genes and columns represent individual samples from the two experimental conditions (LI and HI represent low iron and high iron conditions, respectively). **c** Stacked-bar graph representing the total number of the up and down-regulated genes identified in the T1 and Cp13 strains, considering a greater than 1.5-fold change (log2fold change 0.5849 and − 0.5849), where 57 and 16 genes with diminished expression, 20 and 43 genes with increased expression under iron restriction are specified. **d** Comparative analysis of the DEGs between wild-type and Cp13 mutant is represented by a scaled Venn diagram showing 52 genes expressed only in the wild-type T1 strain, 34 genes expressed only in the Cp13 mutant and 25 genes common to both wild-type and mutant (intersection)

To estimate the relative gene expression across sample groups, pairwise analyses were conducted in counts of uniquely mapped reads of LI against HI cultures for each strain with the DESeq2 R package. Adjusted *p*-values were obtained using the Benjamin and Hochberg false discovery rate (FDR) method, and an adjusted *p*-value of 0.05 and a log2fold change of − 0.5849/0.5849 (fold change of 1.5) were set as a cutoff for significant differential expression. A total of 77 (T1- Fig. 2a) and 59 (Cp13- Fig. 2b) genes were found to be differentially expressed between LI and HI conditions, of which, 25 genes were commonly expressed in the wild-type T1 strain and Cp13 mutant (Fig. 2d). In the wild-type T1 strain, 20 genes were identified up-regulated and 57 were down-regulated, while 43 genes were up-regulated, and 16 genes were down-regulated in the mutant strain (Fig. 2b). The distribution and expression profile for each strain was represented by heatmap and volcano plots of all significant differentially expressed genes with a log2fold change of 0.5849/− 0.5849 at a 5% FDR (Fig. 2a/b). Complete DE gene lists for each of the strain are provided in Additional file 3.

### Differential gene expression analysis under iron restriction in the wild-type T1 strain

**Fig. 3:**
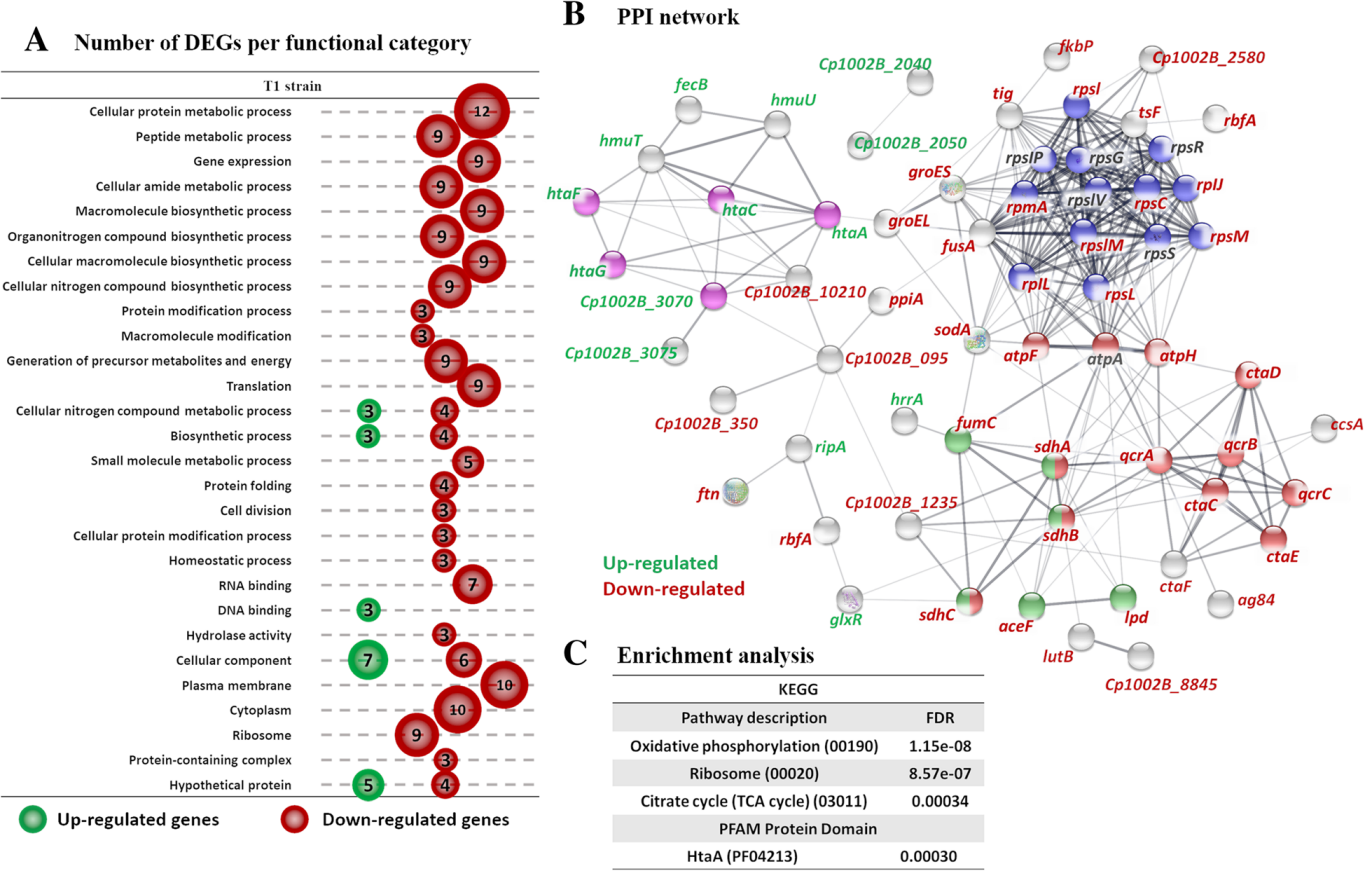
Functional annotation and PPI analysis of the DEGs in the wild-type T1 strain. **a** The number of differentially expressed genes are shown by functional categories, where circle sizes are proportional to the number of genes with significant differential expression. Green circles represent genes with increased expression and red circles represent genes with diminished expression. Only terms with > 2 genes assigned to a functional category are shown. **b** PPI analyses were carried out using the STRING database analysis tool and line thickness indicates the strength of data support for each interaction. Only connected nodes and interaction with a medium (> 0.4), high (> 0.7) and highest confidence (> 0.9) are visualized in the network. Node colors represent enriched functional categories and gene identification color represents up-regulation (green), down-regulation (red) and unchanged expression (gray). *p*-value of PPI interactions indicates significance of protein association. **a** The STRING PPI network of the T1 strain contained 75 nodes and 298 edges with PPI interaction enrichment *p*-value of < 1.0e-16 (Additional file 5: Figure S4). 4 enriched categories are shown: TCA cycle (green), oxidative phosphorylation (red), ribosome (blue) and HtaA domain (pink). **c** Enrichment analysis was conducted using STRING and significant expressed categories (FDR < 0.01) are indicated

To investigate the impact of iron restriction in the wild-type T1 strain of *C. pseudotuberculosis* a DEG analysis was performed comparing LI vs HI conditions between biological replicates for each condition. A total of 77 genes were found differentially expressed with increased expression of 20 genes and decreased expression of 57 genes in the wild-type T1 strain (Fig. 2a). Regarding gene ontology classification, most of the DEGs belonged to the categories of cellular metabolic processes (GO:0044267, GO:0006518, GO:0043603, GO:0009059, GO:1901566, GO:0034645, GO:0044271), generation of precursor metabolites and energy (GO:0006091), translation (GO:0006412), cellular nitrogen compound metabolic process (GO:0043167), biosynthetic process (GO:0009058), RNA binding (GO:0003723), hydrolase activity (GO:0016818), plasma membrane (GO:0005886), cytoplasm (GO:0005737), and ribosome (GO:0005840) (Fig. 3a). Interestingly, all genes assigned to these categories were down-regulated, suggesting that iron restriction prompted a decrease in expression of genes involved in energy metabolism, biosynthetic processes, and translation in the wild-type T1 strain. Specifically, 12 DEGs involved in energy metabolism, including TCA cycle (*sdhC*, *sdhB*, and *lpd*), ATP production (*atpF*, *atpH*), pyruvate metabolism (*lpd*), and oxidative phosphorylation (*qcrC, qcrA, qcrB, ctaC, ctaF, ctaE*, and *ctaD*). It also draws our attention to the down-regulation of genes involved in ribosome and translation categories (*rplJ, rplL, rplM, rpmA, rpsC, rpsI, rpsL, rpsM).* See Additional file 4: Table S7 for the full listing of GO terms identified. In the T1 strain, the *rplJ, rplL, rplM, rpmA, rpsC, rpsI, rpsL, rpsM* genes were down-regulated by over 1.5-fold difference together with the other down-regulated genes *fusA* and *tsf*, which encode the elongation factors EF-G and EF-Ts associated with translation. The categories cellular nitrogen compound metabolic process (GO:0043167), biosynthetic process (GO:0009058), DNA binding (GO:0003677), and cellular component (GO:0005575) were the only categories with significant and differential increased expression under iron restriction in the T1 strain (Fig. 3a). Specifically, the three genes with increased expression from the DNA binding, biosynthetic process, and cellular nitrogen compound metabolic process groups were *glxR*, *ripA*, and *hrrA*, which are involved in the transcriptional regulation of metabolic processes (*glxR*) and the expression of iron-dependent genes (*ripA* and *hrrA*) [20–23]. Among the genes assigned to term cellular component, we observed an increase in the expression of genes encoding components of the membrane, which play a role in the acquisition and transportation of hemin (*htaA*, *htaC*, *htaF*, *htaG*, *Cp_3070*, and *Cp_3075*). Protein-protein interaction (PPI) analysis was conducted to investigate the direct and functional association between the differentially expressed genes. PPI and enrichment analyses were conducted using the STRING database [24] and visualized using the Cytoscape software [25]. The PPI network constructed using the DE genes of the wild-type T1 strain consisted of 75 nodes and 298 edges with a confidence score of ≥0.4 (Fig. 3b/Additional file 5: Table S9). 23 genes with more than 10 interactions were selected as representative hub genes. The most significant node genes were *atpF, atpH, ctaC, ctaD, ctaE, qcrA, qcrB, qcrC, sdhA, sdhB, sdhC, rplJ, rplL, rplM, rpmA, rpsC, rpsI, rpsL, rpsM, grosS, tsF, fusA*, and *sodA*. These genes were all down-regulated and mainly associated with energy metabolism, ribosome biogenesis, and translation. Enrichment analysis confirmed the repression of the oxidative phosphorylation (KEGG 00190, *p* adjusted value = 1.15e-08), ribosome (KEGG 03010, *p* adjusted value = 8.57e-07) and the tricarboxylic acid cycle (TCA cycle – KEGG 00020, *p* adjusted value = 0.00034) pathways under iron restriction in the wild-type T1 strain (Fig. 3c). Additionally, genes encoding proteins with a HtaA hemin-binding domain (PFAM PF04210, *p* adjusted value = 0.00030) were also enriched among the DEGs in the wild-type T1 strain (Fig. 3c).

### Differential gene expression analysis under iron restriction in the Cp13 mutant

**Fig. 4:**
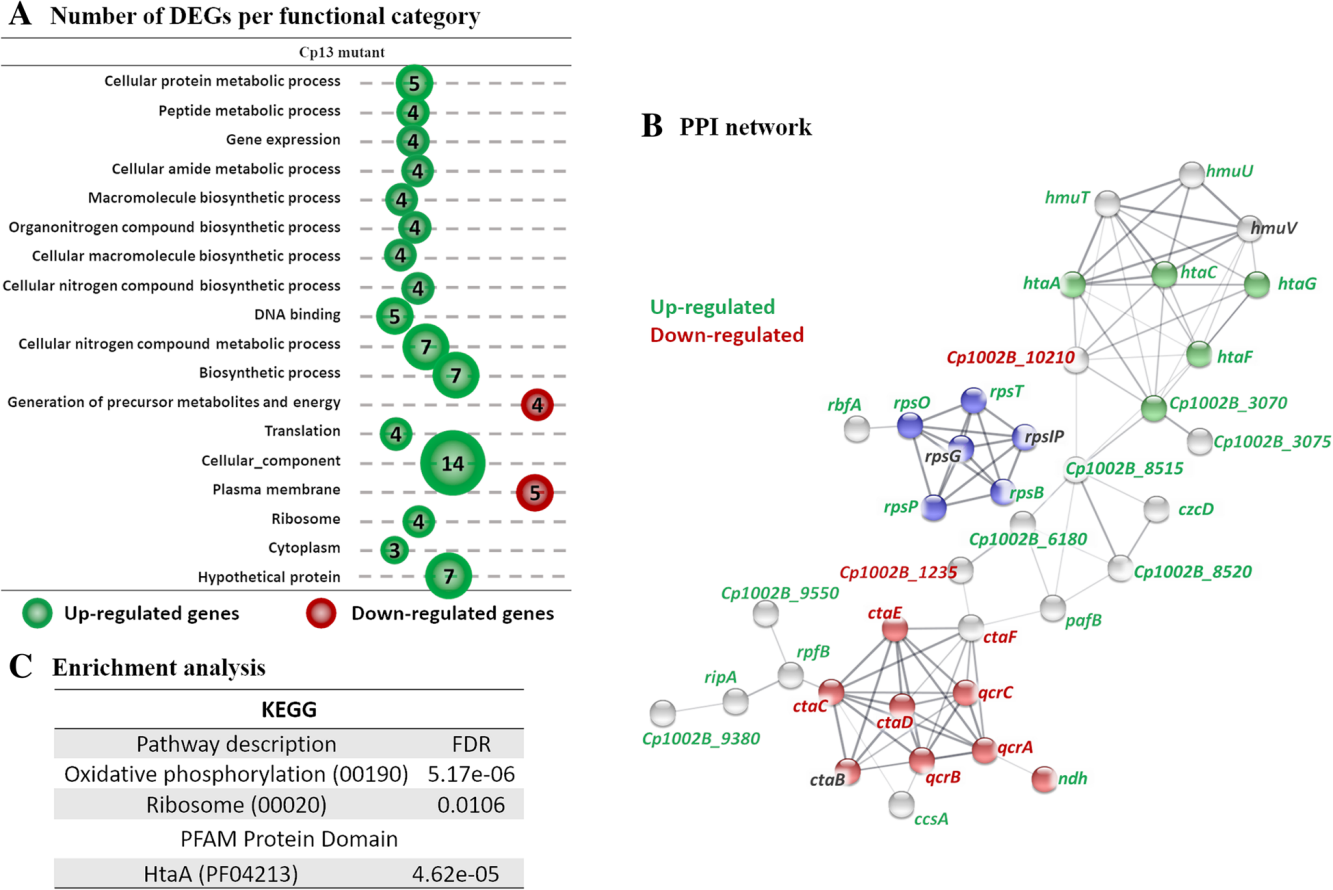
Functional annotation and PPI analysis of the DEGs in the Cp13 mutant. **a** The number of differentially expressed genes are shown by functional categories, where circle sizes are proportional to the number of genes with significant differential expression. Green circles represent genes with increased expression and red circles represent genes with diminished expression. Only terms with > 2 genes assigned to a functional category are shown. **b** PPI analysis was carried out using the STRING database analysis tool and line thickness indicates the strength of data support for each interaction. Only connected nodes and interaction with a medium (> 0.4), high (> 0.7) and highest confidence (> 0.9) are visualized in the network. Node colors represent enriched functional categories and gene identification color represents up-regulation (green), down-regulation (red) and unchanged expression (gray). *p*-value of PPI interactions indicates significance of protein association. Cp13 PPI interactions contained 56 nodes and 94 edges with a PPI interaction enrichment *p*-value of 6.02e-14 (Additional file 5: Figure S4). Three enrichments are shown: oxidative phosphorylation (red), ribosome (blue) and HtaA domain (green). **c** Enrichment analysis was conducted using STRING and significant expressed categories (FDR < 0.05) are indicated

A total of 59 genes were found differentially expressed with increased expression of 16 genes and decreased expression of 43 genes in the Cp13 mutant under iron restriction (Fig. 2b). Again, a higher number of differentially expressed genes were assigned to term cellular component (GO:0005575) (Fig. 4a). Similar to the wild-type strain, the cellular component category included the induction of genes involved in the transportation of hemin (*htaA*, *htaC*, *htaF*, *htaG*, *Cp_3070*, and *Cp_3075*) and the down-regulation of generation of precursor metabolites and energy (*qcrB, qcrC, ctaE, ctaF, ctaC,* and *qcrA)*. Regarding energy metabolism, the *ndh* gene was only up-regulated in the Cp13 mutant and encodes an iron-free type II NADH dehydrogenase. The copper containing NADH II and the cytochrome *bd* oxidase complex are responsible for the generation of a less efficient proton gradient during micro-aerobic growth, which in turn generates ATP through ATP synthase [26, 27]. The Cp13 mutant also exhibited increased expression of DEGs assigned to the GO term DNA binding (GO:0003677), genes *hrrA*, *whiB*, *ripA, glpR,* and *cspA*. The ArsR family repressor of iron-sulfur cluster biogenesis gene (*sufR*) and the WhiB family transcription regulator were differentially expressed only in the Cp13 mutant (See Additional file 3). The up-regulated *whiB4* gene encodes a protein with a redox-sensitive iron-sulfur cluster that is potentially associated with the regulation of genes involved with the oxidative stress response of *C. glutamicum* and *M. tuberculosis* [28, 29]. This iron-sulfur containing transcription factor was also implicated in the intracellular replication of *Mycobacterium marinum* in macrophages and diminished virulence [30]. Furthermore, overexpression of the *whiB4* gene (*whcA* homologous) is strongly associated with slow growth in *C. glutamicum* cells [28]. Strikingly, while the ribosome (GO:0005840) and translation (GO:0006412) categories were composed of down-regulated genes in the wild-type strain, the genes *rpsO*, *rpsB*, *rpsP*, and *rspT*, were all induced in the Cp13 mutant (Fig. 4a). See Additional file 4: Table S8 for the full listing of GO terms identified. The genes encoding ribosomal proteins of the small 30S ribosome subunit (genes *rpsB*, *rpsO*, *rpsP*, *rpsT)* were identified as up-regulated by > 1.5-fold difference (See Additional file 3). The PPI network of the Cp13 mutant contained 56 nodes and 94 edges with a confidence score of ≥0.4 (Fig. 4b/Additional file 5: Table S10). No significant node genes (more than 10 connections/interactions) were identified in the network. GO enrichment analysis confirmed the down-regulation of the oxidative phosphorylation (KEGG 00190, *p* adjusted value = 5.17e-06), and ribosome (KEGG 03010, *p* adjusted value = 0.01) pathways, as well as the induction of genes encoding proteins with a HtaA hemin-binding domain (PFAM PF04210, *p* adjusted value = 4.62e-05) (Fig. 4c).

### The main core iron restriction response

**Fig. 5:**
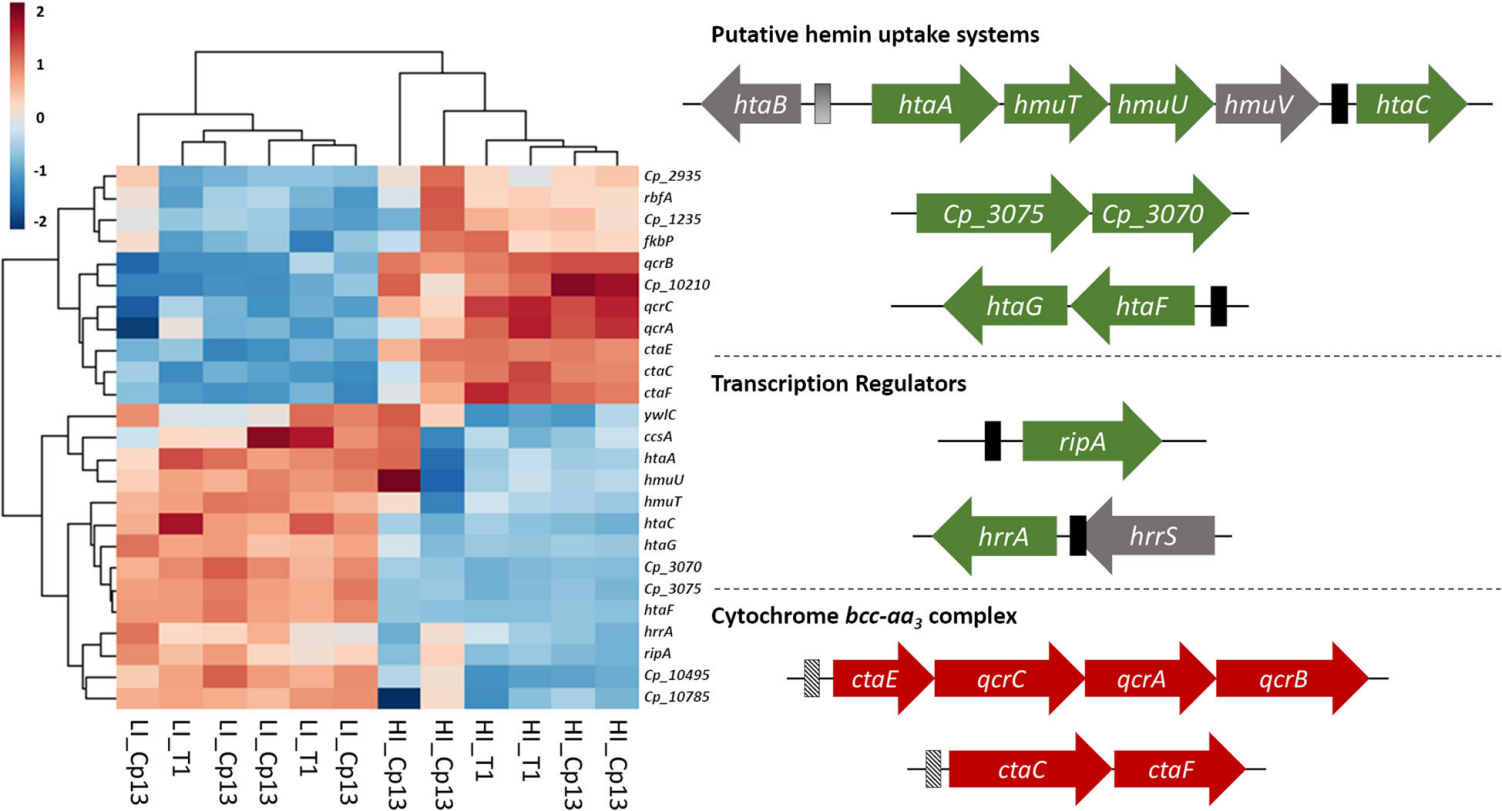
Expression pattern of commonly expressed genes identified between the T1 strain and the Cp13 mutant. Hierarchical clustering of the 25 DEGs commonly expressed between the strains was used to identify the pattern of expression of these genes. *Rlog*-transformed normalized counts in the heatmap were clustered based on Euclidean distance and show 25 DEGs consistently up- or down-regulated in the T1 strain and Cp13 mutant. Columns represent sample identification in relation to experimental condition and strains. Representative gene clusters identified between both strains are indicated highlighting the clusters involving high-affinity hemin-binding acquisition systems, transcription factors and genes encoding the cytochrome *bcc-aa3* super complex of the respiratory chain. Up-regulated genes are shown as green arrows; down-regulated genes are shown as red arrows and genes with unchanged expression are shown as gray. Black solid bars represent DtxR predicted regulatory binding sites and striped-bars represent GlxR regulatory binding sites

Hierarchical clustering of the commonly expressed genes in both strains revealed a similar pattern of gene expression between wild-type and mutant strains, with all 25 DEGs being consistently up- or down-regulated in the T1 strain and Cp13 mutant (Fig. 5). We believe that these DEGs may contribute directly to the homeostasis of iron in *C. pseudotuberculosis.* Characterization of these overlapped DEGs revealed 5 genes encoding conserved hypothetical proteins, hemin transport proteins (*htaA*, *htaC*, *htaF*, *htaG*, *Cp_3070*, *Cp_3075, hmuT* and *hmuU* genes), protein subunits of the oxidative phosphorylation process (*qcrCAB* and *ctaCEF* genes), transcriptional regulators (*hrrA* and *ripA*), cytochrome c biogenesis protein A (*ccsA*), threonylcarbamoyl-amp synthase (*ywlc*), peptidyl-prolyl cis-trans isomerase (*fkbp*), and Ribosome-binding factor A (*rbfA*) (Fig. 5). Interestingly, these results confirmed the induction of genes with an enriched HtaA domain and the down-regulation of the oxidative phosphorylation process.

Under iron limitation, we observed an up-regulation of genes encoding surface exposed proteins with an enriched high-affinity HtaA hemin binding domain associated with hemin ABC-type transporters. Overall sequence identity of the hemin-binding proteins is low among *Corynebacterium* species [31]; however, the proteins encoded by the *htaA*, *htaC*, *htaF*, *htaG* and *Cp_3070* genes were all identified as having at least one HtaA domain of approximately 150 amino acids. In *C. pseudotuberculosis*, the *htaA* gene appears to be organized in a single operon with the *hmuTUV* genes, while the *htaFG* and the *Cp_3075*-*Cp_3070* genes appear to be organized in two distinct two-gene operons in the genome. Notably, the *hmuT* (hemin binding protein) and *hmuU* (permease) genes of the hemin utilization (*hmu*) operon were among the up-regulated genes in response to low iron availability in both strains (Table 1).

**Table 1:** Differentially expressed genes identified in the T1 wild-type and Cp13 mutant strain of *C. pseudotuberculosis*

*Gene/Locus ID*	Product	LI vs HI Log(2)Fold-change		LI vs HI Fold-change		*p-*value*	
		T1	Cp13	T1	Cp13	T1	Cp13
*Cp1002B_95*	Hypothetical protein	−0.7619 (DOWN)	−0.5325 (UNC.)	0.59	0.69	0.000814	0.019094
*sdcS*	Sodium-dependent dicarboxylate transporter	−1.2318 (DOWN)	−0.5604 (UNC.)	0.43	0.68	6.21E-16	0.011373
*Cp1002B_1235*	Hypothetical protein	−1.1201 (DOWN)	−0.9908 (DOWN)	0.46	0.50	0.002796	0.000258
*ccsA*	Cytochrome c biogenesis protein ccsa	0.6175 (UP)	0.9430 (UP)	1.53	1.92	0.022829	0.002895
*htaA*	Cell-surface hemin receptor	0.9624 (UP)	1.4327 (UP)	1.95	2.70	2.66E-12	0.000128
*hmuT*	ABC transporter substrate-binding protein	0.8922 (UP)	1.6025 (UP)	1.86	3.04	1.19E-11	5.47E-05
*hmuU*	Hemin import atp-binding protein	0.5849 (UP)	1.0666 (UP)	1.50	2.09	3.31E-05	0.005646
*htaC*	Hypothetical protein	2.7936 (UP)	2.8902 (UP)	6.93	7.41	9.8E-66	3.33E-11
*Cp1002B_2935*	Transglycosylase associated protein	−1.3034 (DOWN)	−0.8085 (DOWN)	0.41	0.57	6.46E-15	3.39E-05
*hrrA*	DNA-binding response regulator	0.7473 (UP)	1.9853 (UP)	1.68	3.96	3.02E-06	4.02E-05
*Cp1002B_3070*	Hypothetical protein	3.0864 (UP)	4.5174 (UP)	8.49	22.90	1.74E-34	3.62E-26
*Cp1002B_3075*	Hypothetical protein	3.3487 (UP)	4.8177 (UP)	10.19	28.20	1.71E-47	2.43E-32
*Cp1002B_3725*	FUSC family protein	0.4215 (UNC.)	0.5911 (UP)	1.34	1.51	0.004667	2.37E-05
*Cp1002B_4550*	Antimicrobial peptide ABC transporter	−0.6302 (DOWN)	−0.4877 (UNC.)	0.65	0.71	0.003592	0.003453
*htaF*	Cell-surface hemin receptor	1.7558 (UP)	2.8668 (UP)	3.38	7.29	4.16E-27	2.69E-14
*htaG*	Uncharacterized protein htac	1.4734 (UP)	2.5507 (UP)	2.78	5.86	3.62E-24	0.002598
*ctaC*	Cytochrome c oxidase subunit II	−1.3075 (DOWN)	−1.0670 (DOWN)	0.40	0.48	5.17E-13	1.06E-06
*ctaF*	Cytochrome c oxidase polypeptide 4	−1.1573 (DOWN)	−1.0888 (DOWN)	0.45	0.47	7.95E-11	1.08E-07
*ctaE*	Cytochrome c oxidase subunit III	−1.2057 (DOWN)	−1.3813 (DOWN)	0.43	0.38	9.09E-10	7.24E-11
*qcrC*	Ubiquinol-cytochrome C reductase cytochrome C	−1.5080 (DOWN)	−1.3603 (DOWN)	0.35	0.39	1.19E-14	1.02E-09
*qcrA*	Ubiquinol-cytochrome c reductase iron-sulfur	−1.2355 (DOWN)	−1.1237 (DOWN)	0.42	0.46	7.6E-05	0.000568
*qcrB*	Ubiquinol-cytochrome C reductase cytochrome B	−1.2774 (DOWN)	−1.2865 (DOWN)	0.41	0.41	4.7E-13	6.48E-07
*Cp1002B_5915*	Hypothetical protein	0.5118 (UNC.)	1.0689 (UP)	1.43	2.10	0.006998	1.59E-11
*rbfA*	Ribosome-binding factor A	−0.6969 (DOWN)	−0.6192 (DOWN)	0.62	0.65	0.00192	0.002598
*Cp1002B_8515*	Hypothetical protein	0.4077 (UNC.)	0.6311 (UP)	1.33	1.55	0.039677	0.01409
*ywlC*	Trna threonylcarbamoyladenosine biosynthesis protein ywlc	0.6784 (UP)	0.6002 (UP)	1.60	1.52	0.016627	3.39E-05
*ripA*	HTH-type transcriptional repressor of iron protein A	1.0919 (UP)	1.2883 (UP)	2.13	2.44	1.193E-12	6.07E-13
*rpfB*	Resuscitation-promoting factor rpfb	0.3968 (UNC.)	0.7641 (UP)	1.32	1.70	0.039677	1.58E-07
*fkbP*	Peptidyl-prolyl cis-trans isomerase, FKBP-type	−0.9806 (DOWN)	−0.6872 (DOWN)	0.51	0.62	0.000242	0.013233
*Cp1002B_10210*	Substrate-binding protein	−2.4417 (DOWN)	−1.2848 (DOWN)	0.18	0.41	2.89E-17	1.28E-07
*Cp1002B_10495*	Hypothetical protein	2.3140 (UP)	2.6553 (UP)	4.97	6.30	3.14E-62	2.66E-14
*Cp1002B_10785*	Hypothetical protein	1.8433 (UP)	2.5687 (UP)	3.59	5.93	2.63E-17	3.23E-32

Log (2) Fold- change values correspond to the average normalized count ratio between LI (low iron) media and HI (high iron) media for each individual strain. *p-values were adjusted using the Benjamin and Hochberg false discovery rate (FDR) approach. Genes with *p*-values < 0.05 and a base-2 log-ratio ≥ 0.5849 (i.e., ≥ 1.5-fold) or ≤ −0.5848 (i.e., ≤ 0.66-fold) in relation to the HI media were considered as differentially expressed. Gene regulation is based on the LI:HI ratio expression value: UP, up-regulated (log (2) fold-change ≥0.5849); UNC. unchanged (− 0.5849 < log (2) fold-change < 0.5849); DOWN, down-regulated (log (2) fold-change ≤ − 0.5849)

Generally, low levels of iron are expected to activate a cellular iron restriction response involving the down-regulation of genes encoding key iron/heme proteins of metabolic processes, resulting in a state of low metabolic activity in which bacteria can adapt to the hostile iron-poor environment [32]. Collectively, iron restriction resulted in the down-regulation of genes encoding protein subunits of metabolic complexes involved in energy metabolism, with the down-regulation of genes associated with the oxidative phosphorylation process (KEGG 00190) and the TCA cycle. In both strains, we identified two gene clusters of the cytochrome *bcc-aa*_*3*_ supercomplex, Ubiquinol-cytochrome C reductase – *qcrCAB* operon and cytochrome oxidase c – *ctaCDEF* genes, with down-regulated genes subunits *qcrA*, *qcrB*, *qcrC*, *ctaC*, *ctaE and ctaF* (Table 1). The *ctaE* gene is located adjacent of the *qcrCAB* genes clustered in the order *ctaE-qcrCAB*, suggesting that this gene could be transcriptionally regulated with the *qcrCAB* operon. In actinobacteria, the *ctaE* gene encodes subunit III of the cytochrome aa_3_ oxidase, the *qcrCAB* genes encode the three subunits of the cytochrome *bcc* complex and the *ctaCDF* genes encode subunits (II, I and IV) of the cytochrome *aa*_*3*_ oxidase complex (complex IV), forming a respiratory super complex chain in many high-GC Gram-positive bacteria [33, 34]. The proteins in the *bcc-aa*_*3*_ complex contain four redox prosthetic groups, formed by two heme *b* groups (QcrB), two *c*-type heme (QcrC), a 2Fe-2S cluster (QcrA) and two heme *a* groups (CtaD) in the *aa*_*3*_ oxidase molecules [33, 35]. Taken together, these results indicate that iron restriction hindered the aerobic electron transport chain in both wild-type and mutant.

The transcriptomic analyses showed the upregulation of two well-described iron-dependent TFs amongst the differentially up-regulated genes in both strains: the regulator HrrA of the two-component system (TCS) *hrrSA* and the RipA repressor (Fig. 5), which are known to be regulated by the availability of iron in other corynebacteria [20, 21]. The *hrrA* gene was up-regulated by a 1.6-fold and 2-fold difference in the T1 and Cp13 strains, respectively (Table 1). The two-component system (TCS) are ubiquitous bacterial systems composed of two proteins: a membrane-associated sensor histidine kinase (HK) and a response regulator (RR), which detect specific environmental signals allowing bacteria to regulate osmolarity, nutrient acquisition, modulate gene expression and modify protein interaction [36]. *C. pseudotuberculosis* encodes 8 two-component systems, but little is known about their function. The DNA-binding response regulator HrrA of the *hrrSA* system in *C. pseudotuberculosis* shares high amino acid sequence identity with the homologs HrrA regulator of *C. diphtheriae* (91%) and of *C. glutamicum* (83%) (Additional file 6: Figure S9). In *C. diphtheriae* and *C. glutamicum,* the *hrrSA* system is responsible for the activation of the heme oxygenase (*hmuO)* promoter and for the repression of genes involved in heme homeostasis [21, 36, 37]. The *hrrSA* system has also been shown to regulate the expression of the *ctaE-qcrCAB* and *ctaCF* genetic clusters [36]. In *C. glutamicum*, a *hrrA* mutant showed a strong decrease in the expression of the *qcrCAB* and *ctaCEF* genes in the presence of heme, which suggests an *hrrSA* regulation. In addition, genes belonging to both clusters were also shown to be down-regulated in the presence of iron in *ΔhrrA* mutant [36]. Correspondingly, the *ripA* gene was up-regulated by a 2-fold difference in the T1 strain and by a 2.4-fold in the Cp13 mutant strain (Table 1). It has been demonstrated that under iron limitation, the RipA protein reduces cellular iron demand by acting as a repressor of several genes encoding iron-containing proteins, such as aconitase and succinate dehydrogenase [20, 22, 23]. These results are in accordance with the down-regulation of the iron-sulfur proteins encoded by the *sdhCAB* genes in the T1 strain (Additional file 3: Table S5).

### DEGs regulatory networks and genomic islands (GIs) prediction

**Fig. 6:**
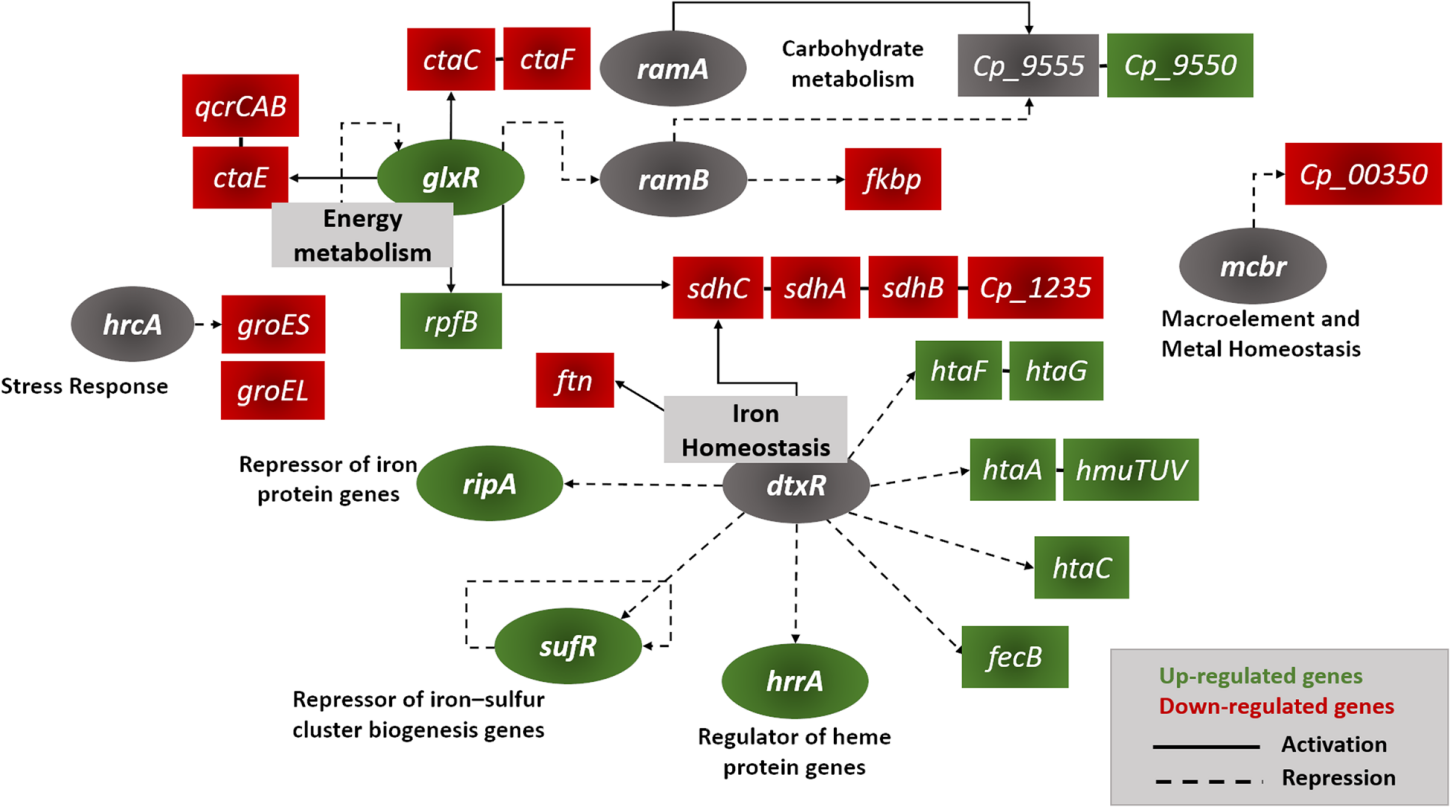
Gene network of the DEGs between wild-type T1 strain and Cp13 mutant. Networks shown involve DEGs identified in the transcriptional profile of both strains. TF genes are shown as circles. The colors represent the type of expression pattern and it is consistent with data in Table 1. Green indicates up-regulated genes, red indicates down-regulated genes and gray indicates genes with no difference in fold-change expression. Black solid lines represent transcriptional activation of the target gene and black dashed lines represent transcriptional repression of the target gene. Connected genes represent operons

**Fig. 7:**
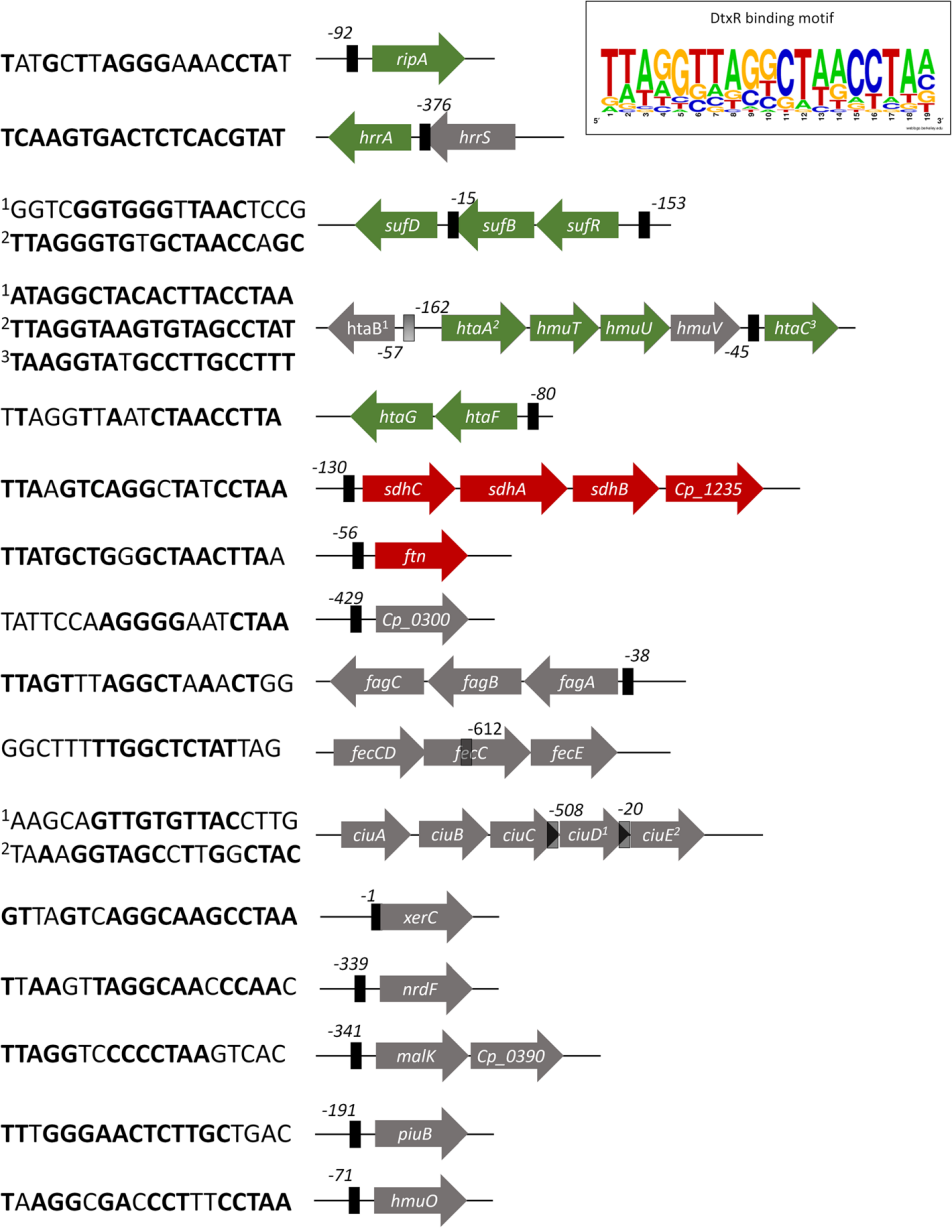
Genetic map of the target genes identified as having a putative iron-DtxR regulated binding site. Conserved residues in relation to the reference are indicated in bold. DtxR target site prediction was based on orthologous target and experimentally confirmed sites of DtxR orthologous identified in taxonomically closely related species: *C. glutamicum* (NC_003450), *C. diphtheriae* (NZ_LN831026) *and M. tuberculosis* (NC_000962). Upregulated genes are shown as green arrows; downregulated genes are shown as red arrows and genes with unchanged expression are shown as gray. Detected DtxR binding sites are indicated as black boxes upstream the genes coding regions. The 19-bp consensus sequence of the DtxR-binding site of *C. pseudotuberculosis* is shown as DNA sequence logo

To identify genes that are most relevant to iron hemostasis and bacterial adaptation to iron restriction, we constructed a transcriptional regulatory network using the DEGs identified in our transcriptome assays. The *C. pseudotuberculosis* network was constructed by using homology-based transference of conserved regulatory interactions, which combine a TF-to-target search with a conserved regulatory binding site prediction of known TFBS from taxonomically closely species (*C. diphtheriae*, *C. glutamicum* and *M. tuberculosis*). TFBS of homologous TFs were used to create an HMM profile that was used to search for the presence of regulatory sites upstream of the coding regions of target homologous genes in the *C. pseudotuberculosis* genome. This approach yielded 187 interactions with 21 transcriptional factors and 119 transcriptional binding sites. Based on these results, we analyzed the set of DEGs to identify specific networks regulated under iron restriction and identified the top two regulons to be controlled by the global regulator protein (GlxR) and the master regulator of iron metabolism protein (DtxR - diphtheria toxin repressor) (Fig. 6). Both GlxR and DtxR regulons are connected by coregulation and are involved in the regulation of 25 DEGs, which are mainly associated with energy metabolism and iron homeostasis (Fig. 6). The DEGs GlxR regulon is formed by 14 down and up-regulated genes involved in energy metabolism, stress response and resuscitation. Regulatory GlxR sites were identified controlling the expression of the down-regulated genes in the *ctaEqcrCAB*, *ctaCF*, *sdhCAB-Cp1235* operons and upstream the coding region of the up-regulated *rpfB* gene (Fig. 6). A regulatory GlxR binding site was also identified upstream the transcriptional regulator RamB, indicating that GlxR could be cross-regulating the expression of the down-regulated *fkpb* gene under iron restriction (Fig. 6). Interesting, we have also identified a coregulatory interaction between the RamB and RamA transcription factors and the up-regulated *Cp_9550* gene (Fig. 6). In *C. glutamicum*, both RamA and RamB act controlling the utilization of different carbon sources and metabolic adaptation to specific nutritional conditions [38]. A regulatory site was also detected upstream of the *glxr* gene, suggesting that the synthesis of GlxR might be controlled by negative autoregulation (Fig. 6). DtxR was the most connected node of the network, involved in the regulation of 15 DEGs, including 2 regulatory genes (Fig. 6). In *C. pseudotuberculosis*, the homologous DtxR gene encodes a protein of 226 amino acids with over 79% of sequence similarity to the DtxR of *C. diphtheriae* and *C. glutamicum,* and 70% to the IdeR of *M. tuberculosis.* The DtxR metal-ion-dependent protein is composed of an N-terminal DNA binding domain and a flexible C-terminal domain with two metal-binding sites [39]. Although the breadth of the gene expression regulation may vary among the target genes in *Corynebacterium* species [40, 41]; overall, in the presence of ferrous iron, DtxR mediates gene expression regulation by binding directly to a 19-bp operator sequence in iron-regulated promoters, thus preventing the binding of RNA polymerase. In contrast, in an iron-deficient environment, DtxR is unable to bind Fe^2+^ and the repression of target regions is released [42]. 20 putative DtxR binding sites with the consensus sequence TTAGGTTAGGCTAACCTAN were identified in the genome of *C. pseudotuberculosis* (Fig. 7), which might be involved in the regulation of over 40 genes encoding proteins involved in iron acquisition, storage, metabolism, and transcriptional regulation. Confirming previous findings, putative regulatory DtxR sites were identified upstream of the coding regions of genes related to iron acquisition and transportation (*htaAhmuTUV*, *htaC* and *htaF*); iron storage *(ftn)* and the ferric citrate transport system *(fecB)* (Fig. 7*)*. Although gene clusters involved in siderophore acquisition were not identified as differentially expressed under iron limitation, regulatory site motifs were found in the intergenic region between the *fagD* gene and the *fagABC* cluster, upstream the *fecCDE (CD)* operon and upstream the coding regions of the *ciuE* and *ciuD* (of the *ciuABCDEF* cluster) genes (Fig. 7) [14, 43]. The proposed DtxR network revealed a complex hierarchical cross-regulatory structure, with DtxR negatively controlling two other transcription regulators identified as differentially expressed in our results: the *ripA* and *sufR* (Fig. 6). The *sufR* gene is a metalloregulator of the ArsR protein family responsible for the repression of the *suf* gene cluster, which is involved in the biogenesis of iron-sulfur clusters in bacteria [44]. The RipA protein is part of the AraC family of transcription regulators and it has been demonstrated that under iron limitation, the DtxR repression of the *ripA* gene is relieved and the transcription of RipA protein reduces cellular iron demand by acting as a repressor of several genes encoding iron-containing proteins, such as aconitase and succinate dehydrogenase [22, 23]. Although no homologous regulatory *ripA* site was found upstream the *sdh* genes, previous results have shown that the operon is actively repressed by the binding of the *ripA* repressor [22]. In our prediction, regulatory binding sites for DtxR and GlxR were identified in the promoter region of the *sdhCAB.* The least connected TFs of the network are the *mcbr* and the *hrcA* regulators, which are involved in metal homeostasis and stress response (Fig. 6) [20].

Genomic islands (GIs) are large DNA sequences horizontally transferred between microbial genomes. Genomic island prediction was carried out using GIPSy and identification was based on the presence of signature features common to the exogenous fragment, such as anomalous C-G content, codon-usage deviation, the presence of transposase, tyrosine and serine recombinase genes and flaking direct repeats [45]. These regions often carry genes associated with antibiotic resistance, metabolism, virulence and adaptation factors, which in turn enhance bacterial survival under adverse conditions [46]. We have identified 7 putative genomic islands harboring 8 DEGs, including three genes encoding conserved hypothetical proteins (*Cp_10540, Cp_10545*, *Cp_10265)*, 4 hemin-binding transport proteins (*Cp3070-Cp3075, htaFG* genes)*,* and 1 acetolactate synthase *(ilvb1)* (Additional file 6: Table S11)*.*

## Discussion

In pathogenic bacteria, iron acquisition is an intrinsic part of the proliferation and host colonization process [47]. Upon entering the host, pathogenic bacteria encounter a period of iron starvation and must scavenge enough iron in order to successfully establish infection [32]. To compensate for the low iron availability tightly controlled by the host, these pathogens employ sensitive transcriptionally regulated iron-dependent systems directly associated with the bioavailability of the metal [32, 47]. Describing these mechanisms is key to understand how *C. pseudotuberculosis* adapts and survives within the host and, most importantly, how it causes disease. To investigate the gene expression of *C. pseudotuberculosis* under iron restriction a high-throughput RNA sequencing (RNA-seq) transcriptome profiling was used to characterize the differential gene expression between low iron (LI) and high iron (HI) cultures (LI vs HI) of the wild-type T1 strain. Additionally, since high-affinity iron scavenging siderophores seem to play a pivotal role in the procurement of iron in bacteria, a similar transcriptome analysis was conducted using the siderophore-defective Cp13 mutant. To gain more insight into the *C. pseudotuberculosis* adaptation to iron restriction, we conducted a homology-based in vitro prediction of TFBS to coordinate the results of our differentially expressed gene with candidate iron-regulated TFs.

### Wild-type T1 and Cp13 mutant transcriptional response to iron restriction

In this study, differential expression analyses were conducted independently in a wild-type strain and mutant of *C. pseudotuberculosis*, with 77 genes identified differentially expressed in the wild-type T1 strain, and 59 DEGs identified in the Cp13 mutant under iron restriction conditions (Fig. 2). Interestingly, less than 2.7% of the genes were affected in the Cp13 mutant and less than 3.6% of the genes were affected in the wild-type strain under iron restriction. While it appeared that disruption of the *ciuA* had little impact on the transcriptome of the Cp13 mutant, the majority of DEGs were preferentially repressed in the T1 strain (20 up-regulated and 57 down-regulated) and induced in the Cp13 mutant (43 up-regulated and 16 down-regulated) (Fig. 2c). A comparison of the dataset of DEGs between the wild-type T1 strain and the Cp13 mutant identified 52 genes uniquely regulated in the T1 strain, while 34 genes were expressed only in the Cp13 mutant under iron restriction. For the Cp13 mutant, these unique DEGs represent specific transcriptional gene changes associated with the disruption of the *ciuA* gene. Interestingly, we have also identified 25 consistently regulated DEGs overlapping, suggesting that the transcriptional responses induced by iron restriction are substantially similar between the wild-type T1 strain and Cp13 mutant.

### *C. pseudotuberculosis* Cp13 mutant strain exhibits reduced growth and specific transcriptional response under iron restriction

As previously described, the *ciuA* Cp13 mutant was generated by an in vivo insertional mutagenesis of the reporter transposon-based system TnFuZ within the genome of the virulent T1 strain, resulting in the disruption of the *ciuA* gene [12]. In *C. pseudotuberculosis*, the *ciuA* gene appears to be organized into an operon (*ciuABCD*) with high protein similarity to a lipoprotein component of the iron-siderophore transport system in other corynebacteria, where mutations in *ciuA* greatly reduce iron uptake and the ability to use siderophore as an iron source [14]. In this study, while the Cp13 mutant exhibited diminished growth in low iron medium, the mutant was able to achieve cellular density comparable to the wild-type T1 strain in the high iron medium suggesting the presence of redundant iron uptake systems in addition to the *ciu* system [47]. However, it is important to notice, similar to the *C. diphtheriae ciuA* mutant [14], that although the disruption of the *ciuA* gene was not able to fully abolish growth of the Cp13 mutant (Fig. 1), the significant reduction in growth in low iron medium relative to the wild-type strain indicates that CiuA protein could be required for the sufficient acquisition of iron and normal growth of the mutant strain. Moreover, further analysis of the 34 unique differentially expressed genes in the Cp13 mutant indicated a shift in metabolic activity of the mutant favoring ATP production through iron-independent pathways. To compensate for the down-regulation of genes encoding heme-containing proteins of the respiratory *bcc-aa*_*3*_ complex, the up-regulation of the type II NADH dehydrogenase (*ndh*) in the Cp13 mutant suggests a shift of electron flow from the *bcc-aa*_*3*_ complex to the *bd* complex*,* favoring the use of iron-free proteins of the micro-aerobic chain under iron restriction. This is in accordance with previous reports that have shown that this respiratory micro-aerobic chain is predominant when iron is scarce [48]. Nonetheless, this shift in metabolic activity in the Cp13 mutant could provide a mechanism for the bacterium to compensate the restriction of intracellular iron demand and promote the production of ATP, which is required for pivotal biosynthetic processes, such as protein synthesis. Since translation is an energy-consuming process, there is a strong connection between ATP availability and protein synthesis [49]. Under most growth conditions, the rate of protein synthesis is strongly associated with efficient ribosome biogenesis, which is in turn tightly coupled with the energy cell production and nutrient availability [49]. In the Cp13 mutant, we observed up-regulation of a set of genes required for protein synthesis whose expression is normally down-regulated when under nutritional restriction [49]. However, these results were not unexpected given the substantial number of up-regulated genes identified in the set of DEGs of the Cp13 mutant compared to the wild-type strain. Contrasting to the downregulation of the resuscitation-promoting factor A (*rpfA*) gene in the wild-type T1 strain another transcriptional change associated with the disruption of the *ciuA* gene was the up-regulation of the resuscitation-promoting factor B (*rpfB*) gene in the Cp13 mutant (See Additional file 3). These Rpf proteins contain a highly conserved Rpf domain with peptidoglycan-hydrolyzing activity [50, 51]. In *M. tuberculosis*, the Rpf proteins act as bacterial growth factors that stimulate the regrowth of dormant mycobacteria cells, contributing to *M. tuberculosis* persistence and virulence inside the host [52]. The role of these independently regulated genes is still inconclusive with no significant phenotype being assigned to a specific *rpf* gene, suggesting a functional redundancy between these genes [53]. How exactly the iron acquisition *ciu* cluster and the *rpf* genes are associated is not immediately clear; however, the up regulation of the *rpfB* gene in the Cp13 mutant indicates an attempt to grow in a media unconducive for optimal replication.

#### Siderophore gene regulation under iron restriction

We selected the Cp13 mutant as an iron limitation model hoping to illuminate how this bacterium copes with iron stress, and to understand the role that the siderophore *ciu* cluster plays in iron acquisition. Unexpectedly, we were unable to address the involvement of siderophores in the acquisition of iron by *C. pseudotuberculosis*. Siderophore synthesis has a pivotal role in iron acquisition for many pathogenic species [47]. For these pathogens, previous results have highlighted the importance of CiuA protein in virulence and pathogenesis [14, 16, 47]. Surprisingly, none of the genes believed to encode proteins involved in siderophores biogenesis and uptake (*ciuABCDEF* and *fagABC*-*fagD* clusters) were identified as differentially expressed in the wild-type T1 strain, or in the Cp13 mutant strain. Contradictorily, regulatory DtxR sites were identified upstream the genes *ciuD* and *ciuE* of the *ciuABCDEF* genetic cluster and upstream the *fagA* gene of the *fagABC* cluster (Fig. 7), indicating that these clusters are probably regulated by DtxR and iron. This could suggest that similar to other iron-regulated genes, these putative siderophores clusters must be actively repressed by DtxR under sufficient iron availability. However, under iron limitation, gene expression is likely to require additional regulatory pathways [21, 54–56]. This hypothesis was first proposed by Billington et al. (2002) [15], where the expression of the siderophore ABC-type transporters encoded by the *fag* genes was only observed in vivo*,* thus suggesting that unknown host factors could play an important role in the expression of genes involved in siderophore synthesis and uptake. Although no differential expression was observed, the *ciu* cluster appears to be essential for the growth and the bacterial adaptive responses under iron restriction. However, the data on siderophore production and acquisition in *C. pseudotuberculosis* is still circumstantial and further studies must be conducted in order to clarify the role of high affinity siderophores in the acquisition of iron in *C. pseudotuberculosis*.

### *C. pseudotuberculosis* iron restriction response

In this study, comparison of both transcriptome profiles datasets allowed us to identify 25 differentially expressed genes overlapping between wild-type and mutant, and 52 uniquely regulated genes in the wild-type T1 strain under iron restriction. Since low iron availability in the host represents major stress for bacterial pathogens and is considered a signal that leads to significant changes in cell processes [10, 32, 47], these genes may be clues into the survival, adaptation and virulence of *C. pseudotuberculosis*. Gene enrichment analysis of the main core iron restriction response genes identified in both mutant and wild-type strain revealed significant representation of genes associated with the acquisition and transportation of heme and the oxidative phosphorylation process, as well as two known iron-regulated TFs (*hrrA* and *ripA*). Consistently, ontology analysis of the uniquely DEGs regulated in the T1 strain revealed significant representation of genes associated with energy metabolism, biosynthetic process and transcription regulation (Fig. 3a). The *C. pseudotuberculosis* response to iron restriction appears to have unique and shared gene expression components that is also reflected in the iron acquisition mechanisms and metabolic activity of the bacterium as described below.

#### Iron limitation increased the transcription of putative hemin acquisition systems

The acquisition of iron bound especially to heme is very common in pathogenic bacteria and for many Gram-positive species, heme represents the preferred iron source, accounting for approximately 70–75% of the total available mammalian iron pool [57]. There were 8 (*htaA*, *htaC*, *htaF*, *htaG*, *Cp_3070, Cp_3075* and *hmuTU)* up-regulated DEGs involved with heme uptake systems in both Cp13 mutant and T1 strain, suggesting a relevant role for hemin as iron source. These genes were identified organized in three distinct genetic loci in the chromosome, all three exhibiting a remarkable difference in expression levels between the up-regulated genes in both strains. However, the *htaFG* and the *Cp_3070*-*Cp_3075* genes were strongly up-regulated in the Cp13 mutant, suggesting that the disruption of the *ciuA* gene could have elicited compensatory mechanisms involving the acquisition of iron in the form of hemin in the mutant strain.

Remarkably, the predicted products of the *Cp3075-Cp3070* and *htaFG* loci share striking structural similarities to the hemin and hemoglobin binding proteins CirA-ChtC (DIP0523-DIP0522) and ChtA-ChtB (DIP1520-DIP1519) described in *C. diphtheriae* [58]. In both species, all four genes are arranged in two-gene operons and encode proteins with N-terminal secretion signals, C-terminal transmembrane regions and conserved HtaA domains critical for binding hemin (Additional file 6: Figure S6). Moreover, the Cp_3075 shares 32.7% amino acid sequence identity and 57.8% similarity with CirA, while the Cp3070 protein shares 33% of amino acid identity and 50.4% similarity with the ChtC protein. The *Cp_3070* and *Cp_3075* genes are separated by an intergenic region of 12 nucleotides, indicating that these genes are probably regulated by a single promoter. Similar to the CirA protein of *C. diphtheria*, the Cp_3075 protein lacks a putative HtaA domain, however its strong association with the HtaA-like membrane protein encoded by the *Cp_3070* gene (PPI confidence score of 0.829 in both strains) suggests that this protein is likely involved in the uptake of hemin in *C. pseudotuberculosis*. The HtaF and HtaG proteins of *C. pseudotuberculosis* share 60 and 62% of amino acid sequence identity, 74 and 75.4% sequence similarity with the ChtA and ChtB proteins of *C. diphtheria*, respectively. In this study, both loci were strongly induced under iron limitation, which is consistent with the iron-DtxR regulation observed in the promoter region of the *chtAB* and *cirA-chtC* operons of *C. diphtheriae* [58]. Additionally, a DtxR regulatory site was identified upstream of the start codon of the *htaF* gene that shared 14 conserved residues with the DtxR binding site sequence identified for the *chtA-chtB* operon, confirming that the *htaFG* cluster is iron and DtxR regulated (Fig. 7). Despite being strongly up-regulated in response to iron restriction, we found no significant evidence of a regulatory DtxR sequence upstream of the putative *Cp3075-Cp3070* operon. Nevertheless, it is important to point out that the absence of a regulatory site could be partially attributed to the site prediction methodology, which restricted the detection to highly similar regulatory sites previously identified in orthologous target genes. This is in accordance with the site prediction for the *cirA-chtA* operon, which showed a relatively poor match to the consensus DtxR-binding site, in spite of showing a strong iron and DtxR transcriptional regulation of the *cirA* promotor region [58]. In addition, the *chtA-chtB* operon of *C. diphtheriae* was shown to be flanked by inverted repeats of identical insertion sequence (IS) elements, creating a transposon-associated system. Both the *Cp3075-Cp3070* and the *htaFG* clusters were identified within genomic islands of *C. pseudotuberculosis,* indicating that both clusters were probably acquired by horizontal gene transfer (Additional file 6: Table S11). A closer examination revealed that these putative genomic islands harbor several genes whose products are known to contribute to the virulence of various pathogenic bacteria [59, 60]. Although the contribution of these hemin-binding proteins to the virulence or survival of *C. pseudotuberculosis* remains to be determined, the relation between iron acquisition and virulence was previously reported by Billington et al. (2002) [15], which identified the *fagABC* iron uptake operon located in a pathogenicity island along with the primary virulence factor phospholipase D (Pld). The *C. pseudotuberculosis fagB(C)* mutants showed reduced abscess formation in CLA models, with the in vivo expression of the *fagABCD* genes significantly contributing to the virulence of the strain. The hemin uptake *ChtAB* system could also be implicated in the virulence of *C. diphtheriae*, being found in related strains responsible for the diphtheria outbreak in the 1990s [58, 61].

Interestingly, in both strains under iron limitation, we observed an up-regulation of the genes encoding a complete hemin iron acquisition system formed by the *htaABC* and *hmuTUV* genes [62]. Strong evidence suggests that the cluster of six genes, *htaB-htaAhmuTUV-htaC,* is likely involved in the acquisition and transportation of hemin, sharing substantial sequence similarity with genes encoding hemin uptake systems in other corynebacteria, including *C. diphtheriae* and *C. ulcerans.* An alignment of the *C. pseudotuberculosis* HmuT and HmuU amino acid sequences demonstrates a high level of identity with the Hmu components of *C. ulcerans* and *C. diphtheriae*. We observed a sequence identity of 97 and 98% with HmuT and HmuU of *C. ulcerans* and 81 and 82% of identity with HmuT and HmuU of. *C diphtheriae* (Additional file 6: Figure S7/S8). Moreover, the conserved Tyr233 and Tyr347 residues, related to the binding and stabilization of hemin Fe^3+^ [63–66], were identified in the HmuT amino acid sequence (Additional file 6: Figure S8). In *C. diphtheriae*, it has been proposed that hemin is acquired from hemoglobin by the surface-exposed hemin binding HtaA protein, which sequentially transfers the heme group to the membrane HtaB protein and the substrate binding HmuT protein. The lipoprotein HmuT takes the hemin to the ABC transporters to pump hemin through the cellular membrane [21, 67]. Three distinct putative 19-bp DtxR binding sites were identified upstream of the *htaAhmuTUV* operon and upstream the start codon of the *htaB* and *htaC* genes (Fig. 7), confirming the iron-DtxR regulation of the cluster [62].

#### Iron restriction hinders energy metabolism: control of the intracellular iron demand

Oxidative phosphorylation is the final stage of energy-yielding metabolism in aerobic respiration and represented the most significant enrichment observed across all down-regulated genes identified in both strains, suggesting that iron limitation hinders *C. pseudotuberculosis* aerobic respiration. In both strains, iron restriction resulted in the down-regulation of genes encoding protein subunits of the cytochrome *bcc-aa*_*3*_ super complex (*qcrCAB* operon and the genes *ctaCDEF)* (Fig. 5). Accordingly, most strain-specific genes down-regulated in the T1 strain under iron-restriction encode components of the oxidative phosphorylation process as well as enzymes of the TCA cycle. These down-regulated genes included succinate dehydrogenase iron-sulfur proteins (genes *sdhA*, *sdhB and sdhC*), dihydrolipoamide acyltransferase *(aceF)* and dihydrolipoamide dehydrogenase (*ldh*). The *sdhA*, *sdhB* and *sdhC* genes form the respiratory complex II of the respiratory chain in which succinate reduction acts as a pivotal link between the TCA and the oxidative phosphorylation process [33]. The *sdhCAB* gene cluster is formed by a membrane protein (SdhC) that interacts with ubiquinone and anchors the catalytic domain at the membrane surface. The flavoprotein (SdhA) and iron-sulfur cluster (SdhB) subunits catalyze succinate reduction [68]. The *sdhB* encodes a protein with three prosthetic Fe-Su clusters [35, 69]. The fact that the majority of these down-regulated gene products encode heme or iron-sulfur proteins, suggests that intracellular restriction of iron usage is key to the iron starvation response of *C. pseudotuberculosis.* Moreover, these results corroborate previous observations that there is a decrease in the activity of proteins and protein systems which require heme or iron for their activity under iron-limiting conditions [32, 33, 70, 71]. This is further confirmed by the down-regulation of the *ftn* gene in the T1 strain, which encodes a ubiquitous iron storage protein. Concomitantly, expression of the genes encoding subunits B (*atpF*) and δ (*atpH*) of the F_1_F_0_-complex was also decreased in the T1 strain, indicating reduced ATP synthesis associated with the respiratory pathway. The F_1_F_0_-complex integrates the electrochemical gradient of proton translocation through the membrane for the synthesis of ATP [33]. ATP synthase is encoded by the *atp* operon, which is tightly regulated by cellular ATP requirements and respiratory chain activity. These findings suggest a more pronounced inhibition of aerobic processes in the wild-type T1 strain. In a nutrient-poor environment, a lower metabolic rate has been shown critical for the survival of *M. tuberculosis* inside the macrophages [72]. Consistent with lower metabolic activity, the global regulator GlxR was uniquely up-regulated in the T1 strain under iron restriction. It has been demonstrated that the gene coding for the Cyclic AMP (cAMP) GlxR global transcriptional regulator is involved in the control of over 200 genes in *C. glutamicum* [73] and 80 genes in *C. diphtheriae* [74], as well as the expression of genes involved in carbohydrate metabolism, aerobic and anaerobic respiration, fatty acid biosynthesis, deoxyribonucleotide biosynthesis, cellular stress response and resuscitation [20, 75].

#### Iron-regulated transcription factors

The majority of the differentially expressed genes identified in our analyses were shown to be regulated by DtxR (Fig. 6), which is not unexpected given its role in iron metabolism. DtxR is the key TF regulator of iron homeostasis in corynebacteria and our data showed that DtxR coordinates a complex cross-regulatory interaction involving the up-regulated *ripA* gene, which have been reported to play a role in the expression of genes encoding iron proteins under iron restriction [22, 23, 41]. In addition, iron also appears to control the expression of the DNA-binding regulatory protein HrrA (of the *hrrSA* two-component system), which resulted in the up-regulation of the HrrA regulator in both mutant and wild-type strains. Both, *hrrA* and *ripA* genes, were identified up-regulated in the wilt-type T1 and Cp13 mutant. In *C. glutamicum*, the HrrA was shown to regulate the expression, in an iron-dependent manner, of genes encoding iron and heme-containing protein via DtxR [36, 37]. In contrast, no DtxR binding motif was reported in the promoter region of the *hrrSA* system in *C. diphtheriae*, exposing differences in regulation between *Corynebacterium* species [21]. Comparatively, we also found no significant evidence for a DtxR binding site in the promoter region of the *hrrA* gene. However, given the predicted divergence in DtxR binding sequence within the *Corynebacterium* species, this observation was probably due to the low conservation of the DtxR regulatory site among these two species. To confirm this hypothesis, we aligned the regulatory site of *C. glutamicum* with the in silico predicted site identified in *C. pseudotuberculosis* FRC41 strain by Trost et al. (2010) [40]. As expected, these two species shared 8 out of the 19-bp DtxR binding site predicted for the target *hrrA* gene. Furthermore, we conducted a BLAST search of the Cp13 and T1 strains genomes for the in silico predicted 19-bp DtxR binding site. The DtxR predicted site (TCAAGTGACTCTCACGTAT) was located upstream of the start codon of *hrrA* gene overlapping the coding region of the *hrrS* gene, sharing 19-bp (out of 19) consensus identity with the predicted DtxR binding motif (Fig. 5). Besides the nucleotide diversity, these regulatory DtxR sites were both identified overlapping the *hrrS* gene, which could indicate that only the *hrrA* gene of the *hrrSA* system is regulated by iron and DtxR in *C. glutamicum* [36, 37], and *C. pseudotuberculosis*. In *C. diphtheriae* and *C. glutamicum,* the *hrrA* gene of the *hrrSA* system is responsible for the activation of the heme oxygenase (*hmuO)* promoter and for the repression of genes involved in heme homeostasis [21, 36, 37]. In addition, *hrrA* mutants of *C. glutamicum* showed a strong decrease in the expression of the *qcrCAB and ctaCFE* genes, which could suggest that these genes might be regulated by the *hrrSA* system [36]. In regard to function, many of the DEGs directly regulated by DtxR were related to iron homeostasis as well as other metabolic processes. There is also a regulatory link between the DtxR and GlxR regulons, which appear to positively co-regulate succinate enzymes of the TCA cycle. The coordination of expression by these transcription regulators enable the bacteria to rapidly adapt gene expression levels to specific environmental conditions [20].

#### Proteins synthesis under iron limitation

The ribosome is an essential ribonucleoprotein complex indispensable for growth involved in translation [76]. In this study, many ribosomal protein genes, including the large 50S and small 30S subunits of the ribosome, were identified differentially regulated under iron restriction. Consistently with the energy deficiency, a larger proportion of genes encoding either ribosomal proteins or proteins involved in translation were down-regulated in the wild-type T1, while the genes encoding ribosomal proteins were up-regulated in the Cp13 mutant. Diminished expression of such genes is well documented and likely reflects diminished protein synthesis and growth arrest [49, 76].

## Conclusion

In summary, we identified a total of 77 and 59 transcripts differentially expressed between low iron and high iron cultures of the wild-type parental T1 strain and the Cp13 mutant of *C. pseudotuberculosis*, respectively. Of these total, 25 genes were found consistently up- or down-regulated in the wild-type and mutant strain under iron restriction and could potentially play a role in the intracellular iron homeostasis of *C. pseudotuberculosis*. Functional analysis of these genes demonstrated that the up-regulation of systems involved in the acquisition of iron from hemin and the down-regulation of intracellular iron demand of iron-dependent aerobic respiratory pathways are key to the response of *C. pseudotuberculosis* to iron restriction. Notably, these hemin uptake systems were substantially expressed in the Cp13 mutant, highlighting the importance of hemin and suggesting that *C. pseudotuberculosis* does not restrict iron uptake to a single strategy (for example siderophores), but can adjust iron acquisition accordingly. Some of these hemin uptake genes and many known virulence factors were also identified harbored by genomic islands, corroborating with the association of iron uptake and virulence of pathogenic bacteria. Additionally, two transcription factors (*ripA* and *hrrA*) known to control the expression of genes encoding iron-containing proteins were also up-regulated in both strains. Comparative analysis of the transcriptome profile for the T1 wild-type and Cp13 mutant highlighted the differences between the iron-mediated responses of the two strains involving genes that were directly related to bacterial growth and metabolism. While we were unable to address the expression of siderophore-based iron acquisition systems for this bacterium, our results, although admittedly preliminary regarding the *ciuA* gene, indicate that siderophore-mediated iron acquisition may be required for optimal growth and could be involved in the adaptative gene expression response of *C. pseudotuberculosis* to iron restriction*.* To our knowledge, this is the first study to analyze changes in gene expression between high iron and low iron cultures of two strains of *C. pseudotuberculosis*, providing a sensitive transcriptional analysis of this important pathogen. Our findings contributed to an enhance understanding of the molecular mechanisms involved in the *C. pseudotuberculosis’s* ability to rapidly adapt to the restriction of iron, revealing potential gene targets that can be used in the development of effective therapeutic strategies for CLA.

## Methods

### Bacterial strains

The biovar *ovis* pathogenic wild-type T1 and the *ciuA* iron-acquisition-deficient Cp13 mutant were both obtained from the Laboratory of Cellular and Molecular Genetics (LGCM) at the Federal University of Minas Gerais/Brazil. The T1 strain was isolated from a caseous granuloma found in CLA-affected goats in Brazil (Bahia state) [12]. The Cp13 mutant was generated by Dorella et al. (2006) [12], using the in vivo insertional mutagenesis of the reporter transposon-based system TnFuZ in the strain T1. This system combines a transposable element (*Tn4001*) with a modified Gram-positive bacterium alkaline phosphatase gene (*phoZ*), and it is used for the discovery and mutagenesis of cytoplasm exported proteins. The PhoZ reporter protein is activated upon its exportation from the cytoplasm and insertional mutant colonies are identified by the colorimetric reaction of the alkaline phosphatase with the 5-bromo-4-chloro-3-indolylphosphate substrate [77]. The molecular characterization of the Cp13 mutant showed that the insertion disrupted the *ciuA* gene, which encodes a putative-iron transport binding protein of the *ciuABCD* operon [16].

### Media and growth analysis of *C. pseudotuberculosis* strains

Wild-type T1 and Cp13 mutant strains were grown individually either in the presence of the iron chelator 2,2′-dipyridyl-DIP (Low Iron condition), or without it (High Iron condition). Precultures and main (inocula) cultures for both conditions (LI and HI) were all prepared with Brain Heart Infusion (BHI) broth-0.05% (v/v) Tween® 80 (Sigma-Aldrich) under routine conditions. The iron-chelated BHI medium was prepared with 250 μM of ferrous iron chelator 2,2′-dipyridyl (Sigma Aldrich), which, due to its low aqueous solubility, was prepared with 40% (v/v) of ethanol (0.5 M 2,2′-dipyridyl stock solution) and then was kept refrigerated and protected from light until its use. The 0.5 M 2,2′-dipyridyl stock solution was diluted in sterile water to a concentration of 0.1 M 2,2′-dipyridyl 8% (v/v). To chelate the iron content of the medium, 0.1 M 2,2′-dipyridyl solution was added to fresh BHI broth to a final concentration of 250 μM 2,2′-dipyridyl with 0.02% (v/v) of ethanol. To normalize the effect on gene expression caused by the presence of ethanol, HI cultures were prepared with 0.02% (v/v) sterile-filtered ethanol (final concentration).

Bacterial cultures on iron-chelated (low iron) media and on non-chelated (high iron) media for the wild-type T1 and the Cp13 mutant strains were conducted as follows: precultures were prepared inoculating a single colony of the strains T1 and Cp13 harvested from fresh BHI agar plates. Precultures were incubated overnight at 37 °C (140 rpm). After incubation, precultures were diluted to a start optical density of 0.02 (OD_600_) and inoculated in BHI cultures prepared in the absence (HI) and in the presence (LI) of 250 μM 2,2′-dipyridyl. Cultures were incubated at 37 °C (140 rpm) and bacterial growth was monitored hourly by optical density (OD_600_). The iron-stressed condition was determined by the addition of the iron-chelator, and the stress was confirmed by a reduction of bacterial culture density in the LI medium in comparison to bacteria grown in the HI medium after 6 h and 30 min of incubation. Bacterial viability was assessed by the number of colony-forming units per milliliter (CFU/mL) after the 6h30min incubation. Growth rate was used to estimate the rate of change in the number of bacterial cells in wild-type and mutant cultures cultivated in BHI medium, with and without 250 μM 2,2-dipyridyl (DIP). Cellular density was inferred directly from OD and the rate of bacterial growth was calculated by applying the final and initial OD-values (at t_initial_ = 0 and t_final_ = 6h30min) to the equation: *Growth rate* = (2.303 ∗ (log_10_*ODf*) − (log_10_*ODi*))/(*T*_*final*_ − *T*_*initial*_), according to methods described previously [78]. Growth rate and CFU counts represent averages of three independent assays.

In addition, to assess any possible toxic effect of ethanol under the described culture conditions, bacterial growth and viability were monitored hourly over a 14-h period by optical density (OD_600_) and by the number of CFU/mL after 11 h of incubation using the Cp13 mutant. Comparisons were made by inoculating a starting bacterial preculture at concentration of 0.02 (OD_600_) in BHI media prepared with 0.02 and 0.1% (v/v) of ethanol against a BHI ethanol-free inoculum.

### Statistical analysis

Statistical analysis for the growth assays and bacterial viability under iron limitation and in the presence of ethanol were carried out using the GraphPad Prism v.5.0 software (GraphPad, SanDiego, CA, USA).

### Bacterial transcriptome assays and RNA extraction

In order to identify the transcriptional response of *C. pseudotuberculosis* involved in bacterial persistence under the growth conditions described above, wild-type T1 and Cp13 mutant strains were grown in an iron-chelated media (low iron - LI) and in a non-chelated iron media (high iron - HI). Incubation of individual HI and LI samples were conducted in parallel and independent sets of paired samples (HI and LI conditions) were prepared for each strain. Bacterial growth was monitored hourly by optical density (OD_600_) and total RNA extractions were carried out following 6h30min of incubation. Following the determinate incubation period, 20 mL of each culture (LI and HI) was centrifuged for 10 min at 5000 rpm (4 °C) in 50 mL conical tubes, and the supernatants were discarded. The bacterial pellets were kept on ice and resuspended in 300 μL of Tris/EDTA/SDS (Tris HCl pH 8 50 mM/L–EDTA pH 8 10 mM/L) lysis buffer per 10 mL of used initial culture volume. Cell suspensions were homogenized with 0.1% of SDS and 1 mL of TRIzol® Reagent (Invitrogen™) was added (1 volume per 10 mL of used initial culture). Pellets from each tube were divided into 2 new tubes filled with 1 mm diameter glass microbeads (Bertin Technologies). The cells were mechanically lysed using Prescellys®24, set for 2 cycles (15 s per cycle) at 6200 rpm with 5 s interval between each cycle. The tubes were immediately placed on ice following the lysis. The cooled samples were centrifuged for 10 s at 14,000 rpm and the supernatants were carefully transferred to a new tube for total RNA extraction. Subsequent extraction steps were carried out following TRIzol® Reagent manufacturer’s recommended protocol and total RNA was eluted in 50 μl of RNase free water. Traces of contaminating DNA were eliminated with the Ambion® TURBO DNA-free™ DNase kit (Invitrogen), following manufacturer’s protocol. DNase treated samples were eluted in 50 μl of RNase-free water and RNA quantification was done using NanoDrop NDZ1000 (Thermo Scientific, Wilmington, DE) spectrophotometer. Samples with a 260/280 ratio over 1.9 were considered pure and used in subsequent steps. Samples with a high concentration of total RNA were obtained using a vacuum concentrator and by combining the previously divided samples. RNA quality and integrity of combined samples were verified by the electrophoretic profile of the 16S and 23S rRNA subunits using Agilent™ 2100 Bioanalyzer™ instrument. A total of 14 samples (7 sets of paired samples) were selected for library construction: 3 paired sets (HI and LI condition) of RNA samples from the wild-type T1 strain and 4 paired sets (HI and LI) of RNA samples from the mutant Cp13. All RNA samples were subjected to ribosomal RNA depletion and purification using the RiboMinus™ Bacteria Transcriptome Isolation Kit with the RiboMinus™ followed by use of the Concentration Module (Invitrogen), as described in the manual.

### Library preparation and sequencing

Approximately 5 μg of purified depleted RNA samples were used to prepare 14 individual single-end libraries with the Ion Total RNA-seq v2 for Whole Transcriptome Library kit (Thermo Scientific). Purified RNA was enzymatic fragmented using RNase III. The fragmentation procedure produced a distribution of RNA fragment sizes that ranged from 35 to few thousand nucleotides, with an average size of 100–200 nucleotides. The size distribution and yield of cDNA were assed using the Agilent™ 2100 Bioanalyzer instrument and NanoDrop™ spectrophotometer. Template preparation was carried out with the amplified cDNA and each library template was clonally amplified on the Ion Sphere™ Particles using the Ion OneTouch™ 2 System. Two rounds of sequencing were performed on the Ion Proton™ platform. The library preparation and sequencing steps were performed at the Federal University of Parana/Brazil.

### Quality assessment and differential expression analysis

The output raw data files generated by the Ion proton system were converted to FASTQ-sanger file format using the Torrent Suite Software v. 5.0.5 (Thermo Scientific). The software was used to remove low quality bases at the 3′-end and the ion adaptor sequences. Raw data was then quality checked using the FASTQC tool (Babraham Bioinformatics, Cambridge, UK) [79]. Per base quality dropped significantly at the 3′-end extremity and a sliding window trimming was performed by Trimmomatic; cutting once the average base quality fell within the Phred score of 10 (90% accuracy) [80]. Trimmed reads were again quality checked using the FASTQC tool and mapped to the *Corynebacterium pseudotuberculosis* strain 1002B genome (NZ_CP012837.1), using the Torrent Mapping Alignment Program v3.4.1 (TMAP). Mapping parameters were set as default and the -mapall command was used, combining all 4 available mapping algorithms (map 1, map 2, map 3, map 4) to successfully address the variable read length and the high homopolymer error rate associated with the Ion Torrent technology [81]. The abundance of unambiguously mapped reads to a gene feature in the aligned genome was determined using HTSeq v0.8.0 package [82]. Differential expression analysis was performed with the bioconductor R package DESeq2 v.1.16.1 [19]. In short, using a generalized linear model (GLM) for each gene, the uniquely mapped read counts were modeled as following a negative binomial distribution with mean and dispersion, where the mean is proportional to the concentration of RNA fragments counted, scaled by a size factor normalization, which accounts for differences in sequencing depth between samples [19]. The linear model fit generated coefficients that indicate the overall gene expression strength and the log2-fold change between the low iron condition (LI samples) compared to the reference (HI samples). Pairwise comparisons were conducted in the normalized count data and significance was determined by the Walt test with *p-*value adjusted for multiple testing using the Benjamini and Hochber procedure [19]. Genes with a *p* adjusted value < 0.05 (FDR 5%) were considered to be differentially expressed (DE); these DE genes were ranked by their fold-change and were considered as being up- or down-regulated when there was a minimum 1.5-fold change difference with respect to the HI samples. Regularize-logarithm transformed (*rlog*) data was used to present relative gene abundance using the *rlog* function and applied to measure sample to sample distance by calculating the Euclidean distance between samples and by Principal Component Analysis (PCA). Visual representation was done using the *pheatmap* v.1.0.8 and *ggplot2* v.2.2.1. Heatmaps of the differentially expressed genes identified in both strains were hierarchically clustered using the *rlog* of the normalized count data. Volcano plots were designed for both strains, demonstrating the fold change (log2 ratio) against the statistical significance (−log10 adjusted *p*-value) of the counted genes. A Venn diagram of differentially expressed genes was created to show the numbers of common and distinct genes identified in both strains. Batch effect was addressed in the sequenced data samples of the Cp13 mutant after PCA evaluation and it was attributed to sample processing. Strain-specific data samples were processed in separate groups and batch identification was included into the DESeq2 design formula [19].

### Functional annotation and PPI networks of differentially expressed genes (DEGs)

The biological function of differentially expressed genes was retrieved from the *GOfeat* platform.^1^ In addition, protein-protein interactions (PPIs) analyses were conducted to investigate direct and functional associations between differentially expressed gene products. Protein interactions were constructed using the Search Tool for the Retrieval of Interacting Genes/Proteins (STRING) v.11.0^2^ and were based on the transfer of functional annotation between orthologous genes [24]. Protein interactions with a combined score of > 0.4 and a PPI enrichment value < 0.01 were considered significant. Enrichment analyses were also carried out using the STRING tool and were set to a 1% false discovery rate (FDR). The identification of hub genes, or genes with the highest degree of connectivity within the network, was conducted using Cytoscape – version 3.7.1 [25].

### DEGs regulatory networks and genomic islands (GIs) prediction

A regulatory network consists of interactions between a transcription factor (TF) and its direct target genes, where each regulatory interaction represents the binding of the transcription factor to a specific DNA binding site (transcription factor binding site -TFBS) near its target gene [83]. The *C. pseudotuberculosis* transcriptional regulatory network (TRN) was predicted using regulatory interactions of experimentally confirmed interactions identified in the taxonomically related species: *C. glutamicum* (NC_003450) *C. diphtheriae* (NZ_LN831026) and *M. tuberculosis* (NC_000962). Regulatory data from the model organisms were collected from CoryneRegNet 6.0 [84], RegulonDB [85], CollectTF [86], PRODORIC2 [87], RegPrecise [74], and Abasy Atlas [88]. The methodology combined the detection of orthologous transcription factors and target genes with the identification of conserved binding motifs to reliably transfer transcriptional regulatory interactions between related species [89]. First, ortholog detection was performed using bi-directional BLAST with e-value cutoff of 10^− 35^, which was previously stablished to adequately cluster homologous proteins within the actinobacteria phylum. [90]. Once the homologous target genes were identified, we scanned the promoter region of these genes (or operons) (+ 20 to − 560 relatives to the start of the coding region of the gene) for conservative binding regulatory sites. Experimentally confirmed regulatory sites were used to detect in silico conserved binding motifs in the genome of *C. pseudotuberculosis* using Hidden Markov Model (HMM) profiles, with HMMER software. The HMMER bit score was used to infer the confidence level (high/medium/low) of each predicted binding site, reflecting how well the site sequence matched the profile model [91]. The bit score is calculated as the log-odds ratio score (base two) comparing the likelihood that the query sequence is a significant match to the profile HMM to the likelihood generated by a random model. Predicted regulatory site motifs with a bit score equal or greater than the selected score threshold of 3.45 were considered true positives and indicate high confidence site prediction. Predictions with a bit score between 0 and < 3.45 were considered medium, while negative scores mean that the sequence is probably similar by chance (rated low) [92]. The results of both homology and binding site detection were used with the genomic and regulatory information available from online database literature to create a list of regulatory interactions and to create the networks. These results were filtered to present only networks with connected DEGs. Networks were constructed manually using the DEGs identified in our transcriptome analyses.

Gipsy (Genomic Island prediction software) [45] was used to predict genomic islands (GIs) in the genome of the wild-type T1 strain of *C. pseudotuberculosis* against the closely related nonpathogenic genome of *C. glutamicum* (NC_003450). Comparatively, island prediction was conducted in the reference genome of the *C. pseudotuberculosis* 1002B strain (NZ_CP012837.1). We looked at the genes in the putative predicted islands to see if they were part of the differentially expressed genes that we identified earlier in the analysis.

## Additional files

Additional file 1: **Table S1.** Sequencing statistics. **Table S2.** Total number of processed reads from High Iron (HI) and Low Iron (LI) experimental condition. **Table S3.** Mapping results. **Table S4.** Raw gene counts. **Figure S1.** Sample to sample principal component analysis (PCA) (DOCX 129 kb)

Additional file 2: **Figure S2.** Effect of iron depletion on the growth curve and growth rate of *C. pseudotuberculosis* wild-type T1 strain and Cp13 mutant*.* **Figure S3.** Effects of ethanol on bacterial growth (DOCX 261 kb)

Additional file 3: **Table S5.** Differentially expressed genes identified in the T1 strain in response to iron limitation. **Table S6.** Differentially expressed genes identified in the Cp13 mutant in response to iron limitation (DOCX 63 kb)

Additional file 4: **Table S7.** Table of gene ontology terms assigned to DEGs identified in the T1 strain. **Table S8.** Table of gene ontology terms assigned to DEGs identified in the Cp13 mutant (DOCX 32 kb)

Additional file 5: **Table S9.** Predicted protein-protein interactions of iron regulated genes identified in the wild-type T1 strain. **Table S10.** Predicted protein-protein interactions of iron regulated genes identified in the Cp13 mutant. **Figure S4.** Protein enrichment analysis (DOCX 209 kb)

Additional file 6: **Figure S5.** Alignment of the amino acid sequence of the HtaA domain from protein products of the upregulated genes *htaC*, *htaA*, *htaF*, *htaG* and *Cp_3070* genes of *C. pseudotuberculosis*. **Figure S6.** Structural protein domain characteristics of the HtaA, HtaC, HtaF, HtaG and Cp_3070 proteins. **Figure S7.** Alignment of the amino acid sequences of the HmuU protein between *C. pseudotuberculosis* (Cp), *C. ulcerans* (Cu) and *C. diphtheriae* (Cd). **Figure S8.** Alignment of the amino acid sequences of the HmuT protein between *C. pseudotuberculosis* (Cp), *C. ulcerans* (Cu) and *C. diphtheriae* (Cd). **Figure S9.** Alignment of the amino acid sequences of the DNA-binding regulator *hrrA* of the two-component regulatory system *hrrSA* between *C. pseudotuberculosis* (Cp), *C. glutamicum* (Cg) and *C. diphtheriae* (Cd). **Table S11.** Genomic Island predictions (DOCX 4804 kb)

## Data Availability

The datasets generated and/or analyzed during the current study are available in the Gene Expression Omnibus (GEO) repository under the accession number GSE114125 <https://www.ncbi.nlm.nih.gov/geo/query/acc.cgi?acc=GSE114125>. The datasets supporting the conclusions of this article are included in this article and its supplementary information files.

## Abbreviations

ATP: Adenosine Triphosphate
BHI: Brain heart infusion medium
CFU: Colony-forming unit
CLA: Caseous lymphadenitis
DEGs: Differentially expressed genes
DIP: 2,2′-dipyridyl
EF: Elongation factor
FDR: False discovery rate
GO: Gene ontology
HI: High iron experimental condition
HMM: Hidden Markov Model
KEGG: Kyoto Encyclopedia of Genes and Genomes
LI: Low iron experimental condition
OD: Optical density
PCA: Principal component analysis
PFAM: Protein Family database
PPI: Protein-protein interaction
*rlog*: Regularize-logarithm transformed function
TCA: Tricarboxylic acid cycle or Krebs cycle
TF: Transcription factor
TRN: transcriptional regulatory network

## Footnotes

1
http://www.computationalbiology.ufpa.br/gofeat/

2
https://string-db.org/
