# Supplementary material for: Transcriptome profile of Corynebacterium pseudotuberculosis in response to iron limitation
Source: BMC Genomics. 2019 Aug 20;20:663. doi: 10.1186/s12864-019-6018-1 (PMC6701010; doi:10.1186/s12864-019-6018-1)
Supplement: Supplementary file 1 — Table S1. Sequencing statistics. Table S2. Total number of processed reads from High Iron (HI) and Low Iron (LI) experimental condition. Table S3. Mapping results. Table S4. Raw gene counts. Figure S1. Sample to sample principal component analysis (PCA) (DOCX 129 kb) [file 12864_2019_6018_MOESM1_ESM.docx]

Additional file 1

**Transcriptome Profile of *Corynebacterium pseudotuberculosis* in Response to Iron Limitation**

Izabela Coimbra Ibraim^1^, Mariana Teixeira Dornelles Parise^1^, Doglas Parise^1^, Michelle Zibetti Tadra Sfeir^2^, Thiago Luiz de Paula Castro^3^, Alice Rebecca Wattan^4^, Preetam Ghosh^5^, Debmalya Barh^1^, Emannuel Maltempi Souza^2^, Aristóteles Góes-Neto^6^, Anne Cybelle Pinto Gomide^a1^, Vasco Azevedo^a1*^

Corresponding Author: Dr. Vasco Azevedo - vasco@icb.ufmg.br

Table of Contents:

Table S1. Sequencing statistics

Table S2. Total number of processed reads from High Iron (HI) and Low Iron (LI) experimental condition

Table S3. Mapping results

Table S4. Raw gene counts

Figure S1. Sample to sample principal component analysis (PCA) and hierarchical clustering analysis

Table S1. Sequencing statistics. Paired samples from High Iron (HI) and Low Iron (LI) experimental condition in the wild-type T1 and Cp13 strains were sequenced in two Ion proton runs (run 1 and run 2), yielding over 120 million single-end reads (124.251.275 total). Overall read length vary from 8 to 380 bp with an average CG content of 53-55%. The pair sample (S.3) from the wild-type T1 strain was eliminated from further analysis due to the low sequenced read count.

| Strain | Sample ID | Condition | Average read length (bp) | Average CG content (%) | Number of reads | Batch Id | Run 1 | Run 2 |
| --- | --- | --- | --- | --- | --- | --- | --- | --- |
| Cp13 mutant | S.1 | HI | 8-369 | 53 | 6,014,333 | A | ✓ |  |
|  | S.1 | LI | 8-375 | 53 | 10,905,772 | A | ✓ |  |
|  | S.2 | HI_1 | 8-368 | 55 | 3,936,728 | B | ✓ |  |
|  |  | HI_2 | 8-366 | 55 | 9,035,553 | B |  | ✓ |
|  | S.2 | LI | 8-368 | 54 | 4,834,840 | C | ✓ |  |
|  | S.3 | HI | 8-374 | 55 | 8,974,704 | C | ✓ |  |
|  | S.3 | LI | 8-378 | 55 | 4,236,781 | C | ✓ |  |
|  | S.4 | HI_1 | 8-368 | 54 | 1,789,770 | C | ✓ |  |
|  |  | HI_2 | 8-369 | 54 | 3,327,609 | C |  | ✓ |
|  | S.4 | LI_1 | 8-369 | 55 | 2,792,977 | C | ✓ |  |
|  |  | LI_2 | 8-364 | 55 | 6,038,287 | C |  | ✓ |
| Wild-type T1 | S.1 | HI_1 | 8-378 | 55 | 3,988,317 | C | ✓ |  |
|  |  | HI_2 | 8-368 | 55 | 7,886,336 | C |  | ✓ |
|  | S.1 | LI_1 | 8-355 | 55 | 3,537,940 | C | ✓ |  |
|  |  | LI_2 | 8-366 | 55 | 7,801,599 | C |  | ✓ |
|  | S.2 | HI | 8-370 | 55 | 11,975,500 | C | ✓ |  |
|  | S.2 | LI | 8-369 | 55 | 14,575,386 | C | ✓ |  |
|  | S.3 | HI | 8-380 | 54 | 38,385 | C | ✓ |  |
|  | S.3 | LI | 8-345 | 54 | 12,560,458 | C |  | ✓ |

| Strain | Sample Identification | Condition | Number of reads | Number of trimmed/processed reads | % of excluded reads | Phred score |
| --- | --- | --- | --- | --- | --- | --- |
| **Cp13 mutant** | S.1 | HI | 6,014,33 | 6,012,724 (99.9%) | 1,609 (0.03%) | 24 |
|  | S.1 | LI | 10,905,772 | 10,902,511 (99.97%) | 3,261 (0.03%) | 24 |
|  | S.2 | HI | 3,936,728 | 3,931,106 (99.86%) | 5,622 (0.14%) | 25 |
|  |  |  | 9,035,553 | 9,018,421 (99.81%) | 17,132 (0,19%) | 25 |
|  | S.2 | LI | 4,834,840 | 4,830,590 (99.91%) | 4,250 (0.09%) | 25 |
|  | S.3 | HI | 8,974,704 | 8.964,375 (99.88%) | 10,329 (0.12%) | 25 |
|  | S.3 | LI | 4,236,781 | 4,233,505 (99.92%) | 3.276 (0.08%) | 24 |
|  | S.4 | HI | 1,789,770 | 1,788,363 (99.92%) | 1,407 (0.08%) | 25 |
|  |  |  | 3,327,609 | 3,324,827 (99.92%) | 2,782 (0.08%) | 24 |
|  | S.4 | LI | 2,792,977 | 2,791,946 (99.96%) | 1,031 (0,04%) | 25 |
|  |  |  | 6,038,287 | 6,035.682 (99.96%) | 2,605 (0.04%) | 25 |
| **Wild-type T1** | S.1 | HI | 3,988,317 | 3,985,364 (99.93%) | 2,953 (0.07%) | 25 |
|  |  |  | 7,886,336 | 7,878,693 (99.90%) | 7,643 (0.10%) | 25 |
|  | S.1 | LI | 3,537,940 | 3,535,701 (99.94%) | 2,239 (0.06%) | 25 |
|  |  |  | 7,801,599 | 7,795,681 (99.92%) | 5,918 (0,08%) | 25 |
|  | S.2 | HI | 11,975,500 | 11,967,330 (99.93%) | 8,170 (0,07%) | 25 |
|  | S.2 | LI | 14,575,386 | 14,561,588 (99.91%) | 13,798 (0,09%) | 24 |

Table S2. Total number of processed reads from High iron (HI) and Low Iron (LI) experimental condition in the wild-type T1 and Cp13 mutant strains. Total number of reads, number of processed reads and percentage of excluded reads after applying a quality filtering score of 10 (Phred score 10) over the average base quality of the sliding window using Trimmomatic. Trimmed reads were quality checked using the FASTQC and presented an average read quality score of 24-25.

Table S3. Mapping results. Paired samples from High Iron (HI) and Low Iron (LI) experimental condition in the wild-type T1 and Cp13 mutant strains. The total number of reads and percentage of total single-end reads mapped to the *Corynebacterium pseudotuberculosis* strain 1002B genome (NZ_CP012837.1), using the Torrent Mapping Alignment Program (TMAP).

| Strain | Sample Identification | Condition | Number of reads | Number of unmapped reads | Number of mapped reads | % of mapped reads |
| --- | --- | --- | --- | --- | --- | --- |
| ciuA mutant Cp13 | S.1 | HI | 6,012,724 | 77,775 | 5,934,949 | 98.7 |
|  | S.1 | LI | 10,902,511 | 117,242 | 10,785,269 | 98.9 |
|  | S.2 | HI | 12,949,527 | 3,232,235 | 9,717,292 | 75.0 |
|  | S.2 | LI | 4,830,590 | 1,325,346 | 3,505,244 | 72.6 |
|  | S.3 | HI | 8,964,375 | 1,776,069 | 7,188,306 | 80.2 |
|  | S.3 | LI | 4,233,505 | 672,232 | 3,561,273 | 84.1 |
|  | S.4 | HI | 5,113,190 | 935,598 | 4,177,592 | 81.7 |
|  | S.4 | LI | 8,827,628 | 2,348,088 | 6,479,540 | 73.4 |
| Wild-type T1 | S.1 | HI | 11,864,057 | 1,793,682 | 10,070,375 | 84.9 |
|  | S.1 | LI | 11,331,382 | 3,024,330 | 8,307,052 | 73.3 |
|  | S.2 | HI | 11,967,330 | 2,342,791 | 9,624,539 | 80.4 |
|  | S.2 | LI | 14,561,588 | 2,664,406 | 11,897,182 | 81.7 |

Table S4. Number of unique and ambiguous (multiple) alignments. Paired samples from High Iron (HI) and Low Iron (LI) experimental condition in the wild-type T1 and Cp13 mutant strains with the total number of read counts aligned to unique features using HTSeq-count are presented in raw counted data and % of unique aligned reads. Reads that were assigned to more than one feature were counted as ambiguous and were not counted for any features (--nonunique none).

| Strain | Sample Identification | Exp. Condition | Number of mapped reads | Ambiguous alignments | Not aligned | Unique alignments | % of unique alignments |
| --- | --- | --- | --- | --- | --- | --- | --- |
| ciuA mutant Cp13 | S.1 | HI | 5,934,949 | 4,231,060 | 77,775 | 1,703,889 | 28.71 |
|  | S.1 | LI | 10,785,269 | 7,637,579 | 117,242 | 3,147,690 | 29.19 |
|  | S.2 | HI | 9,717,292 | 8,749,308 | 3,232,235 | 3,266,366 | 27.18 |
|  | S.2 | LI | 3,505,244 | 2,508,091 | 1,325,346 | 997,153 | 28.45 |
|  | S.3 | HI | 7,188,306 | 5,152,800 | 1,776,069 | 2,035,506 | 28.32 |
|  | S.3 | LI | 3,561,273 | 2,570,497 | 672,232 | 990,776 | 27.82 |
|  | S.4 | HI | 4,177,592 | 3,448,057 | 935,598 | 1,347,008 | 28.09 |
|  | S.4 | LI | 6,479,540 | 5,763,821 | 2,348,088 | 2,340,924 | 28.88 |
| Wild-type T1 | S.1 | HI | 10,070,375 | 8,060,346 | 1,793,682 | 3,232,915 | 28.63 |
|  | S.1 | LI | 8,307,052 | 7,426,007 | 3,024,330 | 2,994,533 | 28.74 |
|  | S.2 | HI | 9,624,539 | 6,900,236 | 2,342,791 | 2,724,303 | 28.31 |
|  | S.2 | LI | 11,897,182 | 8,622,027 | 2,664,406 | 3,275,155 | 27.53 |


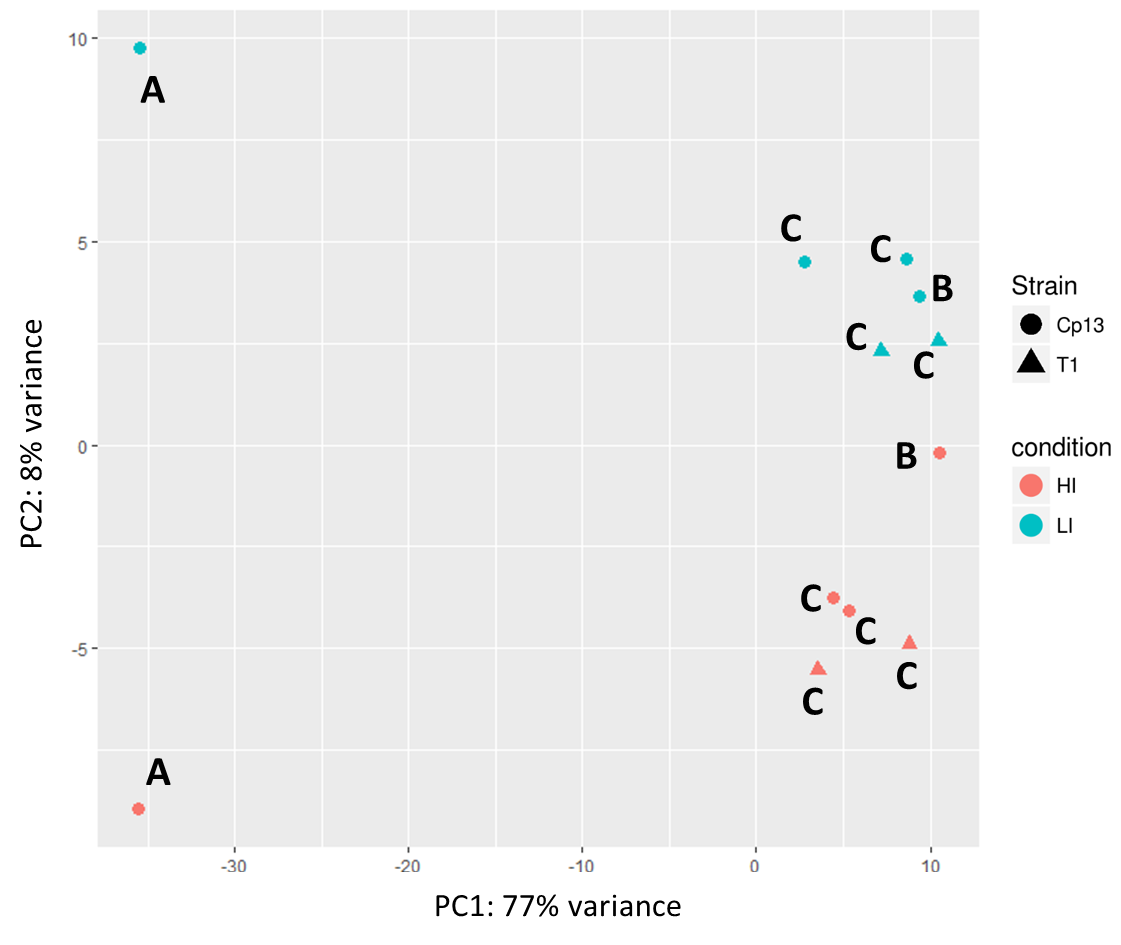


Figure S1. Sample to sample principal component analysis (PCA) and hierarchical clustering analyses. PCA plot for the biological replicates of the T1 wild-type and Cp13 mutant strains. PCA and heatmap were plotted with *rlog*-transformed data of normalized read counts. The first two principal components are shown on the X and Y axes, with the proportion of total variance attributed to that PC indicated. Each experimental sample is represented as a single point with color indicating experimental sample condition and shape indicating strain. Pink represents High Iron (HI) and blue Low Iron (LI) experimental samples. Strains were represented by geometrical figures: T1 wild-type strain (▲) and Cp13 mutant strain (●). Batches are labeled A-C. PC1 evidencing batch clustering and sample variation within batch A and batches B-C of the replicates. PC1 and PC2 represent 85% of the total variance of the samples. HI and LI grouping can be seen, representing 8% of the total variance (PC2). Batch effect was attribute to sample preparation and it was included into the DESeq2 design formula.
