# Supplementary material for: Transcriptome profile of Corynebacterium pseudotuberculosis in response to iron limitation
Source: BMC Genomics. 2019 Aug 20;20:663. doi: 10.1186/s12864-019-6018-1 (PMC6701010; doi:10.1186/s12864-019-6018-1)
Supplement: Supplementary file 2 — Figure S2. Effect of iron depletion on the growth curve and growth rate of C. pseudotuberculosis wild-type T1 strain and Cp13 mutant. Figure S3. Effects of ethanol on bacterial growth (DOCX 261 kb) [file 12864_2019_6018_MOESM2_ESM.docx]

Additional file 2

**Transcriptome Profile of *Corynebacterium pseudotuberculosis* in Response to Iron Limitation**

Izabela Coimbra Ibraim^1^, Mariana Teixeira Dornelles Parise^1^, Doglas Parise^1^, Michelle Zibetti Tadra Sfeir^2^, Thiago Luiz de Paula Castro^3^, Alice Rebecca Wattan^4^, Preetam Ghosh^5^, Debmalya Barh^1^, Emannuel Maltempi Souza^2^, Aristóteles Góes-Neto^6^, Anne Cybelle Pinto Gomide^a1^, Vasco Azevedo^a1*^

Corresponding Author: Dr. Vasco Azevedo - vasco@icb.ufmg.br

Table of Contents:

Figure S2. Effect of iron depletion on the growth curve and growth rate of *C. pseudotuberculosis* wild-type T1 strain and Cp13 mutant

Figure S3. Effects of ethanol on bacterial growth


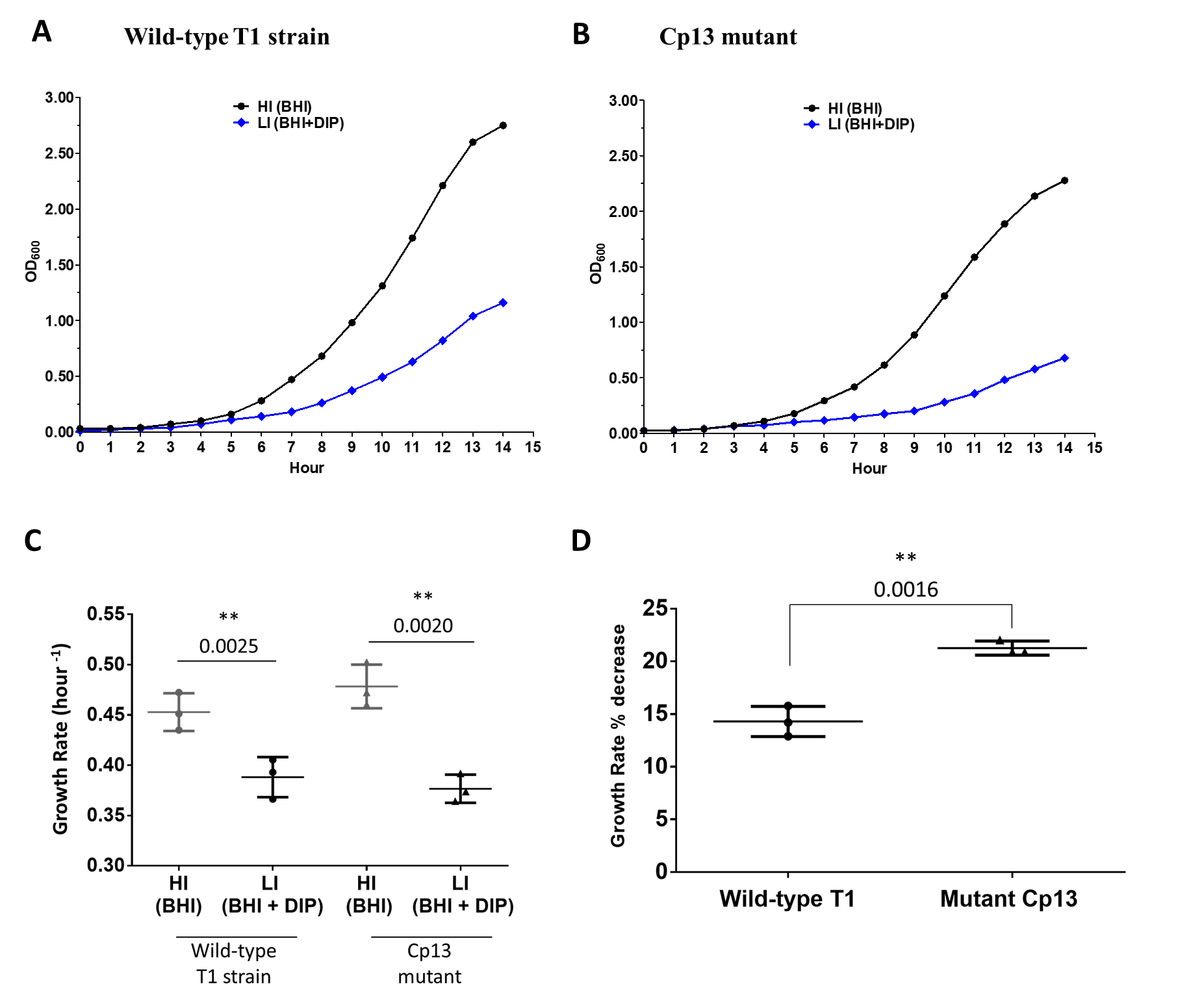


Figure S2. Effect of iron depletion on the growth curve and growth rate of *C. pseudotuberculosis* wild-type T1 strain and Cp13 mutant. Cultures of the (**A**) wild-type T1 strain and the (**B**) Cp13 mutant were cultivated at 37°C in BHI broth (HI – high iron) and BHI broth treated with 250 µM 2,2-dipyridyl (DIP) (LI -low iron). Growth proliferation was measured hourly by optical density for 14 hours at 600nm. For ease of interpretation, data is plotted on a linear scale. (**C**) Log rate plots of OD 600 for the wild-type T1 and Cp13 mutant were calculated using the growth rate equation: $Growth rate=(2.303*\left( \log_{10} ODf \right)- (\log_{10} ODi))/(T_{final}- T_{initial})$, where OD*_i_* represents the optical density (OD_600_) at the start of the incubation period (t=0) and OD*_f_* is the OD_600_ value at the final incubation time (t= 6h30) in BHI medium, with and without 250 µM 2,2-dipyridyl (DIP). Horizontal lines indicate the mean of 3 individual assays and vertical lines standard deviation (sd) from mean. Statistical analysis using a paired t-test confirmed that iron limitation induced a significant effect on the growth rate of the Cp13 (*p* value = 0.0020) and T1 strains (*p* value = 0.0025). No difference was observed when analyzing HI and LI samples between the strains (student t-test, *p* value > 0.05). (**D**) Limited iron availability induced an average decrease of 21.3% in the growth rate of the Cp13 mutant and 14.3% mean average decrease in the wild-type T1 strain.


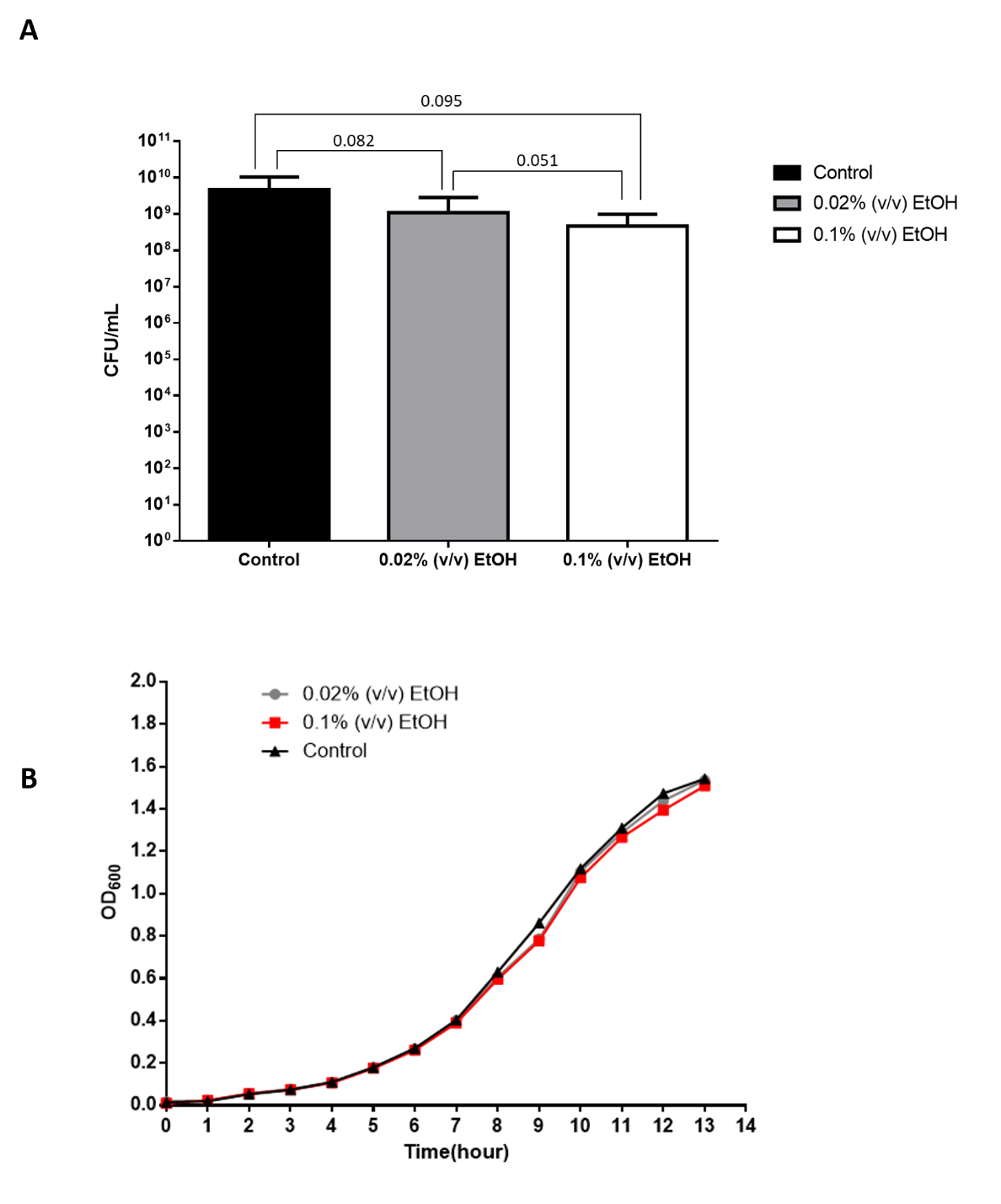


Figure S3. Effects of ethanol on bacterial growth. (**A**) The effects of ethanol on the growth of the *C. pseudotuberculosis* Cp13 strain was measured by determining the number of CFU/mL after 13 hours of incubation in BHI control medium and BHI media supplemented with 0,02% and 0,1% of ethanol (v/v). No significant difference was observed between the CFU counts of control and ethanol supplemented cultures (p > 0.05). (**B)** growth proliferation was measured hourly by optical density for 13 hours at 600nm in control, 0.02% and 0.01% of ethanol cultures.
