# Supplementary material for: Transcriptome profile of Corynebacterium pseudotuberculosis in response to iron limitation
Source: BMC Genomics. 2019 Aug 20;20:663. doi: 10.1186/s12864-019-6018-1 (PMC6701010; doi:10.1186/s12864-019-6018-1)
Supplement: Supplementary file 3 — Table S5. Differentially expressed genes identified in the T1 strain in response to iron limitation. Table S6. Differentially expressed genes identified in the Cp13 mutant in response to iron limitation (DOCX 63 kb) [file 12864_2019_6018_MOESM3_ESM.docx]

Additional file 3

**Transcriptome Profile of *Corynebacterium pseudotuberculosis* in Response to Iron Limitation**

Izabela Coimbra Ibraim^1^, Mariana Teixeira Dornelles Parise^1^, Doglas Parise^1^, Michelle Zibetti Tadra Sfeir^2^, Thiago Luiz de Paula Castro^3^, Alice Rebecca Wattan^4^, Preetam Ghosh^5^, Debmalya Barh^1^, Emannuel Maltempi Souza^2^, Aristóteles Góes-Neto^6^, Anne Cybelle Pinto Gomide^a1^, Vasco Azevedo^a1*^

Corresponding Author: Dr. Vasco Azevedo - vasco@icb.ufmg.br

Table of Contents:

Table S5. Differentially expressed genes identified in the T1 strain in response to iron limitation

Table S6. Differentially expressed genes identified in the Cp13 mutant in response to iron limitation

Table S5. Differentially expressed genes identified in the T1 strain in response to iron limitation

| Refseq_protein | Gene/Locus ID | Product | LI:Ct Fold Change | LI:Ct Log2FoldChange | FDR | Gene reg. |
| --- | --- | --- | --- | --- | --- | --- |
| WP_013240877.1 | *Cp1002B_95* | Hypothetical protein | 1.70 | -0.761910 | 2.14719E-05 | DOWN |
| WP_013240878.1 | *ppiA* | Peptidyl-prolyl cis-trans isomerase | 2.07 | -1.049914 | 1.85484E-06 | DOWN |
| WP_013240933.1 | *Cp1002B_350* | Transcription factor rok | 1.54 | -0.620500 | 1.32554E-07 | DOWN |
| WP_013240983.1 | *sdcS* | Sodium-dependent dicarboxylate transporter | 2.35 | -1.231800 | 2.83533E-18 | DOWN |
| WP_013241065.1 | *glxR* | Crp/Fnr family transcriptional regulator | 1.59 | 0.668724 | 0.00295783 | UP |
| WP_013241099.1 | *lpd* | Dihydrolipoamide dehydrogenase | 1.77 | -0.821935 | 3.89617E-05 | DOWN |
| WP_013241101.1 | *sdhC* | Succinate dehydrogenase cytochrome b556 subunit | 1.57 | -0.649583 | 1.34394E-06 | DOWN |
| WP_013241102.1 | *sdhA* | Succinate dehydrogenase flavoprotein subunit | 1.51 | -0.590974 | 0.000102809 | DOWN |
| WP_013241103.1 | *sdhB* | Succinate dehydrogenase iron-sulfur subunit | 1.86 | -0.897827 | 6.39657E-15 | DOWN |
| WP_013241104.1 | *Cp1002B_1235* | Hypothetical protein | 2.17 | -1.120063 | 8.79442E-05 | DOWN |
| WP_013241113.1 | *Cp1002B_1280* | Hypothetical protein | 1.36 | -0.444455 | 0.002247494 | REG |
| WP_013241147.1 | *ccsA* | Cytochrome c biogenesis protein ccsa | 1.53 | 0.617462 | 0.001022724 | UP |
| [WP_013241165.1](http://www.ncbi.nlm.nih.gov/protein/WP_013241165.1) | *rplA* | 50S ribosomal protein L1 | 1.32 | -0.403942 | 0.000666323 | REG |
| WP_014300452.1 | *rplJ* | 50s ribosomal protein l10 | 1.74 | -0.796356 | 0.000812793 | DOWN |
| WP_013241171.1 | *rplL* | 50s ribosomal protein l7/l12 | 1.80 | -0.845724 | 0.000186583 | DOWN |
| WP_013241172.1 | *Cp1002B_1590* | Hypothetical protein | 1.37 | -0.452040 | 0.000305149 | REG |
| WP_013241174.1 | *Cp1002B_1600* | Hypothetical protein | 1.49 | -0.577251 | 2.7149E-06 | REG |
| [WP_013241177.1](http://www.ncbi.nlm.nih.gov/protein/WP_013241177.1) | *Cp1002B_1615* | hypothetical protein | 1.49 | -0.570888 | 0.001660433 | REG |
| WP_013241191.1 | *rpsL* | 30s ribosomal protein s12 | 1.86 | -0.896705 | 3.80001E-06 | DOWN |
| WP_013241193.1 | *fusA* | Elongation factor g (ef-g) | 1.62 | -0.695013 | 0.001030848 | DOWN |
| WP_013241203.1 | *rplC* | 50s ribosomal protein l3 | 1.47 | -0.553653 | 0.000169055 | REG |
| WP_013241209.1 | *rpsC* | 30s ribosomal protein s3 | 1.50 | -0.589148 | 2.82806E-06 | DOWN |
| [WP_013910826.1](http://www.ncbi.nlm.nih.gov/protein/WP_013910826.1) | *rpsM* | 30S ribosomal protein S13 | 1.63 | -0.705070 | 0.003009419 | DOWN |
| [WP_013241260.1](http://www.ncbi.nlm.nih.gov/protein/WP_013241260.1) | *rplM* | 50S ribosomal protein L13 | 1.65 | -0.719634 | 0.002412353 | DOWN |
| WP_013241261.1 | *rpsI* | 30s ribosomal protein s9 | 1.97 | -0.979783 | 2.95922E-07 | DOWN |
| WP_013241263.1 | *Cp1002B_2040* | Hypothetical protein | 1.72 | 0.783698 | 0.001156371 | UP |
| [WP_013241266.1](http://www.ncbi.nlm.nih.gov/protein/WP_013241266.1) | *Cp1002B_2050* | hypothetical protein | 1.67 | 0.741298 | 0.001724138 | UP |
| WP_004566891.1 | *groES* | Co-chaperone groes | 1.70 | -0.768056 | 7.83744E-06 | DOWN |
| [WP_013241276.1](http://www.ncbi.nlm.nih.gov/protein/WP_013241276.1) | *groEL* | molecular chaperone GroEL | 1.86 | -0.897579 | 0.001764785 | DOWN |
| WP_013241310.1 | *htaA* | Cell-surface hemin receptor | 1.95 | 0.962387 | 2.56552E-14 | UP |
| WP_013241311.1 | *hmuT* | Hemin-binding periplasmic protein hmut | 1.86 | 0.892215 | 1.20551E-13 | UP |
| WP_013241312.1 | *hmuU* | Hemin import atp-binding protein | 1.50 | 0.584874 | 6.21045E-07 | UP |
| WP_013241314.1 | *htaC* | Membrane protein | 6.93 | 2.793618 | 4.97068E-69 | UP |
| Cp1002B_2285 | *Cp1002B_2285* | O-acetyl-L-homoserine sulfhydrolase | 1.69 | 0.7589 | 1.74E-07 | UP |
| WP_013241337.1 | *accBC* | Acyl coenzyme a carboxylase | 1.47 | -0.551264 | 9.14909E-05 | REG |
| WP_013241338.1 | *cysA* | Sulfurtransferase | 1.39 | -0.472489 | 5.40782E-05 | REG |
| WP_013241378.1 | *Cp1002B_2580* | Hypothetical protein | 1.50 | -0.585806 | 0.000132477 | DOWN |
| WP_014522151.1 | *fecB* | Periplasmic binding protein | 2.03 | 1.023079 | 1.84161E-10 | UP |
| WP_013242872.1 | *Cp1002B_2935* | Transglycosylase associated protein | 2.47 | -1.303432 | 3.28002E-17 | DOWN |
| [WP_013242865.1](http://www.ncbi.nlm.nih.gov/protein/WP_013242865.1) | *hrrS* | two-component system sensor histidine kinase | 1.33 | 0.414158 | 0.003040232 | REG |
| WP_013242864.1 | *hrrA* | Two-component system transcriptional regulatory protein | 1.68 | 0.747336 | 4.28669E-08 | UP |
| WP_013242852.1 | *sodA* | Manganese superoxide dismutase | 1.94 | -0.958009 | 5.64712E-16 | DOWN |
| WP_014522292.1 | *Cp1002B_3070* | Hypothetical protein | 8.49 | 3.086410 | 3.5298E-37 | UP |
| WP_013242841.1 | *Cp1002B_3075* | Hypothetical protein | 10.19 | 3.348728 | 2.60248E-50 | UP |
| WP_014401453.1 | *lytR* | Lytr family transcriptional regulator | 1.47 | 0.553600 | 6.56646E-05 | REG |
| [WP_013242822.1](http://www.ncbi.nlm.nih.gov/protein/WP_013242822.1) | *glpQ* | glycerophosphoryl diester phosphodiesterase | 1.38 | -0.466029 | 0.000206749 | REG |
| WP_013242563.1 | *Cp1002B_4430* | Hypothetical protein | 1.56 | 0.637332 | 4.6245E-07 | UP |
| WP_013242548.1 | *Cp1002B_4545* | Abc transporter atp-binding protein | 1.36 | -0.447958 | 0.001367634 | REG |
| WP_013242547.1 | *Cp1002B_4550* | Abc-type antimicrobial peptide transport system | 1.55 | -0.630213 | 0.000120287 | DOWN |
| WP_014367533.1 | *ftn* | Ferritin-like protein | 2.36 | -1.237090 | 7.85923E-15 | DOWN |
| WP_013242523.1 | *ctaD* | Cytochrome c oxidase subunit 1 | 1.93 | -0.947923 | 1.54564E-07 | DOWN |
| WP_013242515.1 | *Cp1002B_4725* | Rhomboid family protein | 1.66 | -0.730178 | 4.04547E-05 | DOWN |
| [WP_013242461.1](http://www.ncbi.nlm.nih.gov/protein/WP_013242461.1) | *tig* | trigger factor | 1.44 | -0.525525 | 0.000524198 | REG |
| WP_013242460.1 | *clpP* | Atp-dependent clp protease proteolytic subunit | 1.42 | -0.507650 | 0.000343248 | REG |
| [WP_013242446.1](http://www.ncbi.nlm.nih.gov/protein/WP_013242446.1) | *rpmA* | 50S ribosomal protein L27 | 1.51 | -0.595384 | 0.001681922 | DOWN |
| [WP_014522238.1](http://www.ncbi.nlm.nih.gov/protein/WP_014522238.1) | *hmuO* | heme oxygenase | 1.47 | 0.556247 | 0.00175222 | REG |
| WP_013242332.1 | *htaF* | Cell-surface hemin receptor | 3.38 | 1.755780 | 4.15589E-27 | UP |
| WP_013242331.1 | *htaG* | Uncharacterized protein htac | 2.78 | 1.473360 | 1.10176E-26 | UP |
| WP_046341090.1 | *aceF* | Dihydrolipoamide acyltransferase | 2.25 | -1.172387 | 8.6232E-09 | DOWN |
| [WP_013242285.1](http://www.ncbi.nlm.nih.gov/protein/WP_013242285.1) | *Cp1002B_5875* | hypothetical protein | 1.42 | -0.505116 | 0.002865682 | REG |
| WP_013242283.1 | *ctaC* | Cytochrome c oxidase subunit ii | 2.48 | -1.307544 | 4.1942E-15 | DOWN |
| WP_013242282.1 | *ctaF* | Cytochrome aa3 subunit 4 | 2.23 | -1.157272 | 8.87325E-13 | DOWN |
| WP_013242281.1 | *ctaE* | Cytochrome c oxidase subunit iii | 2.31 | -1.205747 | 1.10736E-11 | DOWN |
| WP_013242280.1 | *qcrC* | Ubiquinol-cytochrome c reductase cytochrome c subunit | 2.84 | -1.508014 | 6.66346E-17 | DOWN |
| WP_014300818.1 | *qcrA* | Ubiquinol-cytochrome c reductase iron-sulfur subunit | 2.35 | -1.235498 | 1.50312E-06 | DOWN |
| WP_013242278.1 | *qcrB* | Ubiquinol-cytochrome c reductase cytochrome b subunit | 2.42 | -1.277399 | 3.57326E-15 | DOWN |
| WP_013242277.1 | *Cp1002B_5915* | Hypothetical protein, npl/p60 family rote | 1.43 | 0.511751 | 0.000266284 | REG |
| WP_013242261.1 | *mraZ* | Transcriptional regulator mraz | 1.95 | -0.964893 | 3.28102E-07 | DOWN |
| WP_013242245.1 | *ag84* | Antigen 84 | 1.68 | -0.744234 | 2.59512E-06 | DOWN |
| [WP_013242172.1](http://www.ncbi.nlm.nih.gov/protein/WP_013242172.1) | *xerC* | DNA processing protein DprA | 1.30 | 0.383920 | 0.001755934 | REG |
| WP_013242168.1 | *tsf* | Elongation factor ts (ef-ts) | 1.76 | -0.819061 | 0.000609445 | DOWN |
| WP_038616474.1 | *mapB* | methionine aminopeptidase | 1.40 | 0.485872 | 0.00149511 | REG |
| [WP_013242137.1](http://www.ncbi.nlm.nih.gov/protein/WP_013242137.1) | *ylxR* | DNA-binding protein | 1.35 | -0.432620 | 0.001920312 | REG |
| WP_013242135.1 | *rbfA* | Ribosome-binding factor a | 1.62 | -0.696887 | 5.74792E-05 | DOWN |
| WP_013242094.1 | *ptsH* | Phosphocarrier protein hpr | 1.48 | -0.568824 | 0.00131324 | REG |
| [WP_013242073.1](http://www.ncbi.nlm.nih.gov/protein/WP_013242073.1) | *sigB* | RNA polymerase sigma factor SigB | 1.37 | -0.457111 | 0.001568058 | REG |
| [WP_013242016.1](http://www.ncbi.nlm.nih.gov/protein/WP_013242016.1) | *citE* | citrate lyase subunit beta | 1.43 | 0.513988 | 0.001650089 | REG |
| WP_013241994.1 | *nusB* | Transcription antitermination protein nusb | 1.44 | -0.526362 | 0.000136463 | REG |
| [WP_013241984.1](http://www.ncbi.nlm.nih.gov/protein/WP_013241984.1) | *Cp1002B_7360* | hypothetical protein | 1.48 | -0.569637 | 0.002657504 | REG |
| WP_013241796.1 | *doxX* | Doxx family protein | 1.39 | -0.472414 | 5.25994E-05 | REG |
| [WP_057061524.1](http://www.ncbi.nlm.nih.gov/protein/WP_057061524.1) | *Cp1002B_8515* | hypothetical protein | 1.33 | 0.407664 | 0.002131544 | REG |
| WP_014522179.1 | *atpH* | F-type atpase subunit delta | 1.58 | -0.657731 | 0.000150468 | DOWN |
| WP_013241701.1 | *atpF* | F-type atpase subunit b | 1.57 | -0.654918 | 2.11641E-05 | DOWN |
| WP_013241696.1 | *ywlC* | Threonylcarbamoyl-amp synthase | 1.60 | 0.678432 | 0.000725474 | UP |
| WP_013241684.1 | *lutB* | Lactate utilization protein b | 2.11 | -1.075741 | 3.23659E-15 | DOWN |
| WP_013241683.1 | *Cp1002B_8845* | Hypothetical protein | 1.52 | -0.605213 | 3.72574E-06 | DOWN |
| [WP_013241666.1](http://www.ncbi.nlm.nih.gov/protein/WP_013241666.1) | *galK* | galactokinase | 1.29 | 0.366387 | 0.00275072 | REG |
| WP_013241654.1 | *odhA* | Alpha-ketoglutarate decarboxylase | 1.45 | -0.540864 | 0.002471747 | REG |
| WP_013241593.1 | *fumC* | Fumarate hydratase class ii (fumarase c) (ec 4.2.1.2) (aerobic fumarase) (iron-independent fumarase) | 1.43 | -0.511878 | 0.000272047 | REG |
| [WP_013241591.1](http://www.ncbi.nlm.nih.gov/protein/WP_013241591.1) | *Cp1002B_9300* | MFS transporter | 1.32 | 0.401988 | 0.000330379 | REG |
| WP_039697516.1 | *ripA* | Arac family transcriptional regulator-hth-type transcriptional repressor of iron protein A | 2.13 | 1.091910 | 1.19311E-12 | UP |
| [WP_013241535.1](http://www.ncbi.nlm.nih.gov/protein/WP_013241535.1) | *rpfB* | resuscitation-promoting factor | 1.32 | 0.396790 | 0.002133833 | REG |
| WP_013241520.1 | *mscL* | Large conductance mechanosensitive channel protein mscl | 1.49 | -0.578272 | 0.000228706 | REG |
| [WP_013241517.1](http://www.ncbi.nlm.nih.gov/protein/WP_013241517.1) | *tcsS4* | two-component system sensor histidine kinase | 1.45 | -0.532569 | 0.000333247 | REG |
| WP_013241513.1 | *rpmB* | 50s ribosomal protein l28 | 1.42 | -0.509815 | 8.2349E-05 | REG |
| [WP_013241512.1](http://www.ncbi.nlm.nih.gov/protein/WP_013241512.1) | *rpsN* | 30S ribosomal protein S14 | 1.44 | -0.524718 | 0.001890195 | REG |
| [WP_013241487.1](http://www.ncbi.nlm.nih.gov/protein/WP_013241487.1) | *cobF* | precorrin 6A synthase | 1.35 | -0.436293 | 0.001649632 | REG |
| [WP_013241480.1](http://www.ncbi.nlm.nih.gov/protein/WP_013241480.1) | *arcB* | ornithine cyclodeaminase | 1.47 | 0.557814 | 5.48628E-06 | REG |
| [WP_014300556.1](http://www.ncbi.nlm.nih.gov/protein/WP_014300556.1) | *Cp1002B_9880* | sodium:proton antiporter | 1.39 | 0.475246 | 0.002782863 | REG |
| WP_013241465.1 | *fkbP* | Peptidyl-prolyl cis-trans isomerase | 1.97 | -0.980577 | 5.7635E-06 | DOWN |
| WP_013241451.1 | *rpfA* | Resuscitation-promoting factor | 1.59 | -0.665839 | 3.4754E-05 | DOWN |
| WP_013242913.1 | *Cp1002B_10210* | Hypothetical protein | 5.43 | -2.441716 | 1.17496E-19 | DOWN |
| WP_013242924.1 | *Cp1002B_10265* | Hypothetical protein | 1.78 | -0.827959 | 1.03231E-07 | DOWN |
| WP_013240910.1 | *Cp1002B_10495* | Hypothetical protein | 4.97 | 2.313972 | 3.1878E-65 | UP |
| WP_003402602.1 | *Cp1002B_10540* | AURKAIP1/COX24 domain-containing protein | 1.84 | -0.881351 | 0.002694085 | DOWN |
| WP_014300599.1 | *Cp1002B_10775* | Alkylmercury lyase | 1.43 | -0.515499 | 0.000454602 | REG |
| WP_014300577.1 | *Cp1002B_10785* | Hypothetical protein | 3.59 | 1.843259 | 9.35463E-20 | UP |
|  | *Cp1002B_190* | tRNA | 2.52 | -1.3338 | 3.85011E-16 | DOWN |
|  | *Cp1002B_730* | tRNA | 2.36 | -1.2389 | 0.002888947 | DOWN |
|  | *Cp1002B_3725* | tRNA | 1.34 | 0.4215 | 0.000165761 | REG |
|  | *Cp1002B_4230* | tRNA | 5.60 | -2.4859 | 6.73591E-13 | DOWN |
|  | *Cp1002B_4475* | tRNA | 1.85 | -0.8846 | 1.59602E-11 | DOWN |
|  | *Cp1002B_4760* | tRNA | 1.74 | -0.7992 | 0.003038351 | DOWN |
|  | *Cp1002B_4840* | tRNA | 1.65 | -0.7222 | 6.03451E-06 | DOWN |
|  | *Cp1002B_4880* | tRNA | 2.01 | -1.0093 | 5.66402E-05 | DOWN |
|  | *Cp1002B_5535* | tRNA | 1.45 | -0.5404 | 0.000101199 | REG |
|  | *Cp1002B_7030* | tRNA | 1.66 | -0.7282 | 0.000330746 | DOWN |
|  | *Cp1002B_7045* | tRNA | 1.64 | -0.7174 | 1.90589E-05 | DOWN |
|  | *Cp1002B_8015* | tRNA | 1.29 | -0.3711 | 0.002527029 | REG |
|  | *Cp1002B_9440* | tRNA | 1.73 | -0.7907 | 9.59216E-08 | DOWN |

Table S6. Differentially expressed genes identified in the Cp13 mutant strain in response to iron limitation

| Refseq_protein | Gene/Locus ID | Product | LI:Ct Fold Change | LI:Ct Log2FoldChange | FDR | Gene reg. |
| --- | --- | --- | --- | --- | --- | --- |
| WP_014300357.1 | *Cp1002B_70* | Hypothetical protein | 2.12 | 1.08137012 | 1.69E-06 | UP |
| [WP_013240877.1](http://www.ncbi.nlm.nih.gov/protein/WP_013240877.1) | *Cp1002B_95* | Hypothetical protein | 1.45 | -0.53247980 | 0.019094153 | REG |
| WP_013240983.1 | *sdcS* | Sodium-dependent dicarboxylate transporter | 1.47 | -0.56035596 | 0.011372589 | REG |
| WP_013240987.1 | *Cp1002B_640* | Glyoxalase/Bleomycin resistance protein/Dihydroxybiphenyl dioxygenase | 1.36 | -0.44523800 | 0.014083724 | REG |
| WP_013241021.1 | *deoR* | Deor family transcriptional regulator | 1.33 | -0.40698491 | 0.025766871 | REG |
| WP_013241084.1 | *cspA* | Cold-shock protein | 1.86 | 0.89665067 | 0.000775566 | UP |
| WP_013241104.1 | *Cp1002B_1235* | Hypothetical protein | 1.99 | -0.99080062 | 0.000258088 | DOWN |
| WP_013241147.1 | *ccsA* | Cytochrome c biogenesis protein ccsa | 1.92 | 0.94304670 | 0.002894992 | UP |
| WP_013241172.1 | *Cp1002B_1590* | Membrane protein | 1.33 | -0.40746306 | 0.026664296 | REG |
| WP_014300461.1 | *Cp1002B_1680* | Hypothetical protein | 1.86 | -0.89842111 | 0.023503716 | DOWN |
| [WP_014401012.1](http://www.ncbi.nlm.nih.gov/protein/WP_014401012.1) | *Cp1002B_2240* | Membrane protein | 1.35 | 0.42901158 | 0.022217473 | REG |
| WP_013241310.1 | *htaA* | Cell-surface hemin receptor | 2.70 | 1.43267925 | 0.000128406 | UP |
| WP_013241311.1 | *hmuT* | ABC transporter substrate-binding protein | 3.04 | 1.60250271 | 5.47E-05 | UP |
| WP_013241312.1 | *hmuU* | Hemin import atp-binding protein | 2.09 | 1.06660938 | 0.005645516 | UP |
| WP_013241314.1 | *htaC* | Hypothetical protein | 7.41 | 2.89015069 | 3.33E-11 | UP |
| [WP_014300514.1](http://www.ncbi.nlm.nih.gov/protein/WP_014300514.1) | *WhiB* | Whib family transcriptional regulator | 1.59 | 0.66647696 | 0.020631043 | UP |
| [WP_013242872.1](http://www.ncbi.nlm.nih.gov/protein/WP_013242872.1) | *Cp1002B_2935* | Transglycosylase associated protein | 1.75 | -0.80851436 | 3.39E-05 | DOWN |
| [WP_013242864.1](http://www.ncbi.nlm.nih.gov/protein/WP_013242864.1) | *hrrA* | DNA-binding response regulator | 1.99 | 0.98936897 | 4.02E-05 | UP |
| WP_014522292.1 | *Cp1002B_3070* | Hypothetical protein | 22.90 | 4.51740393 | 3.62E-26 | UP |
| WP_013242841.1 | *Cp1002B_3075* | Hypothetical protein | 28.20 | 4.81767729 | 2.43E-32 | UP |
|  | *Cp1002B_3725* | FUSC family protein;pseudo | 1.51 | 0.59106213 | 2.37E-05 | UP |
| WP_014522271.1 | *Cp1002B_3730* | Hypothetical protein | 1.66 | 0.72793303 | 0.00335166 | UP |
| [WP_013242698.1](http://www.ncbi.nlm.nih.gov/protein/WP_013242698.1) | *ccsB* | Cytochrome C biogenesis protein | 1.41 | -0.49961433 | 0.044619839 | REG |
| [WP_013242669.1](http://www.ncbi.nlm.nih.gov/protein/WP_013242669.1) | *Cp1002B_3905* | Cation:proton antiporter | 1.60 | -0.67886689 | 0.02554289 | DOWN |
| [WP_013242602.1](http://www.ncbi.nlm.nih.gov/protein/WP_013242602.1) | *IlvB1* | Acetolactate synthase large subunit ilvb1 | 1.64 | 0.71442304 | 0.04042813 | UP |
| WP_013242586.1 | *Cp1002B_4315* | Hypothetical protein | 1.94 | 0.95247557 | 0.001833731 | UP |
| WP_014300921.1 | *Cp1002B_4550* | Antimicrobial peptide ABC transporter | 1.40 | -0.48765514 | 0.003452582 | REG |
| WP_013242430.1 | *rpsT* | 30S ribosomal protein S20 | 1.52 | 0.60224891 | 0.008322644 | UP |
| [WP_013242381.1](http://www.ncbi.nlm.nih.gov/protein/WP_013242381.1) | *Cp1002B_5410* | Membrane protein | 1.38 | 0.46069178 | 0.037698195 | REG |
| WP_013242364.1 | *Cp1002B_5505* | Hypothetical protein | 1.66 | 0.73058897 | 8.84E-06 | UP |
| [WP_013242345.1](http://www.ncbi.nlm.nih.gov/protein/WP_013242345.1) | *Cp1002B_5600* | Hypothetical protein | 1.49 | -0.57305480 | 0.028963703 | REG |
| WP_013242332.1 | *htaF* | Cell-surface hemin receptor | 7.29 | 2.86676168 | 2.69E-14 | UP |
| WP_013242331.1 | *htaG* | Uncharacterized protein htac | 5.86 | 2.55071095 | 0.002597513 | UP |
| WP_013242283.1 | *ctaC* | Cytochrome c oxidase subunit II | 2.10 | -1.06697640 | 1.06E-06 | DOWN |
| WP_013242282.1 | *ctaF* | Cytochrome c oxidase polypeptide 4 | 2.13 | -1.08882811 | 1.08E-07 | DOWN |
| WP_013242281.1 | *ctaE* | Heme-copper oxidase subunit III;Cytochrome c oxidase subunit III | 2.61 | -1.38133384 | 7.24E-11 | DOWN |
| WP_013242280.1 | *qcrC* | Ubiquinol-cytochrome C reductase cytochrome C subunit | 2.57 | -1.36030789 | 1.02E-09 | DOWN |
| WP_014300818.1 | *qcrA* | Ubiquinol-cytochrome c reductase iron-sulfur subunit | 2.18 | -1.12369409 | 0.000567675 | DOWN |
| WP_013242278.1 | *qcrB* | Ubiquinol-cytochrome C reductase cytochrome B subunit | 2.44 | -1.28647712 | 6.48E-07 | DOWN |
| WP_013242277.1 | *Cp1002B_5915* | Hypothetical protein, npl/p60 family rote | 2.10 | 1.06894276 | 1.59E-11 | UP |
| WP_013242223.1 | *Cp1002B_6180* | Hypothetical protein | 1.51 | 0.59905564 | 2.43E-06 | UP |
| WP_013242184.1 | *rpsP* | 30S ribosomal protein S16 | 1.68 | 0.75001067 | 0.006012873 | UP |
| WP_013242169.1 | *rpsB* | 30S ribosomal protein S2 | 1.86 | 0.89254186 | 0.011455716 | UP |
| WP_038616474.1 | *mapB* | methionine aminopeptidase | 1.26 | 0.33579134 | 0.043912281 | REG |
| WP_013242135.1 | *rbfA* | Ribosome-binding factor A | 1.54 | -0.61916475 | 0.002597513 | DOWN |
| WP_013242127.1 | *rpsO* | 30S ribosomal protein S15 | 1.66 | 0.72694547 | 0.000258088 | UP |
| [WP_013242121.1](http://www.ncbi.nlm.nih.gov/protein/WP_013242121.1) | *Cp1002B_6680* | Hypothetical protein | 1.41 | -0.49825912 | 0.018377372 | REG |
| WP_013242080.1 | *ahpD* | Alkyl hydroperoxide reductase | 1.45 | 0.53549987 | 0.002212884 | REG |
| WP_013241996.1 | *Cp1002B_7300* | Uncharacterized peptidase yqht | 1.36 | 0.44186931 | 0.045562918 | REG |
| [WP_013241994.1](http://www.ncbi.nlm.nih.gov/protein/WP_013241994.1) | *nusB* | Transcription antitermination protein nusb | 1.31 | 0.39293454 | 0.044444147 | REG |
| WP_013241968.1 | *rapZ* | Rnase adaptor protein rapz | 1.32 | 0.40055688 | 0.026878656 | REG |
| WP_013241958.1 | *zwf* | Glucose-6-phosphate dehydrogenase | 1.41 | 0.49092795 | 0.006012873 | REG |
| WP_013241957.1 | *tal* | Transaldolase | 1.27 | 0.34747651 | 0.023121339 | REG |
| WP_013241950.1 | *sufR* | Arsr family transcriptional regulator;Iron-sulfur cluster biosynthesis transcriptional regulator sufr | 1.65 | 0.72331015 | 0.000883021 | UP |
| WP_013241891.1 | *pafB* | Protein pafb | 1.54 | 0.62541924 | 0.001833731 | UP |
| WP_013241870.1 | *ndh* | NAD(P)/FAD-dependent oxidoreductase | 1.58 | 0.66087849 | 0.002206551 | UP |
| WP_014522824.1 | *gnd* | Phosphogluconate dehydrogenase | 1.43 | 0.51097812 | 0.00479576 | REG |
| [WP_013241858.1](http://www.ncbi.nlm.nih.gov/protein/WP_013241858.1) | *Cp1002B_7990* | Hypothetical protein | 1.35 | 0.43456513 | 0.049631926 | REG |
| WP_057061524.1 | *Cp1002B_8515* | Hypothetical protein | 1.55 | 0.63109337 | 0.014090059 | UP |
| [WP_038616439.1](http://www.ncbi.nlm.nih.gov/protein/WP_038616439.1) | *Cp1002B_8520* | Hypothetical protein | 1.56 | 0.63873835 | 0.003003536 | UP |
| WP_014300634.1 | *czcD* | Cadmium, cobalt and zinc/H(+)-K(+) antiporter | 1.98 | 0.98243851 | 2.57E-05 | UP |
| WP_013241750.1 | *ilvC* | Ketol-acid reductoisomerase (NADP(+)) | 1.42 | 0.50216481 | 0.026701234 | REG |
| WP_013241696.1 | *ywlC* | Trna threonylcarbamoyladenosine biosynthesis protein ywlc | 1.52 | 0.60023873 | 3.39E-05 | UP |
| WP_013241682.1 | *Cp1002B_8855* | Hypothetical protein | 1.95 | 0.96684071 | 9.02E-05 | UP |
| WP_014366861.1 | *GlpR* | Glycerol-3-phosphate regulon repressor | 1.64 | 0.71631969 | 0.007008324 | UP |
| WP_013241665.1 | *galT* | Galactose-1-phosphate uridylyltransferase | 1.45 | 0.53877654 | 0.003194642 | REG |
| WP_013241622.1 | *Cp1002B_9145* | Hypothetical protein | 1.43 | 0.51586838 | 0.002894992 | REG |
| WP_013241575.1 | *Cp1002B_9380* | Hypothetical protein | 1.74 | 0.80159961 | 1.05E-08 | UP |
| WP_039697516.1 | *ripA* | Arac family transcriptional regulator-HTH-type transcriptional repressor of iron protein A | 2.44 | 1.28829471 | 6.08E-13 | UP |
| WP_013241560.1 | *Cp1002B_9460* | Ribose-phosphate pyrophosphokinase | 1.33 | -0.40905274 | 0.014460429 | REG |
| WP_013241539.1 | *Cp1002B_9550* | Antibiotic biosynthesis monooxygenase | 1.51 | 0.59258290 | 0.004739101 | UP |
| WP_013241535.1 | *rpfB* | Resuscitation-promoting factor rpfb | 1.70 | 0.76406592 | 1.58E-07 | UP |
| [WP_013241514.1](http://www.ncbi.nlm.nih.gov/protein/WP_013241514.1) | *rpmE2* | 50S ribosomal protein L31 | 1.38 | 0.46891228 | 0.006379392 | REG |
| WP_013241482.1 | *Cp1002B_9845* | ABC transporter substrate-binding protein | 1.47 | 0.56022364 | 0.013264456 | REG |
| WP_013241465.1 | *fkbP* | Peptidyl-prolyl cis-trans isomerase, FKBP-type | 1.61 | -0.68715082 | 0.01323332 | DOWN |
| WP_013242913.1 | *Cp1002B_10210* | Substrate-binding protein | 2.44 | -1.28477155 | 1.28E-07 | DOWN |
| WP_013240910.1 | *Cp1002B_10495* | Hypothetical protein | 6.30 | 2.65529734 | 2.66E-14 | UP |
| WP_014300401.1 | *Cp1002B_10510* | Hypothetical protein | 1.80 | 0.85097217 | 0.013271231 | UP |
| [WP_076761485.1](https://www.ncbi.nlm.nih.gov/projects/sviewer/sequence.cgi?id=gi\|1140912577&format=fasta&filename=WP_076761485.1.fa&ranges=0-73) | *Cp1002B_10545* | Hypothetical protein | 1.81 | 0.85570870 | 0.04264298 | UP |
| WP_014300577.1 | *Cp1002B_10785* | Hypothetical protein | 5.93 | 2.56871970 | 3.23E-32 | UP |
|  | *Cp1002B_0615* | tRNA | 1.70 | -0.7692 | 0.01323332 | DOWN |
|  | *Cp1002B_0730* | tRNA | 1.73 | -0.7923 | 0.013271231 | DOWN |
|  | *Cp1002B_2115* | tRNA | 1.42 | 0.5092 | 0.020078668 | REG |
|  | *Cp1002B_4495* | tRNA | 2.68 | 1.4241 | 6.08E-06 | UP |
|  | *Cp1002B_7685* | tRNA | 2.24 | -1.1651 | 6.48E-07 | DOWN |
|  | *Cp1002B_9390* | tRNA | 1.69 | 0.7547 | 0.017203604 | UP |
