# Supplementary material for: Transcriptome profile of Corynebacterium pseudotuberculosis in response to iron limitation
Source: BMC Genomics. 2019 Aug 20;20:663. doi: 10.1186/s12864-019-6018-1 (PMC6701010; doi:10.1186/s12864-019-6018-1)
Supplement: Supplementary file 4 — Table S7. Table of gene ontology terms assigned to DEGs identified in the T1 strain. Table S8. Table of gene ontology terms assigned to DEGs identified in the Cp13 mutant (DOCX 32 kb) [file 12864_2019_6018_MOESM4_ESM.docx]

Additional file 4

**Transcriptome Profile of *Corynebacterium pseudotuberculosis* in Response to Iron Limitation**

Izabela Coimbra Ibraim^1^, Mariana Teixeira Dornelles Parise^1^, Doglas Parise^1^, Michelle Zibetti Tadra Sfeir^2^, Thiago Luiz de Paula Castro^3^, Alice Rebecca Wattan^4^, Preetam Ghosh^5^, Debmalya Barh^1^, Emannuel Maltempi Souza^2^, Aristóteles Góes-Neto^6^, Anne Cybelle Pinto Gomide^a1^, Vasco Azevedo^a1*^

Corresponding Author: Dr. Vasco Azevedo - vasco@icb.ufmg.br

Table of Contents:

Table S7. Table of gene ontology terms assigned to DEGs identified in the T1 strain

Table S8. Table of gene ontology terms assigned to DEGs identified in the Cp13 mutant

Table S7. Table of gene ontology terms assigned to DEGs identified in the T1 strain. Genes have been divided in up and downregulated and classified into biological process, cellular function and molecular function categories. The GO annotations for DEGs were submitted to GOfeat for ontology classification.

| Sequence Name | GO Count | Annotation GO ID | Annotation GO Term |
| --- | --- | --- | --- |
| WP_013241191.1 | 4 | GO:0003735;GO:0005840;GO:0006412;GO:0019843 | structural constituent of ribosome;  ribosome;  translation;  rRNA binding |
| WP_013910826.1 | 4 | GO:0003735;GO:0005840;GO:0006412;GO:0019843 | structural constituent of ribosome;  ribosome;  translation;  rRNA binding |
| WP_013241511.1 | 4 | GO:0003735;GO:0005840;GO:0006412;GO:0019843 | structural constituent of ribosome;  ribosome;  translation;  rRNA binding |
| WP_013241209.1 | 5 | GO:0003729;GO:0003735;GO:0005840;GO:0006412;GO:0019843 | mRNA binding;  structural constituent of ribosome;  ribosome;  translation;  rRNA binding |
| WP_013241261.1 | 3 | GO:0003735;GO:0005840;GO:0006412 | structural constituent of ribosome;  ribosome;  translation |
| WP_014300452.1 | 5 | GO:0003735;GO:0005840;GO:0006412;GO:0019843;GO:0042254 | structural constituent of ribosome;  ribosome;  translation;  rRNA binding;  ribosome biogenesis |
| WP_013241260.1 | 3 | GO:0003735;GO:0005840;GO:0006412 | structural constituent of ribosome;  ribosome;  translation |
| WP_013242446.1 | 3 | GO:0003735;GO:0005840;GO:0006412 | structural constituent of ribosome;  ribosome;  translation |
| WP_013241171.1 | 3 | GO:0003735;GO:0005840;GO:0006412 | structural constituent of ribosome;  ribosome;  translation |
| WP_013241312.1 | 2 | GO:0005886;GO:0043167 | plasma membrane;  ion binding |
| WP_013241311.1 |  |  | hypothetical protein |
| WP_013242547.1 | 1 | GO:0005886 | plasma membrane |
| WP_013242245.1 | 3 | GO:0005737;GO:0007049;GO:0051301 | cytoplasm;  cell cycle;  cell division |
| WP_013241310.1 | 1 | GO:0005575 | Cellular component |
| WP_013242332.1 | 1 | GO:0005575 | Cellular component |
| WP_013241593.1 | 5 | GO:0005737;GO:0006091;GO:0016829;GO:0032991;GO:0044281 | cytoplasm;  generation of precursor metabolites and energy;  lyase activity;  protein-containing complex;  small molecule metabolic process |
| WP_004566891.1 | 3 | GO:0005737;GO:0006457;GO:0043167 | cytoplasm;  protein folding;  ion binding |
| WP_013241065.1 | 3 | GO:0003677;GO:0009058;GO:0034641 | DNA binding;  biosynthetic process;  cellular nitrogen compound metabolic process |
| WP_013241147.1 | 3 | GO:0003674;GO:0005575;GO:0065003 | Molecular function;  Cellular component;  protein-containing complex assembly |
| CP1002_2885 | 6 | GO:0006520;GO:0006790;GO:0009058;GO:0016765;GO:0016829;GO:0043167 | cellular amino acid metabolic process;  sulfur compound metabolic process;  biosynthetic process;  transferase activity |
| WP_013242281.1 | 4 | GO:0005886;GO:0006091;GO:0016491;GO:0022857 | plasma membrane;  generation of precursor metabolites and energy;  oxidoreductase activity;  transmembrane transporter activity |
| WP_013242283.1 | 5 | GO:0005886;GO:0006091;GO:0016491;GO:0022857;GO:0043167 | plasma membrane;  generation of precursor metabolites and energy;  oxidoreductase activity;  transmembrane transporter activity;  ion binding |
| WP_013242280.1 | 4 | GO:0005886;GO:0006091;GO:0016491;GO:0043167 | plasma membrane;  generation of precursor metabolites and energy;  oxidoreductase activity;  ion binding |
| WP_013242282.1 | 5 | GO:0005886;GO:0006091;GO:0016491;GO:0016874;GO:0022857 | plasma membrane;  generation of precursor metabolites and energy;  oxidoreductase activity;  ligase activity;  transmembrane transporter activity |
| WP_013242523.1 | 5 | GO:0005886;GO:0006091;GO:0016491;GO:0022857;GO:0043167 | plasma membrane;  generation of precursor metabolites and energy;  oxidoreductase activity;  transmembrane transporter activity;  ion binding |
| WP_046341090.1 | 1 | GO:0016746 | transferase activity |
| WP_013241099.1 | 4 | GO:0006091;GO:0016491;GO:0042592;GO:0043167 | generation of precursor metabolites and energy;  oxidoreductase activity;  homeostatic process;  ion binding |
| WP_013242261.1 | 6 | GO:0003677;GO:0003700;GO:0005737;GO:0009058;GO:0034641;GO:0051301 | DNA binding;  DNA-binding transcription factor activity;  cytoplasm;  biosynthetic process;  cellular nitrogen compound metabolic process;  cell division |
| WP_013242864.1 | 4 | GO:0003677;GO:0007165;GO:0009058;GO:0034641 | DNA binding;  signal transduction;  biosynthetic process;  cellular nitrogen compound metabolic process |
| WP_013241193.1 | 4 | GO:0003924;GO:0005737;GO:0008135;GO:0043167 | GTPase activity;  cytoplasm;  translation factor activity |
| WP_013242168.1 | 2 | GO:0005737;GO:0008135 | cytoplasm;  translation factor activity |
| WP_013241701.1 | 8 | GO:0005622;GO:0005886;GO:0009058;GO:0016887;GO:0022857;GO:0032991;GO:0034641;GO:0044281 | intracellular;  plasma membrane;  biosynthetic process;  ATPase activity;  transmembrane transporter activity;  protein-containing complex;  cellular nitrogen compound metabolic process;  small molecule metabolic process |
| WP_014522179.1 | 8 | GO:0005622;GO:0005886;GO:0009058;GO:0016887;GO:0022857;GO:0032991;GO:0034641;GO:0044281 | intracellular;  plasma membrane;  biosynthetic process;  ATPase activity;  transmembrane transporter activity;  protein-containing complex;  cellular nitrogen compound metabolic process;  small molecule metabolic process |
| WP_014367533.1 | 5 | GO:0005737;GO:0006810;GO:0016491;GO:0042592;GO:0043167 | cytoplasm;  transport;  oxidoreductase activity;  homeostatic process;  ion binding |
| WP_013242872.1 | 1 | GO:0005575 | Cellular component |
| WP_013241314.1 | 1 | GO:0005575 | Cellular component |
| WP_014522292.1 | 1 | GO:0005575 | Cellular component |
| WP_013242331.1 | 1 | GO:0005575 | Cellular component |
| WP_039697516.1 | 4 | GO:0003677;GO:0003700;GO:0009058;GO:0034641 | DNA binding;  DNA-binding transcription factor activity;  biosynthetic process;  cellular nitrogen compound metabolic process |
| WP_013241684.1 | 2 | GO:0003674;GO:0044281 | Molecular function;  small molecule metabolic process |
| WP_013241683.1 |  |  | hypothetical protein |
| WP_013242841.1 | 1 | GO:0005575 | Cellular component |
| WP_013242278.1 | 4 | GO:0005886;GO:0006091;GO:0016491;GO:0043167 | plasma membrane;  generation of precursor metabolites and energy;  oxidoreductase activity;  ion binding |
| WP_014300818.1 | 3 | GO:0005886;GO:0016491;GO:0043167 | plasma membrane;  oxidoreductase activity;  ion binding |
| WP_013241276.1 | 4 | GO:0005737;GO:0006457;GO:0043167;GO:0051082 | cytoplasm;  protein folding;  ion binding;  unfolded protein binding |
| WP_013241103.1 | 2 | GO:0006091;GO:0016491 | generation of precursor metabolites and energy;  oxidoreductase activity |
| WP_013241104.1 |  |  | hypothetical protein |
| WP_013241378.1 | 1 | GO:0008150 | Biological process |
| WP_003402602.1 |  |  | hypothetical protein |
| WP_013240878.1 | 3 | GO:0006457;GO:0006464;GO:0016853 | protein folding;  cellular protein modification process;  isomerase activity |
| WP_013241465.1 | 2 | GO:0006464;GO:0016853 | cellular protein modification process;  isomerase activity |
| WP_013242515.1 | 2 | GO:0005575;GO:0008233 | Cellular component;  peptidase activity |
| WP_013242135.1 | 3 | GO:0005737;GO:0034641;GO:0042254 | cytoplasm;  cellular nitrogen compound metabolic process;  ribosome biogenesis |
| WP_013240983.1 | 3 | GO:0003674;GO:0005575;GO:0055085 | Molecular function; cellular component;  transmembrane transport |
| WP_013242913.1 | 1 | GO:0005575 | Cellular component |
| WP_013241101.1 | 2 | GO:0005575;GO:0016491 | Cellular component; oxidoreductase activity |
| WP_013241102.1 | 1 | GO:0016491 | oxidoreductase activity |
| WP_013242852.1 | 3 | GO:0006950;GO:0016491;GO:0043167 | response to stress;  oxidoreductase activity;  ion binding |
| WP_013242880.1 | 3 | GO:0016491;GO:0042592;GO:0044281 | oxidoreductase activity;  homeostatic process;  small molecule metabolic process |
| WP_013241696.1 | 1 | GO:0003723 | RNA binding |
| WP_013240933.1 |  |  | hypothetical protein |
| WP_013242461.1 | 7 | GO:0005737;GO:0006457;GO:0006464;GO:0007049;GO:0015031;GO:0016853;GO:0051301 | cytoplasm;  protein folding;  cellular protein modification process;  cell cycle;  protein transport;  isomerase activity;  cell division |
| WP_013241263.1 |  |  | hypothetical protein |
| WP_013242563.1 |  |  | hypothetical protein |
| WP_013240877.1 | 1 | GO:0005575 | Cellular component |
| WP_013242924.1 |  |  | hypothetical protein |
| WP_014300577.1 |  |  | hypothetical protein |
| WP_013241266.1 |  |  | hypothetical protein |
| WP_014300695.1 | 1 | GO:0003677 | DNA binding |

Table S8. Table of gene ontology terms assigned to DEGs identified in the Cp13 mutant. Genes have been divided in up and downregulated and classified into the biological process, cellular function and molecular function categories. The GO annotations for DEGs were submitted to GOfeat for ontology classification.

| Sequence Name | GO Count | Annotation GO ID | Annotation GO Term |
| --- | --- | --- | --- |
| WP_013242859.1 | 4 | GO:0009058;GO:0034655;GO:0043167;GO:0044281 | biosynthetic process;  nucleobase-containing compound catabolic process;  ion binding;  small molecule metabolic process |
| WP_014300503.1 | 5 | GO:0009058;GO:0016757;GO:0034641;GO:0043167;GO:0044281 | biosynthetic process;  transferase activity, transferring glycosyl groups;  cellular nitrogen compound metabolic process;  ion binding;  small molecule metabolic process |
| WP_013242127.1 | 4 | GO:0003735;GO:0005840;GO:0006412;GO:0019843 | structural constituent of ribosome;  ribosome;  translation;  rRNA binding |
| WP_013242430.1 | 4 | GO:0003735;GO:0005840;GO:0006412;GO:0019843 | structural constituent of ribosome;  ribosome;  translation;  rRNA binding |
| WP_013242864.1 | 4 | GO:0003677;GO:0007165;GO:0009058;GO:0034641 | DNA binding;  signal transduction;  biosynthetic process;  cellular nitrogen compound metabolic process |
| WP_014366861.1 | 4 | GO:0003677;GO:0003700;GO:0009058;GO:0034641 | DNA binding;  DNA-binding transcription factor activity;  biosynthetic process;  cellular nitrogen compound metabolic process |
| WP_039697516.1 | 4 | GO:0003677;GO:0003700;GO:0009058;GO:0034641 | DNA binding;  DNA-binding transcription factor activity;  biosynthetic process;  cellular nitrogen compound metabolic process |
| WP_014300514.1 | 5 | GO:0003677;GO:0005737;GO:0009058;GO:0034641;GO:0043167 | DNA binding;  cytoplasm;  biosynthetic process;  cellular nitrogen compound metabolic process;  ion binding |
| WP_013240910.1 |  |  | hypothetical protein |
| WP_013241310.1 | 1 | GO:0005575 | Cellular component |
| WP_013241314.1 | 1 | GO:0005575 | Cellular component |
| WP_013241539.1 | 1 | GO:0016491 | oxidoreductase activity |
| WP_013241575.1 | 1 | GO:0005575 | Cellular component |
| WP_013241682.1 |  |  | Uncharacterized protein |
| WP_013241870.1 | 2 | GO:0005575;GO:0016491 | Cellular component;  oxidoreductase activity |
| WP_013241950.1 | 3 | GO:0003700;GO:0009058;GO:0034641 | DNA-binding transcription factor activity;  biosynthetic process;  cellular nitrogen compound metabolic process |
| WP_013242169.1 | 3 | GO:0003735;GO:0005840;GO:0006412 | structural constituent of ribosome;  ribosome;  translation |
| WP_013242184.1 | 3 | GO:0003735;GO:0005840;GO:0006412 | structural constituent of ribosome;  ribosome;  translation |
| WP_013242331.1 | 1 | GO:0005575 | Cellular component |
| WP_013242332.1 | 1 | GO:0005575 | Cellular component |
| WP_013242364.1 |  |  | hypothetical protein |
| WP_013242841.1 | 1 | GO:0005575 | Cellular component |
| WP_014300401.1 |  |  | Uncharacterized protein |
| WP_014300577.1 |  |  | hypothetical protein |
| WP_014300634.1 | 2 | GO:0005575;GO:0022857 | Cellular component  transmembrane transporter activity |
| WP_014522292.1 | 1 | GO:0005575 | Cellular component |
| WP_076761485.1 |  |  | Uncharacterized protein |
| WP_013242602.1 | 2 | GO:0016491;GO:0043167 | oxidoreductase activity;  ion binding |
| WP_013242278.1 | 4 | GO:0005886;GO:0006091;GO:0016491;GO:0043167 | plasma membrane;  generation of precursor metabolites and energy;  oxidoreductase activity;  ion binding |
| WP_013242280.1 | 4 | GO:0005886;GO:0006091;GO:0016491;GO:0043167 | plasma membrane;  generation of precursor metabolites and energy;  oxidoreductase activity;  ion binding |
| WP_013242283.1 | 5 | GO:0005886;GO:0006091;GO:0016491;GO:0022857;GO:0043167 | plasma membrane;  generation of precursor metabolites and energy;  oxidoreductase activity;  transmembrane transporter activity;  ion binding |
| WP_013242281.1 | 4 | GO:0005886;GO:0006091;GO:0016491;GO:0022857 | plasma membrane;  generation of precursor metabolites and energy;  oxidoreductase activity;  transmembrane transporter activity |
| WP_013242282.1 | 5 | GO:0005886;GO:0006091;GO:0016491;GO:0016874;GO:0022857 | plasma membrane;  generation of precursor metabolites and energy;  oxidoreductase activity;  ligase activity;  transmembrane transporter activity |
| WP_013241084.1 | 2 | GO:0003677;GO:0005737 | DNA binding;  cytoplasm |
| WP_013241147.1 | 3 | GO:0003674;GO:0005575;GO:0065003 | Molecular function;  Cellular component;  protein-containing complex assembly |
| WP_013241104.1 | 1 | GO:0005575 | Cellular component |
| WP_013241465.1 | 2 | GO:0006464;GO:0016853 | cellular protein modification process;  isomerase activity |
| WP_013242135.1 | 3 | GO:0005737;GO:0034641;GO:0042254 | cytoplasm;  cellular nitrogen compound metabolic process;  ribosome biogenesis |
| WP_013242872.1 | 1 | GO:0005575 | Cellular component |
| WP_013242913.1 | 1 | GO:0005575 | Cellular component |
| WP_014300461.1 |  |  | Uncharacterized protein |
| WP_014300818.1 | 3 | GO:0005886;GO:0016491;GO:0043167 | plasma membrane;  oxidoreductase activity;  ion binding |
| WP_013242669.1 | 2 | GO:0005575;GO:0022857 | Cellular component;  transmembrane transporter activity |
