# Supplementary material for: Transcriptome profile of Corynebacterium pseudotuberculosis in response to iron limitation
Source: BMC Genomics. 2019 Aug 20;20:663. doi: 10.1186/s12864-019-6018-1 (PMC6701010; doi:10.1186/s12864-019-6018-1)

Additional file 5

**Transcriptome Profile of *Corynebacterium pseudotuberculosis* in Response to Iron Limitation**

Izabela Coimbra Ibraim^1^, Mariana Teixeira Dornelles Parise^1^, Doglas Parise^1^, Michelle Zibetti Tadra Sfeir^2^, Thiago Luiz de Paula Castro^3^, Alice Rebecca Wattan^4^, Preetam Ghosh^5^, Debmalya Barh^1^, Emannuel Maltempi Souza^2^, Aristóteles Góes-Neto^6^, Anne Cybelle Pinto Gomide^a1^, Vasco Azevedo^a1*^

Corresponding Author: Dr. Vasco Azevedo - vasco@icb.ufmg.br

Table of Contents:

Table S9. Predicted protein-protein interactions of iron regulated genes identified in the wild-type T1 strain

Table S10. Predicted protein-protein interactions of iron regulated genes identified in the Cp13 mutant

Figure S4. Protein enrichment analyses. Tables with enriched pathways identified in the (**A**) T1 strain and (**B**) Cp13 mutant of *C. pseudotuberculosis*.

Table S9. PPI of iron regulated genes in the T1 strain. Using protein interaction network analysis tool (STRING database - https://string-db.org) showing 298 edges and 75 nodes with a PPI enrichment score of 1,6e-16. Functional protein association are derived from (1) neighborhood on chromosome, (2) gene fusion, (3) phylogenetic cooccurrence, (4) homology, (5) co-expression, (6) experimentally determined interaction, (7) database annotated and (8) automated text mining. A total combine confidence score is computed for each interaction and it is used as the final measure for building the network interactions. We show 111 interactions with a combine interaction score of >0.4 (medium confidence), 58 with scores greater than 0.7 (high confidence) and 129 with scores over 0.9 (highest confidence). The high number of interactions with a confidence score greater than 0.9 indicate a high protein-protein interaction with a significant biological association within the DEGs predicted products identified in the T1 strain.

| PPI T1 strain | | | | | | | | | | |
| --- | --- | --- | --- | --- | --- | --- | --- | --- | --- | --- |
| Node 1 | **Node 2** | 1 | 2 | 3 | 4 | 5 | 6 | 7 | 8 | Total |
| ctaC | **ctaD** | 0.277 | 0.229 | 0.744 | 0 | 0.971 | 0.929 | 0.8 | 0.886 | 0.999 |
| rpsC | **rplM** | 0.231 | 0 | 0.167 | 0 | 0.899 | 0.929 | 0 | 0.886 | 0.999 |
| rpsC | **rplP** | 0.9 | 0.779 | 0.498 | 0 | 0.971 | 0.929 | 0 | 0.554 | 0.999 |
| atpH | **atpF** | 0.9 | 0.675 | 0.184 | 0 | 0.96 | 0.795 | 0.8 | 0.497 | 0.999 |
| rpsL | **rpsG** | 0.9 | 0.77 | 0.479 | 0 | 0.971 | 0.929 | 0 | 0.554 | 0.999 |
| rplV | **rpsC** | 0.9 | 0.715 | 0.318 | 0 | 0.971 | 0.929 | 0 | 0.497 | 0.999 |
| rplV | **rplP** | 0.9 | 0 | 0.253 | 0 | 0.971 | 0.929 | 0 | 0.497 | 0.999 |
| atpA | **atpH** | 0.782 | 0 | 0.157 | 0 | 0.942 | 0.929 | 0.8 | 0.534 | 0.999 |
| rpsS | **rplV** | 0.9 | 0 | 0.277 | 0 | 0.971 | 0.929 | 0 | 0.497 | 0.999 |
| ctaE | **ctaD** | 0.264 | 0.699 | 0.784 | 0 | 0.971 | 0.82 | 0.8 | 0.887 | 0.999 |
| rplJ | **rplL** | 0.758 | 0.754 | 0.306 | 0 | 0.971 | 0.929 | 0 | 0.554 | 0.999 |
| atpA | **atpF** | 0.782 | 0 | 0.19 | 0 | 0.971 | 0.929 | 0.8 | 0.554 | 0.999 |
| rpsG | **rplP** | 0.277 | 0 | 0.749 | 0 | 0.934 | 0.929 | 0 | 0.497 | 0.999 |
| qcrA | **qcrB** | 0.9 | 0 | 0.747 | 0 | 0.613 | 0.828 | 0.8 | 0.891 | 0.999 |
| qcrC | **qcrB** | 0.9 | 0 | 0.768 | 0 | 0.146 | 0 | 0.8 | 0.878 | 0.999 |
| rpsS | **rpsC** | 0.9 | 0 | 0.5 | 0 | 0.971 | 0.929 | 0 | 0.554 | 0.999 |
| groS | **groEL** | 0.895 | 0 | 0.694 | 0 | 0.837 | 0.375 | 0 | 0.8 | 0.999 |
| rpsL | **rpsS** | 0.277 | 0 | 0.682 | 0 | 0.85 | 0.981 | 0 | 0.45 | 0.999 |
| qcrC | **qcrA** | 0.9 | 0 | 0.771 | 0 | 0.119 | 0 | 0.8 | 0.878 | 0.999 |
| rplM | **rpsI** | 0.9 | 0.716 | 0.592 | 0 | 0.971 | 0.929 | 0 | 0.554 | 0.999 |
| rpsS | **rplP** | 0.9 | 0 | 0.65 | 0 | 0.971 | 0.929 | 0 | 0.554 | 0.999 |
| sdhA | **sdhB** | 0.9 | 0 | 0.784 | 0 | 0.945 | 0.87 | 0.8 | 0.86 | 0.999 |
| lutB | **Cp_8845** | 0.9 | 0 | 0.78 | 0 | 0.971 | 0.296 | 0 | 0.851 | 0.999 |
| ctaC | **ctaE** | 0.274 | 0 | 0.755 | 0 | 0.942 | 0.929 | 0.8 | 0.887 | 0.999 |
| rplV | **rplM** | 0.231 | 0 | 0.46 | 0 | 0.888 | 0.929 | 0 | 0.497 | 0.998 |
| rpsG | **rpsS** | 0.277 | 0 | 0.484 | 0 | 0.934 | 0.929 | 0 | 0.497 | 0.998 |
| rplJ | **rpsC** | 0.231 | 0 | 0 | 0 | 0.852 | 0.929 | 0 | 0.851 | 0.998 |
| rpsG | **rpsC** | 0.277 | 0 | 0.595 | 0 | 0.878 | 0.929 | 0 | 0.497 | 0.998 |
| rpsL | **rplP** | 0.277 | 0 | 0.666 | 0 | 0.856 | 0.929 | 0 | 0.497 | 0.998 |
| rpsG | **rplM** | 0.231 | 0 | 0.238 | 0 | 0.93 | 0.929 | 0 | 0.497 | 0.998 |
| rplJ | **rpsG** | 0.231 | 0 | 0.233 | 0 | 0.924 | 0.929 | 0 | 0.497 | 0.998 |
| rplP | **rplM** | 0.231 | 0 | 0.347 | 0 | 0.913 | 0.929 | 0 | 0.554 | 0.998 |
| ctaE | **qcrB** | 0.751 | 0 | 0.517 | 0 | 0.848 | 0.356 | 0 | 0.789 | 0.997 |
| rpsG | **rplV** | 0.277 | 0 | 0.265 | 0 | 0.903 | 0.929 | 0 | 0.497 | 0.997 |
| ctaC | **qcrB** | 0.255 | 0 | 0.731 | 0 | 0.847 | 0.356 | 0 | 0.873 | 0.997 |
| fusA | **rplV** | 0.277 | 0 | 0.271 | 0 | 0.957 | 0.817 | 0 | 0.497 | 0.997 |
| rpsS | **rpsI** | 0.231 | 0 | 0.271 | 0 | 0.864 | 0.929 | 0 | 0.554 | 0.997 |
| rplL | **rplM** | 0.115 | 0 | 0.491 | 0 | 0.841 | 0.929 | 0 | 0.554 | 0.997 |
| rpsL | **rpsC** | 0.277 | 0 | 0.473 | 0 | 0.848 | 0.929 | 0 | 0.497 | 0.997 |
| sdhC | **sdhB** | 0.874 | 0 | 0.781 | 0 | 0 | 0 | 0.8 | 0.57 | 0.997 |
| rpsS | **rplM** | 0.231 | 0 | 0.184 | 0 | 0.885 | 0.929 | 0 | 0.554 | 0.997 |
| hmuT | **hmuU** | 0.853 | 0 | 0.573 | 0 | 0.064 | 0.289 | 0.8 | 0.788 | 0.997 |
| rpmA | **rplM** | 0.231 | 0 | 0.462 | 0 | 0.839 | 0.929 | 0 | 0.554 | 0.997 |
| rpmA | **rpsI** | 0.231 | 0 | 0.406 | 0 | 0.838 | 0.929 | 0 | 0.554 | 0.997 |
| rpsC | **rpsI** | 0.231 | 0 | 0.186 | 0 | 0.856 | 0.929 | 0 | 0.554 | 0.996 |
| rplP | **rpsI** | 0.231 | 0 | 0.229 | 0 | 0.859 | 0.929 | 0 | 0.554 | 0.996 |
| rpmA | **rplL** | 0.049 | 0 | 0.549 | 0 | 0.836 | 0.903 | 0 | 0.554 | 0.996 |
| rpsG | **fusA** | 0.627 | 0.003 | 0.345 | 0 | 0.946 | 0.512 | 0 | 0.519 | 0.996 |
| rplV | **rpsI** | 0.231 | 0 | 0.294 | 0 | 0.867 | 0.929 | 0 | 0.497 | 0.996 |
| rpsL | **rplM** | 0.231 | 0 | 0.246 | 0 | 0.87 | 0.929 | 0 | 0.497 | 0.996 |
| rplL | **rpsG** | 0.231 | 0 | 0.241 | 0 | 0.839 | 0.929 | 0 | 0.497 | 0.996 |
| rpsL | **fusA** | 0.626 | 0 | 0.398 | 0 | 0.877 | 0.793 | 0 | 0.554 | 0.996 |
| rpsL | **rpsI** | 0.231 | 0 | 0.283 | 0 | 0.83 | 0.929 | 0 | 0.497 | 0.996 |
| rplJ | **rplP** | 0.231 | 0 | 0.154 | 0 | 0.882 | 0.929 | 0 | 0.497 | 0.996 |
| rpsL | **rplV** | 0.277 | 0 | 0 | 0 | 0.856 | 0.929 | 0 | 0.497 | 0.995 |
| rpsR | **rplP** | 0 | 0 | 0.177 | 0 | 0.859 | 0.929 | 0 | 0.497 | 0.995 |
| rpsR | **rplM** | 0 | 0 | 0.176 | 0 | 0.862 | 0.929 | 0 | 0.497 | 0.995 |
| sdhC | **sdhA** | 0.874 | 0 | 0.78 | 0 | 0 | 0 | 0.8 | 0.233 | 0.995 |
| rplL | **rpsC** | 0.231 | 0 | 0.166 | 0 | 0.811 | 0.929 | 0 | 0.554 | 0.995 |
| rplL | **rplV** | 0.231 | 0 | 0.224 | 0 | 0.808 | 0.929 | 0 | 0.497 | 0.995 |
| rplL | **rplP** | 0.231 | 0 | 0.332 | 0 | 0.836 | 0.901 | 0 | 0.554 | 0.995 |
| rplJ | **rpsL** | 0.231 | 0 | 0 | 0 | 0.844 | 0.929 | 0 | 0.497 | 0.995 |
| rplJ | **rpsS** | 0.231 | 0 | 0.15 | 0 | 0.848 | 0.929 | 0 | 0.497 | 0.995 |
| rpsC | **rpsM** | 0.266 | 0 | 0.501 | 0 | 0.819 | 0.506 | 0 | 0.886 | 0.995 |
| rplJ | **rplV** | 0.231 | 0 | 0 | 0 | 0.85 | 0.929 | 0 | 0.497 | 0.995 |
| rplJ | **rplM** | 0.096 | 0 | 0 | 0 | 0.848 | 0.929 | 0 | 0.497 | 0.994 |
| rpsG | **rpsI** | 0.231 | 0 | 0.266 | 0 | 0.767 | 0.929 | 0 | 0.497 | 0.994 |
| fusA | **rplP** | 0.277 | 0 | 0.327 | 0 | 0.908 | 0.806 | 0 | 0.496 | 0.994 |
| rpsR | **rpsI** | 0 | 0 | 0 | 0 | 0.857 | 0.929 | 0 | 0.497 | 0.994 |
| rpsR | **rpsC** | 0 | 0 | 0 | 0 | 0.852 | 0.929 | 0 | 0.497 | 0.994 |
| rpmA | **rplV** | 0 | 0 | 0.216 | 0 | 0.818 | 0.929 | 0 | 0.4 | 0.993 |
| rpsR | **rplV** | 0 | 0 | 0.155 | 0 | 0.865 | 0.929 | 0 | 0.239 | 0.993 |
| rpsR | **rpsS** | 0 | 0 | 0 | 0 | 0.858 | 0.929 | 0 | 0.452 | 0.993 |
| rpsR | **rplJ** | 0 | 0 | 0 | 0 | 0.824 | 0.929 | 0 | 0.497 | 0.993 |
| rpsR | **rpsG** | 0.097 | 0 | 0.182 | 0 | 0.807 | 0.929 | 0 | 0.497 | 0.993 |
| fusA | **rplM** | 0.231 | 0 | 0.525 | 0 | 0.832 | 0.806 | 0 | 0.497 | 0.992 |
| fusA | **rpsC** | 0.277 | 0 | 0.182 | 0 | 0.877 | 0.817 | 0 | 0.497 | 0.992 |
| rplJ | **rpsI** | 0.062 | 0 | 0 | 0 | 0.808 | 0.929 | 0 | 0.497 | 0.992 |
| rpmA | **rpsG** | 0.045 | 0 | 0.203 | 0 | 0.78 | 0.929 | 0 | 0.497 | 0.992 |
| rpmA | **rpsR** | 0 | 0 | 0.24 | 0 | 0.81 | 0.929 | 0 | 0.26 | 0.991 |
| rpsR | **rpsL** | 0.088 | 0 | 0 | 0 | 0.766 | 0.929 | 0 | 0.497 | 0.991 |
| rpsR | **rplL** | 0 | 0 | 0 | 0 | 0.804 | 0.929 | 0 | 0.397 | 0.99 |
| fusA | **rpsS** | 0.277 | 0 | 0.188 | 0 | 0.86 | 0.806 | 0 | 0.497 | 0.99 |
| rpmA | **rplJ** | 0.046 | 0 | 0 | 0 | 0.766 | 0.929 | 0 | 0.497 | 0.99 |
| aceF | **lpd** | 0.277 | 0.02 | 0.474 | 0 | 0.316 | 0.324 | 0.9 | 0.554 | 0.99 |
| rpmA | **rplP** | 0 | 0 | 0.442 | 0 | 0.492 | 0.929 | 0 | 0.554 | 0.989 |
| rplL | **rpsI** | 0.1 | 0 | 0.218 | 0 | 0.824 | 0.817 | 0 | 0.553 | 0.988 |
| fusA | **rpsI** | 0.231 | 0 | 0.623 | 0 | 0.613 | 0.817 | 0 | 0.497 | 0.987 |
| rplP | **rpsM** | 0.267 | 0 | 0.613 | 0 | 0.837 | 0.506 | 0 | 0.554 | 0.987 |
| rpmA | **rpsC** | 0 | 0 | 0 | 0 | 0.628 | 0.929 | 0 | 0.554 | 0.987 |
| rpsS | **rpsM** | 0.264 | 0 | 0.677 | 0 | 0.832 | 0.375 | 0 | 0.554 | 0.986 |
| rplL | **rpsL** | 0.231 | 0 | 0.222 | 0 | 0.788 | 0.806 | 0 | 0.497 | 0.985 |
| rpmA | **rpsL** | 0.045 | 0 | 0.255 | 0 | 0.663 | 0.929 | 0 | 0.248 | 0.984 |
| rplL | **fusA** | 0.231 | 0 | 0.229 | 0 | 0.804 | 0.776 | 0 | 0.496 | 0.984 |
| qcrB | **ctaD** | 0.154 | 0 | 0.411 | 0 | 0.846 | 0.322 | 0 | 0.728 | 0.983 |
| atpA | **fusA** | 0.042 | 0 | 0.28 | 0 | 0.784 | 0 | 0 | 0.887 | 0.981 |
| rpsM | **rplM** | 0.129 | 0 | 0.224 | 0 | 0.779 | 0.506 | 0 | 0.79 | 0.981 |
| rplJ | **fusA** | 0.231 | 0 | 0 | 0 | 0.842 | 0.817 | 0 | 0.202 | 0.979 |
| rpmA | **rpsS** | 0 | 0 | 0.164 | 0 | 0.229 | 0.929 | 0 | 0.554 | 0.976 |
| htaA | **hmuT** | 0.649 | 0 | 0.742 | 0 | 0 | 0 | 0 | 0.759 | 0.976 |
| fumC | **sdhA** | 0.231 | 0 | 0 | 0 | 0.388 | 0.123 | 0.9 | 0.497 | 0.975 |
| ctaE | **qcrA** | 0.752 | 0 | 0.24 | 0 | 0.093 | 0.326 | 0 | 0.805 | 0.973 |
| tsf | **fusA** | 0.1 | 0 | 0.25 | 0 | 0.764 | 0.335 | 0 | 0.787 | 0.973 |
| fumC | **sdhB** | 0.231 | 0 | 0 | 0 | 0.332 | 0.115 | 0.9 | 0.497 | 0.972 |
| ctaC | **qcrA** | 0.255 | 0 | 0.576 | 0 | 0.28 | 0.326 | 0 | 0.827 | 0.968 |
| rplL | **rpsS** | 0.231 | 0 | 0.151 | 0 | 0.475 | 0.817 | 0 | 0.554 | 0.966 |
| rpsL | **rpsM** | 0.13 | 0 | 0.537 | 0 | 0.826 | 0.506 | 0 | 0.164 | 0.965 |
| htaA | **htaC** | 0.376 | 0 | 0.783 | 0 | 0 | 0 | 0 | 0.757 | 0.964 |
| ctaC | **ctaF** | 0.866 | 0 | 0.495 | 0 | 0.064 | 0 | 0 | 0.482 | 0.963 |
| hmuT | **htaC** | 0.541 | 0 | 0.662 | 0 | 0 | 0 | 0 | 0.757 | 0.959 |
| rplV | **rpsM** | 0.263 | 0 | 0.273 | 0 | 0.837 | 0.506 | 0 | 0.164 | 0.957 |
| tsf | **rplM** | 0.231 | 0 | 0.155 | 0 | 0.865 | 0 | 0 | 0.554 | 0.955 |
| rpsG | **rpsM** | 0.125 | 0 | 0.374 | 0 | 0.813 | 0.506 | 0 | 0.164 | 0.949 |
| ctaE | **qcrC** | 0.752 | 0 | 0.313 | 0 | 0.079 | 0.372 | 0 | 0.554 | 0.948 |
| rpsM | **rpsI** | 0.108 | 0 | 0.363 | 0 | 0.64 | 0.506 | 0 | 0.554 | 0.946 |
| tsf | **rpsI** | 0.231 | 0 | 0.17 | 0 | 0.833 | 0 | 0 | 0.535 | 0.943 |
| rpsR | **fusA** | 0.099 | 0 | 0 | 0 | 0.618 | 0.817 | 0 | 0.157 | 0.939 |
| rplJ | **rpsM** | 0.098 | 0 | 0 | 0 | 0.754 | 0.375 | 0 | 0.593 | 0.936 |
| tsf | **rpsC** | 0.099 | 0 | 0.182 | 0 | 0.828 | 0 | 0 | 0.546 | 0.934 |
| fusA | **rpsM** | 0.136 | 0 | 0.348 | 0 | 0.804 | 0.373 | 0 | 0.173 | 0.932 |
| tsf | **rplP** | 0.098 | 0 | 0 | 0 | 0.839 | 0 | 0 | 0.554 | 0.929 |
| tsf | **rpsS** | 0.1 | 0 | 0 | 0 | 0.82 | 0 | 0 | 0.554 | 0.921 |
| rpmA | **rpsM** | 0 | 0 | 0.193 | 0 | 0.609 | 0.506 | 0 | 0.554 | 0.921 |
| qcrA | **ctaD** | 0.15 | 0 | 0.187 | 0 | 0.077 | 0.314 | 0 | 0.848 | 0.921 |
| rplP | **hpf** | 0 | 0 | 0 | 0 | 0.064 | 0.383 | 0 | 0.86 | 0.912 |
| htaA | **hmuU** | 0.558 | 0 | 0.22 | 0 | 0 | 0 | 0 | 0.757 | 0.909 |
| rpmA | **fusA** | 0 | 0 | 0.346 | 0 | 0.289 | 0.793 | 0 | 0.152 | 0.907 |
| rplL | **rpsM** | 0.098 | 0 | 0.166 | 0 | 0.61 | 0.373 | 0 | 0.554 | 0.903 |
| fumC | **sdhC** | 0.044 | 0 | 0 | 0 | 0 | 0 | 0.9 | 0 | 0.9 |
| rpsR | **rpsM** | 0 | 0 | 0 | 0 | 0.765 | 0.506 | 0 | 0.181 | 0.896 |
| qcrC | **ctaD** | 0.231 | 0 | 0.2 | 0 | 0.066 | 0.351 | 0 | 0.762 | 0.895 |
| qcrA | **sdhB** | 0.06 | 0 | 0 | 0 | 0.846 | 0.122 | 0 | 0.261 | 0.893 |
| atpA | **sdhA** | 0.049 | 0 | 0 | 0 | 0.759 | 0 | 0 | 0.508 | 0.877 |
| tsf | **rpsG** | 0.1 | 0 | 0 | 0 | 0.864 | 0 | 0 | 0.078 | 0.877 |
| tsf | **rplV** | 0.101 | 0 | 0 | 0 | 0.846 | 0 | 0 | 0.157 | 0.873 |
| atpA | **fumC** | 0 | 0 | 0 | 0 | 0.799 | 0.124 | 0 | 0.316 | 0.869 |
| tsf | **rplJ** | 0 | 0 | 0.179 | 0 | 0.837 | 0 | 0 | 0.098 | 0.868 |
| ctaC | **qcrC** | 0.257 | 0 | 0.475 | 0 | 0.064 | 0.304 | 0 | 0.554 | 0.866 |
| qcrA | **atpH** | 0 | 0 | 0 | 0 | 0.844 | 0 | 0 | 0.158 | 0.863 |
| tsf | **rpsR** | 0 | 0 | 0 | 0 | 0.836 | 0 | 0 | 0.201 | 0.863 |
| qcrA | **atpA** | 0 | 0 | 0 | 0 | 0.834 | 0 | 0 | 0.199 | 0.861 |
| tig | **tsf** | 0.069 | 0 | 0 | 0 | 0.807 | 0.126 | 0 | 0.205 | 0.858 |
| hmuU | **htaC** | 0.526 | 0 | 0.191 | 0 | 0 | 0 | 0 | 0.656 | 0.856 |
| tsf | **rpsL** | 0.1 | 0 | 0 | 0 | 0.811 | 0 | 0 | 0.2 | 0.852 |
| Cp_3075 | **Cp_3070** | 0.821 | 0 | 0 | 0 | 0 | 0 | 0 | 0.201 | 0.851 |
| tsf | **rpsM** | 0.056 | 0 | 0 | 0 | 0.698 | 0 | 0 | 0.516 | 0.849 |
| rpmA | **tsf** | 0.052 | 0 | 0 | 0 | 0.671 | 0 | 0 | 0.554 | 0.848 |
| atpA | **rpsC** | 0.076 | 0 | 0 | 0 | 0.791 | 0.127 | 0 | 0.201 | 0.847 |
| qcrA | **sdhA** | 0.097 | 0 | 0 | 0 | 0.768 | 0.195 | 0 | 0.202 | 0.847 |
| atpF | **rplV** | 0 | 0 | 0 | 0 | 0.845 | 0 | 0 | 0.049 | 0.846 |
| ctaF | **ctaE** | 0.088 | 0 | 0.252 | 0 | 0.066 | 0 | 0 | 0.778 | 0.839 |
| ctaF | **qcrA** | 0.093 | 0 | 0.683 | 0 | 0.064 | 0 | 0 | 0.471 | 0.838 |
| tig | **rplJ** | 0 | 0 | 0.467 | 0 | 0.289 | 0.171 | 0 | 0.54 | 0.836 |
| atpF | **rpsC** | 0 | 0 | 0 | 0 | 0.826 | 0 | 0 | 0.092 | 0.835 |
| tsf | **rplL** | 0 | 0 | 0 | 0 | 0.612 | 0.115 | 0 | 0.554 | 0.833 |
| atpA | **rplP** | 0.079 | 0 | 0.152 | 0 | 0.76 | 0.115 | 0 | 0.134 | 0.83 |
| atpA | **rplV** | 0.062 | 0 | 0 | 0 | 0.805 | 0.115 | 0 | 0.06 | 0.827 |
| sdhC | **Cp_1235** | 0.764 | 0 | 0.29 | 0 | 0 | 0 | 0 | 0 | 0.826 |
| rplL | **groS** | 0 | 0 | 0 | 0 | 0.67 | 0 | 0 | 0.497 | 0.826 |
| hmuU | **htaF** | 0 | 0 | 0 | 0 | 0.064 | 0 | 0.8 | 0.05 | 0.806 |
| sodA | **groS** | 0.056 | 0 | 0 | 0 | 0.428 | 0.294 | 0 | 0.548 | 0.804 |
| Cp_3070 | **htaA** | 0.097 | 0 | 0.767 | 0 | 0 | 0 | 0 | 0.137 | 0.802 |
| hmuT | **htaF** | 0 | 0 | 0 | 0 | 0 | 0 | 0.8 | 0 | 0.8 |
| sdhB | **Cp_1235** | 0.785 | 0 | 0 | 0 | 0 | 0 | 0 | 0.049 | 0.788 |
| htaG | **htaA** | 0.079 | 0 | 0.78 | 0 | 0 | 0 | 0 | 0 | 0.788 |
| sdhA | **Cp_1235** | 0.785 | 0 | 0 | 0 | 0 | 0 | 0 | 0 | 0.786 |
| atpF | **fusA** | 0 | 0 | 0 | 0 | 0.776 | 0 | 0 | 0.065 | 0.781 |
| ctaF | **qcrC** | 0.094 | 0 | 0.732 | 0 | 0.066 | 0 | 0 | 0.104 | 0.769 |
| qcrB | **sdhA** | 0.062 | 0 | 0 | 0 | 0.143 | 0 | 0 | 0.716 | 0.751 |
| rplV | **groS** | 0 | 0 | 0 | 0 | 0.611 | 0 | 0 | 0.385 | 0.75 |
| atpH | **rplM** | 0 | 0 | 0 | 0 | 0.75 | 0 | 0 | 0 | 0.75 |
| ripA | **rpfA** | 0 | 0 | 0 | 0 | 0 | 0 | 0 | 0.749 | 0.749 |
| qcrB | **sdhB** | 0.046 | 0 | 0 | 0 | 0.131 | 0 | 0 | 0.717 | 0.745 |
| atpF | **rpsS** | 0 | 0 | 0 | 0 | 0.691 | 0 | 0 | 0.201 | 0.742 |
| ctaF | **qcrB** | 0.061 | 0 | 0.662 | 0 | 0.066 | 0 | 0 | 0.232 | 0.741 |
| atpA | **rpsG** | 0 | 0 | 0.165 | 0 | 0.66 | 0 | 0 | 0.158 | 0.74 |
| atpA | **rpsL** | 0 | 0 | 0.281 | 0 | 0.612 | 0 | 0 | 0.127 | 0.735 |
| htaG | **Cp_10210** | 0 | 0 | 0.735 | 0 | 0 | 0 | 0 | 0 | 0.735 |
| htaF | **htaG** | 0.477 | 0 | 0.496 | 0 | 0 | 0 | 0 | 0.045 | 0.727 |
| atpA | **rpsS** | 0.062 | 0 | 0 | 0 | 0.683 | 0 | 0 | 0.148 | 0.724 |
| Cp_10210 | **htaC** | 0 | 0 | 0.72 | 0 | 0 | 0 | 0 | 0 | 0.72 |
| rpmA | **atpH** | 0.147 | 0 | 0 | 0 | 0.663 | 0 | 0 | 0.094 | 0.716 |
| tig | **rpsI** | 0.043 | 0 | 0 | 0 | 0.667 | 0.171 | 0 | 0.049 | 0.715 |
| atpA | **rpsM** | 0.083 | 0 | 0 | 0 | 0.656 | 0.115 | 0 | 0.083 | 0.709 |
| tig | **rplM** | 0.043 | 0 | 0 | 0 | 0.663 | 0.171 | 0 | 0 | 0.709 |
| atpH | **rplL** | 0 | 0 | 0 | 0 | 0.454 | 0 | 0 | 0.483 | 0.705 |
| Cp_95 | **Cp_350** | 0 | 0 | 0.705 | 0 | 0 | 0 | 0 | 0 | 0.705 |
| atpH | **sdhB** | 0.096 | 0 | 0 | 0 | 0.612 | 0 | 0 | 0.205 | 0.696 |
| atpF | **rpsG** | 0 | 0 | 0 | 0 | 0.63 | 0 | 0 | 0.2 | 0.691 |
| rpsG | **groS** | 0 | 0 | 0 | 0 | 0.608 | 0 | 0 | 0.234 | 0.686 |
| htaF | **hmuT** | 0.098 | 0 | 0.609 | 0 | 0.064 | 0 | 0 | 0.161 | 0.685 |
| atpH | **rplV** | 0.062 | 0 | 0 | 0 | 0.657 | 0 | 0 | 0.102 | 0.685 |
| atpA | **groS** | 0.046 | 0 | 0 | 0 | 0.28 | 0 | 0 | 0.573 | 0.681 |
| Cp_10210 | **htaA** | 0 | 0 | 0.68 | 0 | 0 | 0 | 0 | 0 | 0.68 |
| htaG | **hmuT** | 0.092 | 0 | 0.662 | 0 | 0 | 0 | 0 | 0 | 0.679 |
| tig | **rpsR** | 0 | 0 | 0 | 0 | 0.677 | 0 | 0 | 0 | 0.677 |
| tsf | **atpA** | 0 | 0 | 0 | 0 | 0.612 | 0 | 0 | 0.204 | 0.677 |
| tig | **rpsG** | 0.049 | 0 | 0.176 | 0 | 0.407 | 0.348 | 0 | 0.072 | 0.667 |
| Cp_3070 | **htaC** | 0.057 | 0 | 0.656 | 0 | 0 | 0 | 0 | 0.045 | 0.663 |
| sodA | **rpsL** | 0 | 0 | 0 | 0 | 0.084 | 0.3 | 0 | 0.516 | 0.662 |
| atpF | **rpsM** | 0 | 0 | 0 | 0 | 0.623 | 0 | 0 | 0.133 | 0.659 |
| rplV | **hpf** | 0 | 0 | 0.355 | 0 | 0 | 0.383 | 0 | 0.206 | 0.656 |
| atpH | **rpsM** | 0.083 | 0 | 0 | 0 | 0.634 | 0 | 0 | 0 | 0.65 |
| htaF | **htaC** | 0.057 | 0 | 0.638 | 0 | 0 | 0 | 0 | 0.045 | 0.645 |
| Cp_3070 | **Cp_10210** | 0 | 0 | 0.643 | 0 | 0 | 0 | 0 | 0 | 0.643 |
| Cp_350 | **Cp_95** | 0 | 0 | 0.643 | 0 | 0 | 0 | 0 | 0 | 0.643 |
| tig | **rplV** | 0.049 | 0 | 0.158 | 0 | 0.526 | 0.171 | 0 | 0 | 0.643 |
| atpF | **rplP** | 0 | 0 | 0 | 0 | 0.609 | 0 | 0 | 0.12 | 0.641 |
| qcrA | **fumC** | 0 | 0 | 0 | 0 | 0.612 | 0.115 | 0 | 0 | 0.641 |
| atpH | **sodA** | 0 | 0 | 0 | 0 | 0.607 | 0 | 0 | 0.125 | 0.641 |
| tig | **fusA** | 0.052 | 0 | 0 | 0 | 0.292 | 0 | 0 | 0.497 | 0.632 |
| tig | **rpsS** | 0.048 | 0 | 0 | 0 | 0.546 | 0.171 | 0 | 0.093 | 0.631 |
| atpA | **rplJ** | 0 | 0 | 0 | 0 | 0.512 | 0 | 0 | 0.266 | 0.626 |
| fumC | **tcsR** | 0.047 | 0 | 0 | 0 | 0 | 0 | 0 | 0.624 | 0.626 |
| qcrA | **ag84** | 0 | 0 | 0.622 | 0 | 0 | 0 | 0 | 0 | 0.622 |
| tig | **atpF** | 0.061 | 0 | 0 | 0 | 0.607 | 0 | 0 | 0 | 0.615 |
| Cp_95 | **ppiA** | 0.606 | 0 | 0 | 0 | 0 | 0 | 0 | 0 | 0.606 |
| tig | **rplP** | 0.049 | 0 | 0 | 0 | 0.482 | 0.171 | 0 | 0.149 | 0.605 |
| tig | **rpsC** | 0.049 | 0 | 0 | 0 | 0.462 | 0.171 | 0 | 0.16 | 0.596 |
| rplM | **groS** | 0.082 | 0 | 0 | 0 | 0.414 | 0 | 0 | 0.311 | 0.596 |
| ctaF | **ctaD** | 0.277 | 0 | 0.172 | 0 | 0.064 | 0 | 0 | 0.357 | 0.591 |
| aceF | **sdhB** | 0.098 | 0 | 0 | 0 | 0.421 | 0.17 | 0 | 0.162 | 0.588 |
| rpmA | **sodA** | 0 | 0 | 0 | 0 | 0.438 | 0.295 | 0 | 0 | 0.586 |
| ripA | **ftn** | 0 | 0 | 0.227 | 0 | 0 | 0 | 0 | 0.487 | 0.586 |
| fumC | **sodA** | 0.142 | 0 | 0 | 0 | 0.228 | 0.189 | 0 | 0.313 | 0.581 |
| tig | **groS** | 0.06 | 0 | 0 | 0 | 0.094 | 0 | 0 | 0.536 | 0.57 |
| fusA | **groS** | 0 | 0 | 0 | 0 | 0.078 | 0 | 0 | 0.553 | 0.57 |
| atpF | **sdhA** | 0 | 0 | 0 | 0 | 0.086 | 0 | 0 | 0.545 | 0.566 |
| ctaC | **atpA** | 0 | 0 | 0 | 0 | 0.504 | 0 | 0 | 0.157 | 0.563 |
| aceF | **sdhA** | 0.098 | 0 | 0 | 0 | 0.423 | 0.115 | 0 | 0.164 | 0.563 |
| atpA | **sdhB** | 0.092 | 0 | 0 | 0 | 0.381 | 0 | 0 | 0.276 | 0.557 |
| htaG | **Cp_3070** | 0.057 | 0 | 0.542 | 0 | 0 | 0 | 0 | 0.045 | 0.551 |
| rplP | **groS** | 0 | 0 | 0 | 0 | 0.231 | 0 | 0 | 0.434 | 0.546 |
| qcrB | **atpA** | 0 | 0 | 0 | 0 | 0.132 | 0 | 0 | 0.496 | 0.543 |
| sodA | **groEL** | 0.058 | 0 | 0 | 0 | 0.154 | 0.115 | 0 | 0.426 | 0.541 |
| atpA | **ctaD** | 0 | 0 | 0 | 0 | 0.444 | 0 | 0 | 0.201 | 0.536 |
| atpH | **sdhA** | 0.05 | 0 | 0 | 0 | 0.156 | 0 | 0 | 0.466 | 0.534 |
| Cp_3070 | **hmuT** | 0.098 | 0 | 0.418 | 0 | 0.064 | 0 | 0 | 0.161 | 0.532 |
| tsf | **groS** | 0.049 | 0 | 0 | 0 | 0.1 | 0 | 0 | 0.495 | 0.53 |
| fusA | **groEL** | 0 | 0 | 0.159 | 0 | 0.12 | 0.214 | 0 | 0.285 | 0.528 |
| atpF | **rpsL** | 0 | 0 | 0 | 0 | 0.493 | 0 | 0 | 0.106 | 0.527 |
| ctaC | **sdhB** | 0 | 0 | 0 | 0 | 0.278 | 0.177 | 0 | 0.262 | 0.523 |
| tig | **fkbP** | 0 | 0 | 0 | 0 | 0.091 | 0 | 0 | 0.496 | 0.522 |
| atpA | **rpsR** | 0 | 0 | 0 | 0 | 0.471 | 0 | 0 | 0.129 | 0.519 |
| tsf | **atpH** | 0.056 | 0 | 0 | 0 | 0.407 | 0 | 0 | 0.205 | 0.516 |
| atpF | **rplL** | 0 | 0 | 0 | 0 | 0.158 | 0 | 0 | 0.444 | 0.511 |
| tig | **atpA** | 0 | 0 | 0 | 0 | 0.411 | 0 | 0 | 0.203 | 0.51 |
| qcrB | **ccsA** | 0 | 0 | 0.429 | 0 | 0.066 | 0 | 0 | 0.155 | 0.51 |
| rpsG | **hpf** | 0 | 0 | 0 | 0 | 0 | 0.383 | 0 | 0.234 | 0.507 |
| rpfA | **glxR** | 0 | 0 | 0 | 0 | 0 | 0 | 0 | 0.504 | 0.504 |
| rpmA | **groS** | 0.056 | 0 | 0 | 0 | 0.276 | 0 | 0 | 0.321 | 0.495 |
| tig | **rplL** | 0 | 0 | 0 | 0 | 0.284 | 0.171 | 0 | 0.218 | 0.495 |
| ctaC | **sdhA** | 0 | 0 | 0 | 0 | 0.342 | 0 | 0 | 0.262 | 0.493 |
| atpA | **lpd** | 0.087 | 0.001 | 0 | 0 | 0.407 | 0 | 0 | 0.12 | 0.482 |
| atpF | **rplM** | 0 | 0 | 0 | 0 | 0.411 | 0 | 0 | 0.157 | 0.482 |
| atpH | **rpsC** | 0.079 | 0 | 0 | 0 | 0.431 | 0 | 0 | 0.092 | 0.482 |
| tig | **rpmA** | 0.101 | 0 | 0 | 0 | 0.431 | 0 | 0 | 0.067 | 0.481 |
| tsf | **atpF** | 0.056 | 0 | 0 | 0 | 0.373 | 0 | 0 | 0.191 | 0.479 |
| sodA | **rpsI** | 0.061 | 0 | 0 | 0 | 0.277 | 0.295 | 0 | 0 | 0.479 |
| sodA | **rplM** | 0.061 | 0 | 0 | 0 | 0.291 | 0.278 | 0 | 0 | 0.477 |
| atpF | **rpsR** | 0 | 0 | 0 | 0 | 0.457 | 0 | 0 | 0.068 | 0.472 |
| atpF | **rplJ** | 0 | 0 | 0 | 0 | 0.339 | 0 | 0 | 0.234 | 0.472 |
| lpd | **sdhA** | 0.087 | 0 | 0 | 0 | 0.274 | 0.184 | 0 | 0.138 | 0.471 |
| htaF | **Cp_10210** | 0 | 0 | 0.466 | 0 | 0 | 0 | 0 | 0 | 0.466 |
| sdhA | **ctaD** | 0 | 0 | 0 | 0 | 0.286 | 0.124 | 0 | 0.214 | 0.465 |
| lutB | **sdhB** | 0.231 | 0 | 0 | 0 | 0.146 | 0 | 0 | 0.25 | 0.464 |
| atpA | **rplL** | 0 | 0 | 0.153 | 0 | 0.151 | 0 | 0 | 0.309 | 0.459 |
| ctaF | **Cp_1235** | 0 | 0 | 0.457 | 0 | 0 | 0 | 0 | 0 | 0.457 |
| aceF | **atpA** | 0.051 | 0 | 0 | 0 | 0.371 | 0 | 0 | 0.157 | 0.452 |
| tig | **rpsL** | 0.049 | 0 | 0 | 0 | 0.429 | 0 | 0 | 0.066 | 0.448 |
| sodA | **rplP** | 0 | 0 | 0 | 0 | 0.279 | 0.265 | 0 | 0 | 0.447 |
| qcrA | **rplL** | 0 | 0 | 0 | 0 | 0.343 | 0 | 0 | 0.186 | 0.442 |
| lpd | **sdhB** | 0.088 | 0 | 0 | 0 | 0.229 | 0.197 | 0 | 0.128 | 0.441 |
| Cp_3070 | **Cp_095** | 0 | 0 | 0.438 | 0 | 0 | 0 | 0 | 0 | 0.438 |
| rplJ | **hpf** | 0.042 | 0 | 0.16 | 0 | 0 | 0.357 | 0 | 0 | 0.437 |
| atpA | **rplM** | 0 | 0 | 0 | 0 | 0.413 | 0 | 0 | 0.074 | 0.433 |
| atpH | **rplJ** | 0 | 0 | 0 | 0 | 0.413 | 0 | 0 | 0.075 | 0.433 |
| glxR | **sdhC** | 0 | 0 | 0 | 0 | 0 | 0 | 0 | 0.431 | 0.431 |
| Cp_95 | **Cp_1235** | 0 | 0 | 0.429 | 0 | 0 | 0 | 0 | 0 | 0.429 |
| qcrA | **sodA** | 0.26 | 0 | 0 | 0 | 0.195 | 0 | 0 | 0.117 | 0.428 |
| sodA | **rpsG** | 0 | 0 | 0 | 0 | 0.229 | 0.284 | 0 | 0 | 0.424 |
| glxR | **sdhB** | 0.153 | 0 | 0 | 0 | 0.076 | 0 | 0 | 0.322 | 0.423 |
| qcrA | **rplM** | 0.231 | 0 | 0 | 0 | 0.28 | 0 | 0 | 0 | 0.422 |
| Cp_10210 | **hmuT** | 0 | 0 | 0.421 | 0 | 0 | 0 | 0 | 0 | 0.421 |
| rplM | **hpf** | 0.079 | 0 | 0 | 0 | 0.064 | 0.383 | 0 | 0 | 0.421 |
| tsf | **rbfA** | 0.231 | 0 | 0 | 0 | 0.125 | 0 | 0 | 0.205 | 0.418 |
| aceF | **qcrA** | 0 | 0 | 0 | 0 | 0.414 | 0 | 0 | 0 | 0.414 |
| rpsC | **groS** | 0 | 0 | 0 | 0 | 0.177 | 0 | 0 | 0.318 | 0.414 |
| htaF | **htaA** | 0.097 | 0 | 0.77 | 0.58 | 0 | 0 | 0 | 0.137 | 0.411 |
| rpsI | **hpf** | 0.062 | 0 | 0 | 0 | 0.065 | 0.383 | 0 | 0 | 0.411 |
| ripA | **Cp_95** | 0 | 0 | 0.41 | 0 | 0 | 0 | 0 | 0 | 0.41 |
| aceF | **fumC** | 0 | 0 | 0 | 0 | 0.228 | 0.115 | 0 | 0.205 | 0.409 |
| ppiA | **fusA** | 0.049 | 0 | 0 | 0 | 0.065 | 0.348 | 0 | 0.096 | 0.405 |
| fusA | **htaA** | 0 | 0 | 0 | 0 | 0 | 0.37 | 0 | 0.095 | 0.405 |
| Cp_2040 | **Cp_2050** | 0.403 | 0 | 0 | 0 | 0 | 0 | 0 | 0 | 0.404 |
| rpsM | **groS** | 0.045 | 0 | 0 | 0 | 0.305 | 0 | 0 | 0.172 | 0.402 |
| tig | **groEL** | 0.048 | 0 | 0 | 0 | 0.094 | 0.231 | 0 | 0.204 | 0.401 |
| ctaC | **ccsA** | 0.049 | 0 | 0.281 | 0 | 0.092 | 0 | 0 | 0.148 | 0.4 |

Additional file 5: Table S10. PPI of iron regulated genes in the Cp13 mutant. Using protein interaction network analysis tool (STRING database - https://string-db.org) showing 94 edges and 57 nodes with a PPI enrichment score of 6.02E-14. Functional protein association are derived from (1) neighborhood on chromosome, (2) gene fusion, (3) phylogenetic cooccurrence, (4) homology, (5) co-expression, (6) experimentally determined interaction, (7) database annotated and (8) automated text mining. A total combine confidence score is computed for each interaction and it is used as the final measure for building the network interactions. We show 35 interactions with a combine interaction score of >0.4 (medium confidence), 18 with scores greater than 0.7 (high confidence) and 41 with scores over 0.9 (highest confidence). The high number of interactions with a confidence score greater than 0.9 indicate a high protein-protein interaction with a significant biological association within the DEGs predicted products identified in the Cp13 strain.

| PPI Cp13 strain | | | | | | | | | | |
| --- | --- | --- | --- | --- | --- | --- | --- | --- | --- | --- |
| Node 1 | **Node 2** | 1 | 2 | 3 | 4 | 5 | 6 | 7 | 8 | Total |
| *hmuT* | ***hmuV*** | 0.894 | 0 | 0.735 | 0 | 0.093 | 0.33 | 0.8 | 0.85 | 0.999 |
| *ctaE* | ***ctaB*** | 0.231 | 0.269 | 0.781 | 0 | 0.794 | 0 | 0.8 | 0.849 | 0.999 |
| *ctaE* | ***ctaD*** | 0.264 | 0.699 | 0.784 | 0 | 0.971 | 0.82 | 0.8 | 0.887 | 0.999 |
| *qcrC* | ***qcrB*** | 0.9 | 0 | 0.768 | 0 | 0.146 | 0 | 0.8 | 0.878 | 0.999 |
| *rpsG* | ***rplP*** | 0.277 | 0 | 0.749 | 0 | 0.934 | 0.929 | 0 | 0.497 | 0.999 |
| *hmuU* | ***hmuV*** | 0.853 | 0.043 | 0.715 | 0 | 0.117 | 0.322 | 0.9 | 0.788 | 0.999 |
| *ctaC* | ***ctaE*** | 0.274 | 0 | 0.755 | 0 | 0.942 | 0.929 | 0.8 | 0.887 | 0.999 |
| *qcrA* | ***qcrB*** | 0.9 | 0 | 0.747 | 0 | 0.613 | 0.828 | 0.8 | 0.891 | 0.999 |
| *ctaC* | ***ctaD*** | 0.277 | 0.229 | 0.744 | 0 | 0.971 | 0.929 | 0.8 | 0.886 | 0.999 |
| *qcrC* | ***qcrA*** | 0.9 | 0 | 0.771 | 0 | 0.119 | 0 | 0.8 | 0.878 | 0.999 |
| *ctaC* | ***ctaB*** | 0.231 | 0 | 0.728 | 0 | 0.71 | 0.178 | 0.8 | 0.883 | 0.998 |
| *rpsB* | ***rplP*** | 0.103 | 0 | 0.741 | 0 | 0.851 | 0.929 | 0 | 0.554 | 0.998 |
| *rpsB* | ***rpsG*** | 0.106 | 0 | 0.659 | 0 | 0.909 | 0.929 | 0 | 0.497 | 0.998 |
| *ctaB* | ***ctaD*** | 0.231 | 0 | 0.781 | 0 | 0.832 | 0.139 | 0.8 | 0.818 | 0.998 |
| *ctaE* | ***qcrB*** | 0.751 | 0 | 0.517 | 0 | 0.848 | 0.356 | 0 | 0.789 | 0.997 |
| *hmuT* | ***hmuU*** | 0.853 | 0 | 0.573 | 0 | 0.064 | 0.289 | 0.8 | 0.788 | 0.997 |
| *ctaC* | ***qcrB*** | 0.255 | 0 | 0.731 | 0 | 0.847 | 0.356 | 0 | 0.873 | 0.997 |
| *rpsB* | ***rpsO*** | 0.231 | 0 | 0.239 | 0 | 0.845 | 0.929 | 0 | 0.497 | 0.996 |
| *rpsT* | ***rpsO*** | 0.231 | 0 | 0 | 0 | 0.611 | 0.929 | 0 | 0.803 | 0.995 |
| *rpsO* | ***rpsG*** | 0.042 | 0 | 0 | 0 | 0.846 | 0.929 | 0 | 0.497 | 0.994 |
| *rpsO* | ***rplP*** | 0 | 0 | 0.252 | 0 | 0.812 | 0.929 | 0 | 0.497 | 0.994 |
| *rpsP* | ***rpsG*** | 0.061 | 0 | 0.167 | 0 | 0.834 | 0.929 | 0 | 0.497 | 0.994 |
| *rpsP* | ***rpsB*** | 0.101 | 0 | 0.208 | 0 | 0.729 | 0.929 | 0 | 0.497 | 0.991 |
| *rpsP* | ***rpsO*** | 0.118 | 0 | 0.189 | 0 | 0.664 | 0.929 | 0 | 0.497 | 0.989 |
| *rpsP* | ***rplP*** | 0.044 | 0 | 0.236 | 0 | 0.655 | 0.929 | 0 | 0.481 | 0.989 |
| *qcrB* | ***ctaD*** | 0.154 | 0 | 0.411 | 0 | 0.846 | 0.322 | 0 | 0.728 | 0.983 |
| *rpsT* | ***rpsP*** | 0.098 | 0 | 0 | 0 | 0.522 | 0.929 | 0 | 0.497 | 0.982 |
| *htaA* | ***hmuT*** | 0.649 | 0 | 0.742 | 0 | 0 | 0 | 0 | 0.759 | 0.976 |
| *rpsT* | ***rpsB*** | 0.099 | 0 | 0 | 0 | 0.202 | 0.929 | 0 | 0.554 | 0.974 |
| *ctaE* | ***qcrA*** | 0.752 | 0 | 0.24 | 0 | 0.093 | 0.326 | 0 | 0.805 | 0.973 |
| *ctaC* | ***qcrA*** | 0.255 | 0 | 0.576 | 0 | 0.28 | 0.326 | 0 | 0.827 | 0.968 |
| *rpsT* | ***rplP*** | 0.049 | 0 | 0 | 0 | 0.12 | 0.929 | 0 | 0.497 | 0.966 |
| *htaA* | ***htaC*** | 0.376 | 0 | 0.783 | 0 | 0 | 0 | 0 | 0.757 | 0.964 |
| *rpsT* | ***rpsG*** | 0 | 0 | 0 | 0 | 0.089 | 0.929 | 0 | 0.497 | 0.964 |
| *htaA* | ***hmuV*** | 0.649 | 0 | 0.621 | 0 | 0 | 0 | 0 | 0.756 | 0.964 |
| *ctaC* | ***ctaF*** | 0.866 | 0 | 0.495 | 0 | 0.064 | 0 | 0 | 0.482 | 0.963 |
| *hmuT* | ***htaC*** | 0.541 | 0 | 0.662 | 0 | 0 | 0 | 0 | 0.757 | 0.959 |
| *ctaE* | ***qcrC*** | 0.752 | 0 | 0.313 | 0 | 0.079 | 0.372 | 0 | 0.554 | 0.948 |
| *hmuV* | ***htaC*** | 0.543 | 0 | 0.564 | 0 | 0 | 0 | 0 | 0.752 | 0.946 |
| *qcrA* | ***ctaD*** | 0.15 | 0 | 0.187 | 0 | 0.077 | 0.314 | 0 | 0.848 | 0.921 |
| *htaA* | ***hmuU*** | 0.558 | 0 | 0.22 | 0 | 0 | 0 | 0 | 0.757 | 0.909 |
| *qcrC* | ***ctaD*** | 0.231 | 0 | 0.2 | 0 | 0.066 | 0.351 | 0 | 0.762 | 0.895 |
| *CP_8515* | ***Cp_8520*** | 0.829 | 0 | 0.253 | 0 | 0 | 0 | 0 | 0 | 0.867 |
| *ctaC* | ***qcrC*** | 0.257 | 0 | 0.475 | 0 | 0.064 | 0.304 | 0 | 0.554 | 0.866 |
| *hmuU* | ***htaC*** | 0.526 | 0 | 0.191 | 0 | 0 | 0 | 0 | 0.656 | 0.856 |
| *CP_3075* | ***CP_3070*** | 0.821 | 0 | 0 | 0 | 0 | 0 | 0 | 0.201 | 0.851 |
| *ctaF* | ***ctaE*** | 0.088 | 0 | 0.252 | 0 | 0.066 | 0 | 0 | 0.778 | 0.839 |
| *ctaF* | ***qcrA*** | 0.093 | 0 | 0.683 | 0 | 0.064 | 0 | 0 | 0.471 | 0.838 |
| *Cp_3070* | ***htaA*** | 0.097 | 0 | 0.767 | 0 | 0 | 0 | 0 | 0.137 | 0.802 |
| *htaG* | ***htaA*** | 0.079 | 0 | 0.78 | 0 | 0 | 0 | 0 | 0 | 0.788 |
| *ctaF* | ***qcrC*** | 0.094 | 0 | 0.732 | 0 | 0.066 | 0 | 0 | 0.104 | 0.769 |
| *AIG11736.1* | ***czcD*** | 0.764 | 0 | 0 | 0 | 0 | 0 | 0 | 0 | 0.764 |
| *ripA* | ***rpfB*** | 0 | 0 | 0 | 0 | 0 | 0 | 0 | 0.757 | 0.757 |
| *ctaF* | ***qcrB*** | 0.061 | 0 | 0.662 | 0 | 0.066 | 0 | 0 | 0.232 | 0.741 |
| *CP_6180* | ***Cp_1235*** | 0 | 0 | 0.736 | 0 | 0 | 0 | 0 | 0 | 0.736 |
| *htaG* | ***Cp_10210*** | 0 | 0 | 0.735 | 0 | 0 | 0 | 0 | 0 | 0.735 |
| *htaF* | ***htaG*** | 0.477 | 0 | 0.496 | 0 | 0 | 0 | 0 | 0.045 | 0.727 |
| *CP_10210* | ***htaC*** | 0 | 0 | 0.72 | 0 | 0 | 0 | 0 | 0 | 0.72 |
| *qcrB* | ***ctaB*** | 0.097 | 0 | 0.453 | 0 | 0.064 | 0 | 0 | 0.43 | 0.701 |
| *qcrC* | ***ctaB*** | 0.231 | 0 | 0.262 | 0 | 0.066 | 0 | 0 | 0.497 | 0.697 |
| *htaF* | ***hmuT*** | 0.098 | 0 | 0.609 | 0 | 0.064 | 0 | 0 | 0.161 | 0.685 |
| *Cp_10210* | ***htaA*** | 0 | 0 | 0.68 | 0 | 0 | 0 | 0 | 0 | 0.68 |
| *htaG* | ***hmuT*** | 0.092 | 0 | 0.662 | 0 | 0 | 0 | 0 | 0 | 0.679 |
| *htaF* | ***Cp_3070*** | 0 | 0 | 0.665 | 0 | 0 | 0 | 0 | 0 | 0.665 |
| *Cp_3070* | ***htaC*** | 0.057 | 0 | 0.656 | 0 | 0 | 0 | 0 | 0.045 | 0.663 |
| *ctaC* | ***rpfB*** | 0 | 0 | 0 | 0 | 0 | 0 | 0 | 0.651 | 0.651 |
| *rbfA* | ***rpsO*** | 0.277 | 0 | 0 | 0 | 0.116 | 0 | 0 | 0.496 | 0.649 |
| *htaF* | ***htaC*** | 0.057 | 0 | 0.638 | 0 | 0 | 0 | 0 | 0.045 | 0.645 |
| *Cp_3070* | ***Cp_10210*** | 0 | 0 | 0.643 | 0 | 0 | 0 | 0 | 0 | 0.643 |
| *Cp_6180* | ***Cp_8515*** | 0 | 0 | 0.592 | 0 | 0 | 0 | 0 | 0 | 0.592 |
| *ctaF* | ***ctaD*** | 0.277 | 0 | 0.172 | 0 | 0.064 | 0 | 0 | 0.357 | 0.591 |
| *htaG* | ***hmuV*** | 0.095 | 0 | 0.562 | 0 | 0 | 0 | 0 | 0 | 0.586 |
| *qcrA* | ***ctaB*** | 0.062 | 0 | 0.29 | 0 | 0.118 | 0 | 0 | 0.369 | 0.58 |
| *htaG* | ***Cp_3070*** | 0.057 | 0 | 0.542 | 0 | 0 | 0 | 0 | 0.045 | 0.551 |
| *Cp_9380* | ***ripA*** | 0.531 | 0 | 0 | 0 | 0 | 0 | 0 | 0 | 0.532 |
| *Cp_3070* | ***hmuT*** | 0.098 | 0 | 0.418 | 0 | 0.064 | 0 | 0 | 0.161 | 0.532 |
| *Cp_6180* | ***pafB*** | 0 | 0 | 0.528 | 0 | 0 | 0 | 0 | 0 | 0.528 |
| *htaF* | ***hmuV*** | 0.098 | 0 | 0.422 | 0 | 0 | 0 | 0 | 0.16 | 0.523 |
| *qcrB* | ***ccsA*** | 0 | 0 | 0.429 | 0 | 0.066 | 0 | 0 | 0.155 | 0.51 |
| *pafB* | ***Cp_8520*** | 0.334 | 0 | 0.285 | 0 | 0 | 0 | 0 | 0 | 0.503 |
| *Cp_8515* | ***czcD*** | 0.502 | 0 | 0 | 0 | 0 | 0 | 0 | 0 | 0.503 |
| *htaF* | ***Cp_10210*** | 0 | 0 | 0.466 | 0 | 0 | 0 | 0 | 0 | 0.466 |
| *qcrA* | ***ndh*** | 0 | 0 | 0 | 0 | 0.356 | 0 | 0 | 0.205 | 0.466 |
| *ctaF* | ***CP_1235*** | 0 | 0 | 0.457 | 0 | 0 | 0 | 0 | 0 | 0.457 |
| *pafB* | ***Cp_8515*** | 0 | 0 | 0.457 | 0 | 0 | 0 | 0 | 0 | 0.457 |
| *Cp_8515* | ***Cp_10210*** | 0 | 0 | 0.454 | 0 | 0 | 0 | 0 | 0 | 0.454 |
| *Cp_3070* | ***hmuV*** | 0.098 | 0 | 0.328 | 0 | 0 | 0 | 0 | 0.16 | 0.446 |
| *ctaF* | ***pafB*** | 0 | 0 | 0.429 | 0 | 0.064 | 0 | 0 | 0 | 0.442 |
| *htaF* | ***Cp_8515*** | 0 | 0 | 0.431 | 0 | 0 | 0 | 0 | 0 | 0.431 |
| *Cp_10210* | ***hmuT*** | 0 | 0 | 0.421 | 0 | 0 | 0 | 0 | 0 | 0.421 |
| *htaF* | ***htaA*** | 0.097 | 0 | 0.77 | 0.58 | 0 | 0 | 0 | 0.137 | 0.411 |
| *Cp_6180* | ***Cp_8520*** | 0 | 0 | 0.409 | 0 | 0 | 0 | 0 | 0 | 0.409 |
| *ctaC* | ***ccsA*** | 0.049 | 0 | 0.281 | 0 | 0.092 | 0 | 0 | 0.148 | 0.4 |

Figure S4. Protein enrichment analyses.


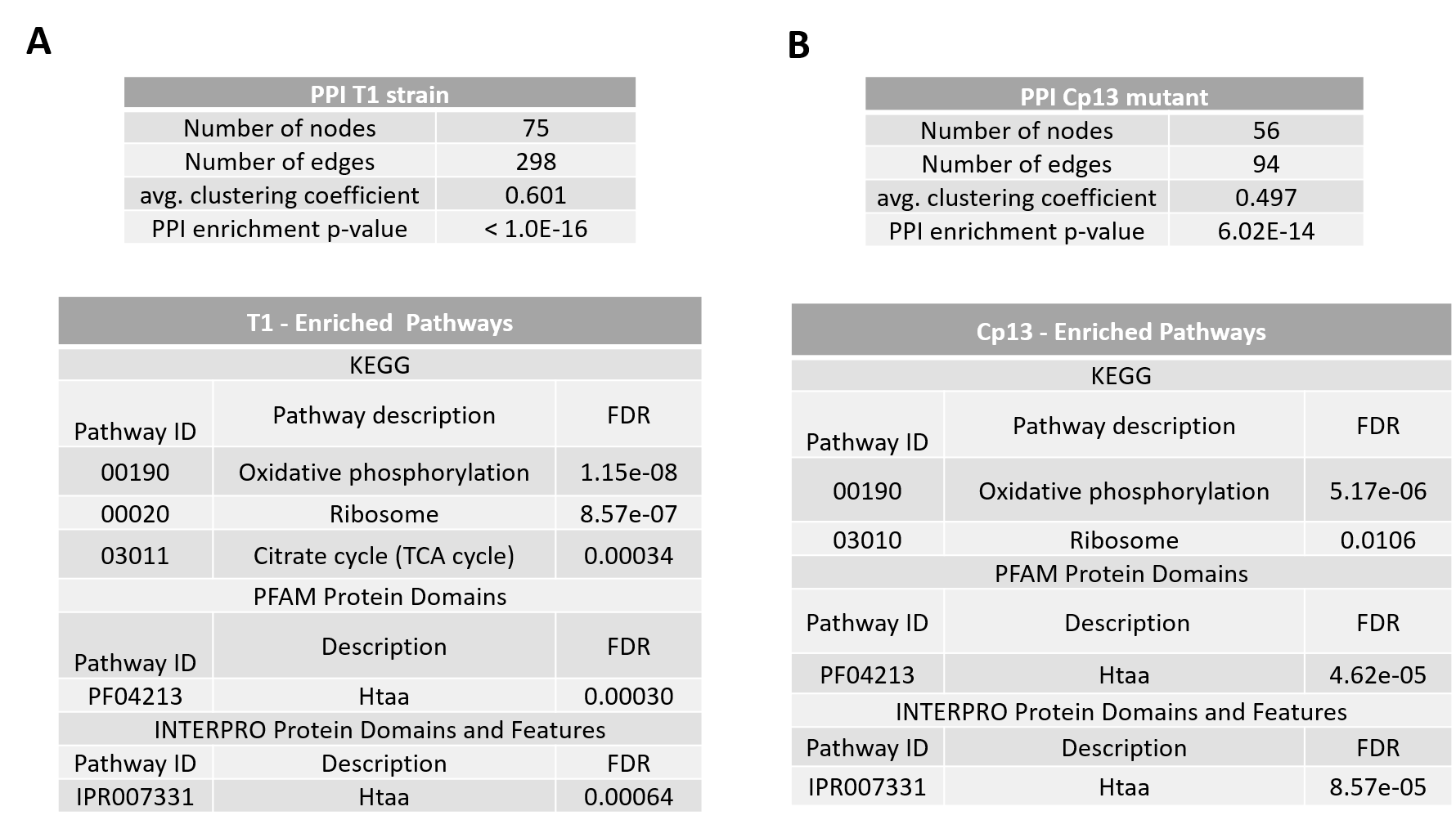

Supplement: Supplementary file 5 — Table S9. Predicted protein-protein interactions of iron regulated genes identified in the wild-type T1 strain. Table S10. Predicted protein-protein interactions of iron regulated genes identified in the Cp13 mutant. Figure S4. Protein enrichment analysis (DOCX 209 kb) [file 12864_2019_6018_MOESM5_ESM.docx]
