# Supplementary material for: Transcriptome profile of Corynebacterium pseudotuberculosis in response to iron limitation
Source: BMC Genomics. 2019 Aug 20;20:663. doi: 10.1186/s12864-019-6018-1 (PMC6701010; doi:10.1186/s12864-019-6018-1)
Supplement: Supplementary file 6 — Figure S5. Alignment of the amino acid sequence of the HtaA domain from protein products of the upregulated genes htaC, htaA, htaF, htaG and Cp_3070 genes of C. pseudotuberculosis. Figure S6. Structural protein domain characteristics of the HtaA, HtaC, HtaF, HtaG and Cp_3070 proteins. Figure S7. Alignment of the amino acid sequences of the HmuU protein between C. pseudotuberculosis (Cp), C. ulcerans (Cu) and C. diphtheriae (Cd). Figure S8. Alignment of the amino acid sequences of the HmuT protein between C. pseudotuberculosis (Cp), C. ulcerans (Cu) and C. diphtheriae (Cd). Figure S9. Alignment of the amino acid sequences of the DNA-binding regulator hrrA of the two-component regulatory system hrrSA between C. pseudotuberculosis (Cp), C. glutamicum (Cg) and C. diphtheriae (Cd). Table S11. Genomic Island predictions (DOCX 4804 kb) [file 12864_2019_6018_MOESM6_ESM.docx]

Additional file 6

**Transcriptome Profile of *Corynebacterium pseudotuberculosis* in Response to Iron Limitation**

Izabela Coimbra Ibraim^1^, Mariana Teixeira Dornelles Parise^1^, Doglas Parise^1^, Michelle Zibetti Tadra Sfeir^2^, Thiago Luiz de Paula Castro^3^, Alice Rebecca Wattan^4^, Preetam Ghosh^5^, Debmalya Barh^1^, Emannuel Maltempi Souza^2^, Aristóteles Góes-Neto^6^, Anne Cybelle Pinto Gomide^a1^, Vasco Azevedo^a1*^

Corresponding Author: Dr. Vasco Azevedo - vasco@icb.ufmg.br

Table of Contents:

Figure S5. Alignment of the amino acid sequence of the HtaA domain from protein products of the upregulated genes *htaC*, *htaA*, *htaF*, *htaG* and *Cp_3070* genes of *C. pseudotuberculosis*

Figure S6. Structural protein domain characteristics of the HtaA, HtaC, HtaF, HtaG and Cp_3070 proteins

Figure S7. Alignment of the amino acid sequences of the HmuU protein between *C. pseudotuberculosis* (Cp), *C. ulcerans* (Cu) and *C. diphtheriae* (Cd)

Figure S8. Alignment of the amino acid sequences of the HmuT protein between *C. pseudotuberculosis* (Cp), *C. ulcerans* (Cu) and *C. diphtheriae* (Cd)

Figure S9. Alignment of the amino acid sequences of the DNA-binding regulator *hrrA* of the two-component regulatory system *hrrSA* between *C. pseudotuberculosis* (Cp), *C. glutamicum* (Cg) and *C. diphtheriae* (Cd)

Table S11. Genomic Island predictions


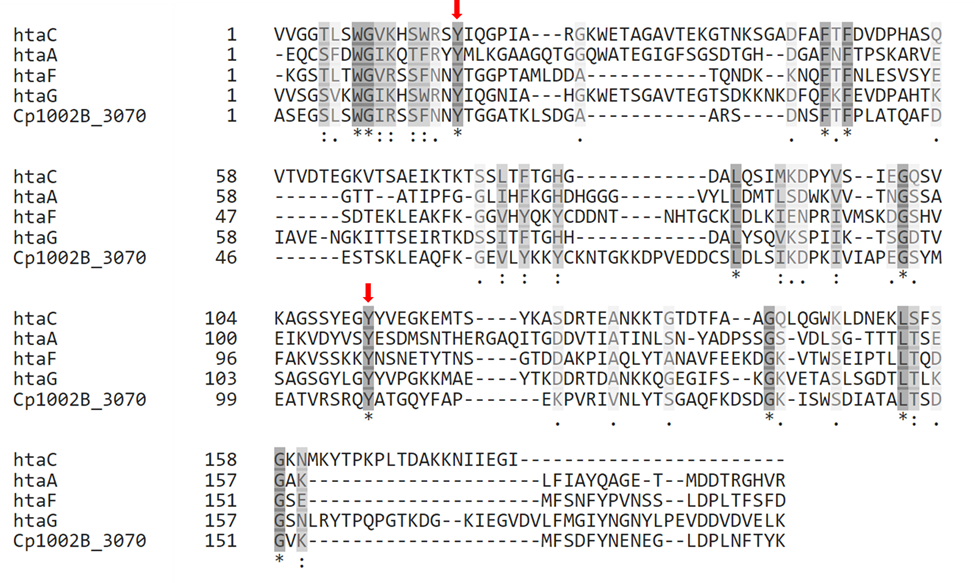


Additional file 6: Figure S4. Alignment of the amino acid sequence of the HtaA domain from protein products of the upregulated genes *htaC*, *htaA*, *htaF*, *htaG* and *Cp1002B_3070* genes of *C. pseudotuberculosis*. Conserved tyrosine residues are indicated above the sequence alignment with arrows; asterisks indicate sequence identity and similarity is indicated by colons and periods.


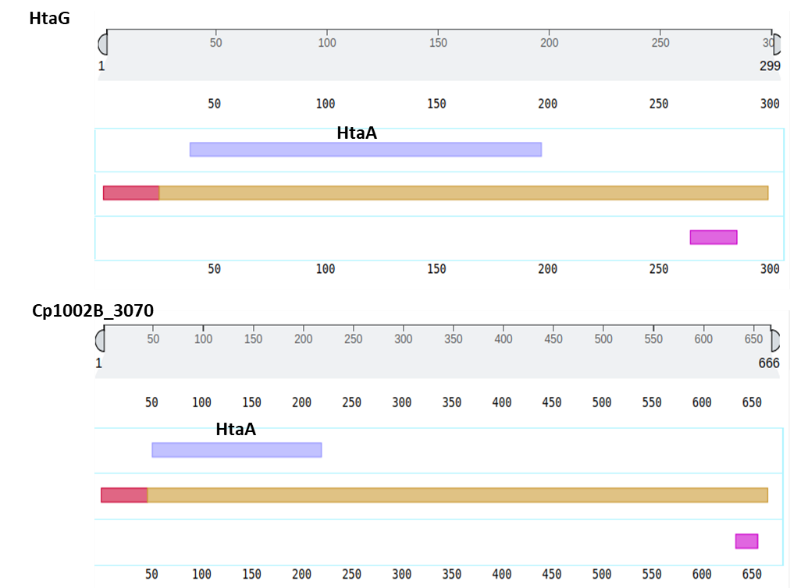

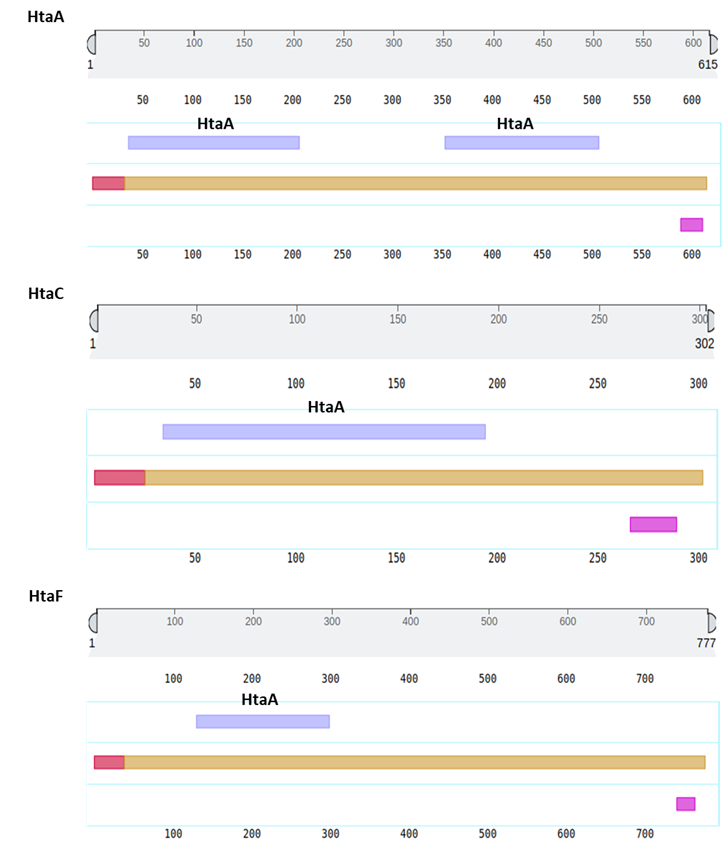


Additional file 6: Figure S5. Structural protein domain characteristics of the HtaA, HtaC, HtaF, HtaG and Cp_3070 proteins. The N-terminal signal peptide, binding HtaA and C-terminal transmembrane domains are schematized by color: signal peptide (red), htaA (purple) and transmembrane (pink). The predicted proteins contain at least one HtaA domain (two for the HtaA protein) consisting of approximately 150 amino acids with a conserved hemin and hemoglobin binding tyrosine residue, a C-terminal transmembrane domain of approximately 22 amino acids responsible for anchoring these proteins to the cytoplasmic membrane with predicted signal peptides.

**
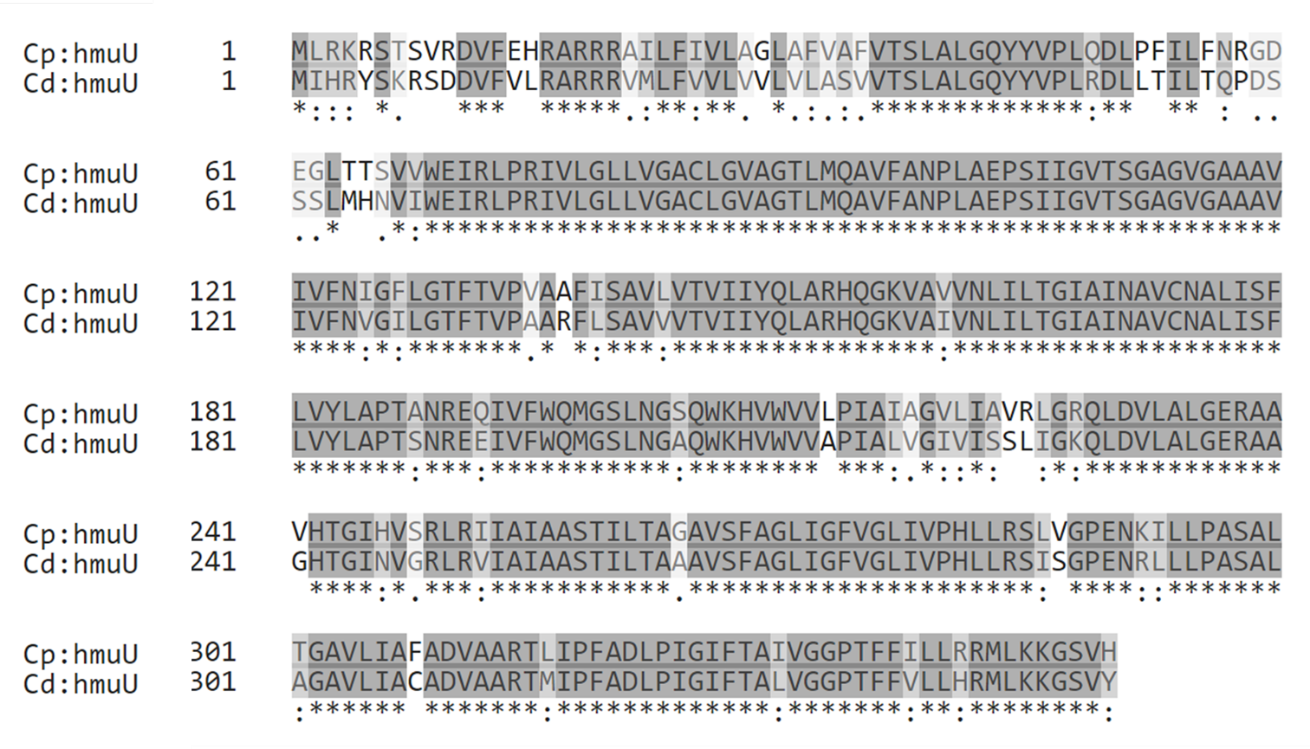
A**

**
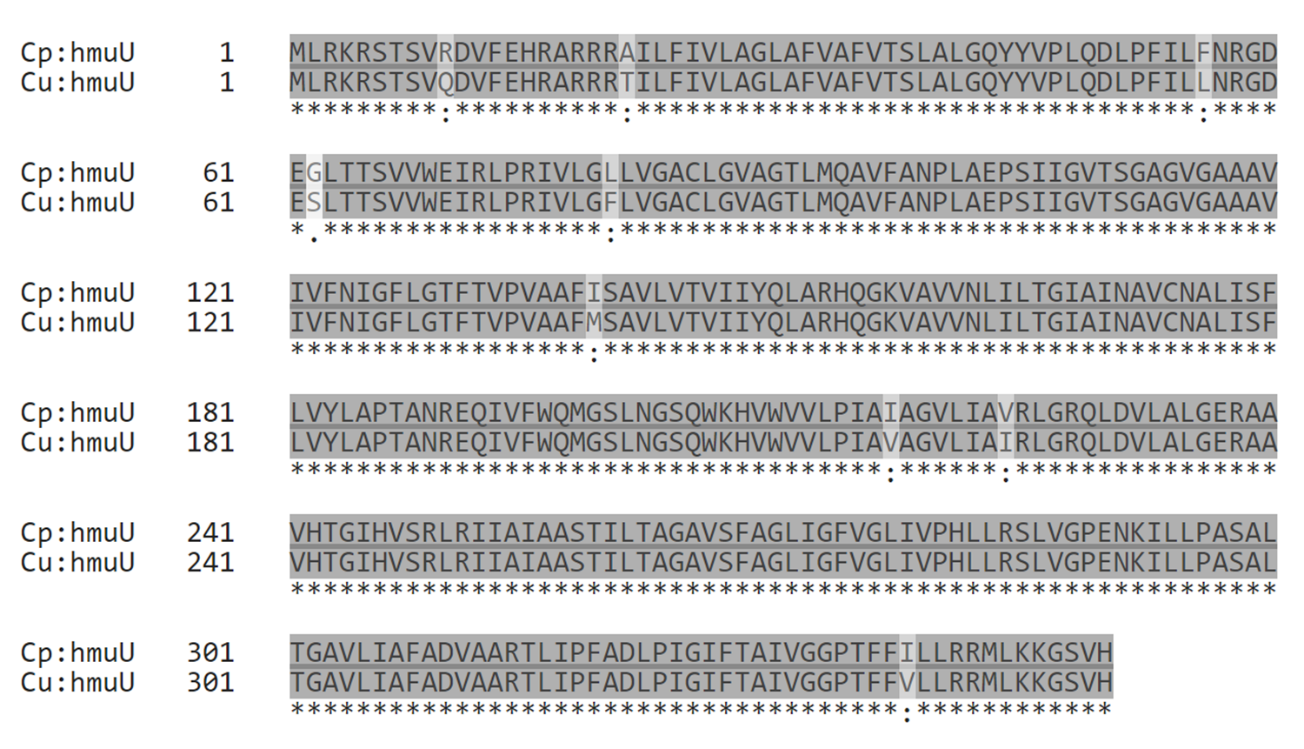
B**

Additional file 6: Figure S6. Alignment of the amino acid sequences of the HmuU protein between *C. pseudotuberculosis* (Cp), *C. ulcerans* (Cu) and *C. diphtheriae* (Cd). Asterisks indicate sequence identity and similarity is indicated by colons and period. (**A**) Cp and Cu alignment with 97.42% amino acid sequence identity; (**B**) Cp and Cd alignment with 80% amino acid sequence identity.


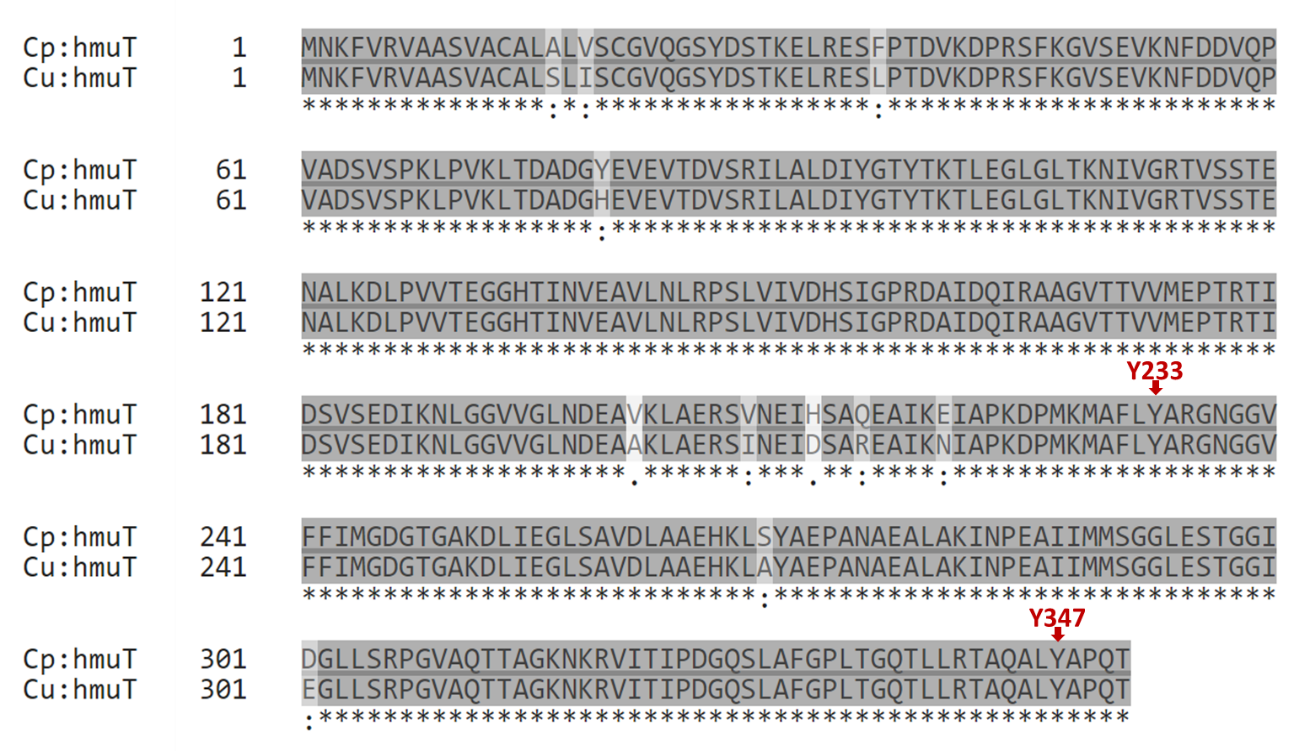
**A**


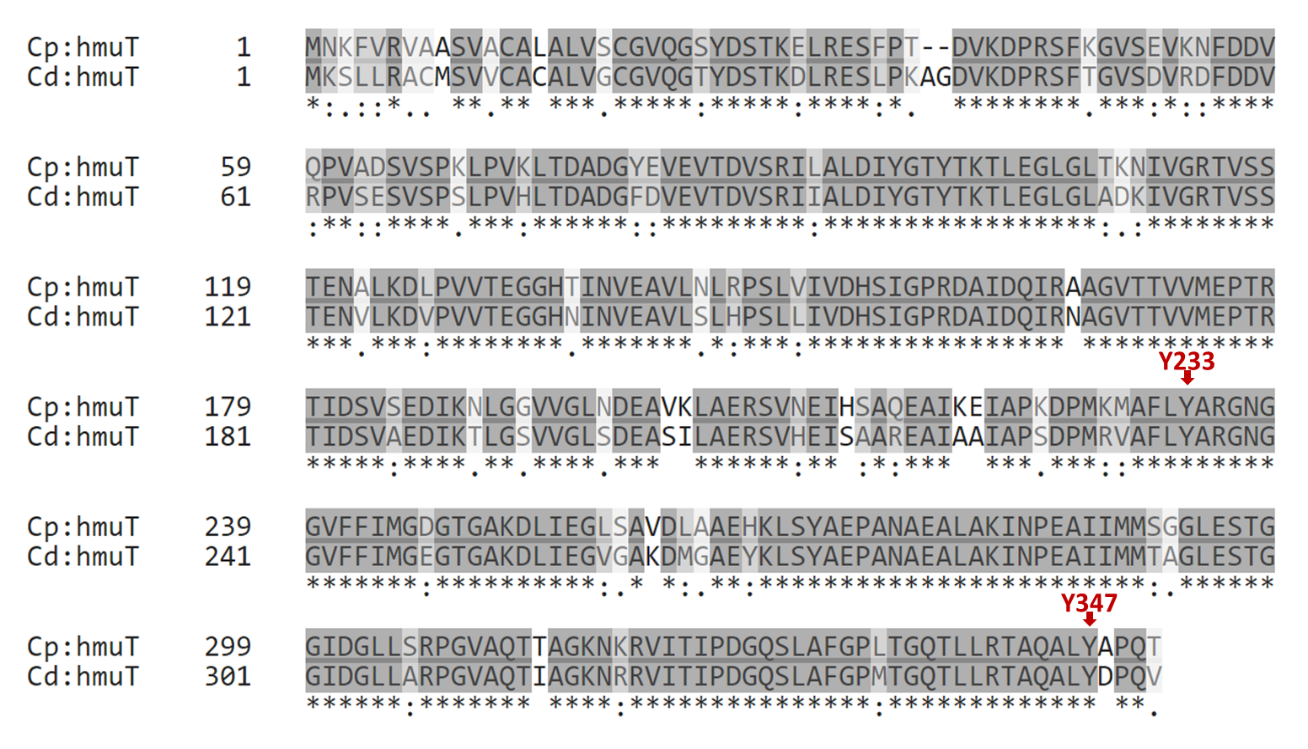
**B**

Additional file 6: Figure S7. Alignment of the amino acid sequences of the HmuT protein between *C. pseudotuberculosis* (Cp), *C. ulcerans* (Cu) and *C. diphtheriae* (Cd). Asterisks indicate sequence identity and similarity is indicated by colons and period. Conserved tyrosine residues (Y233 and Y347) are indicated above the sequence alignment with red arrows (**A**) Cp and Cu alignment with 96.86% amino acid sequence identity; (**B**) Cp and Cd alignment with 80.73% amino acid sequence identity.


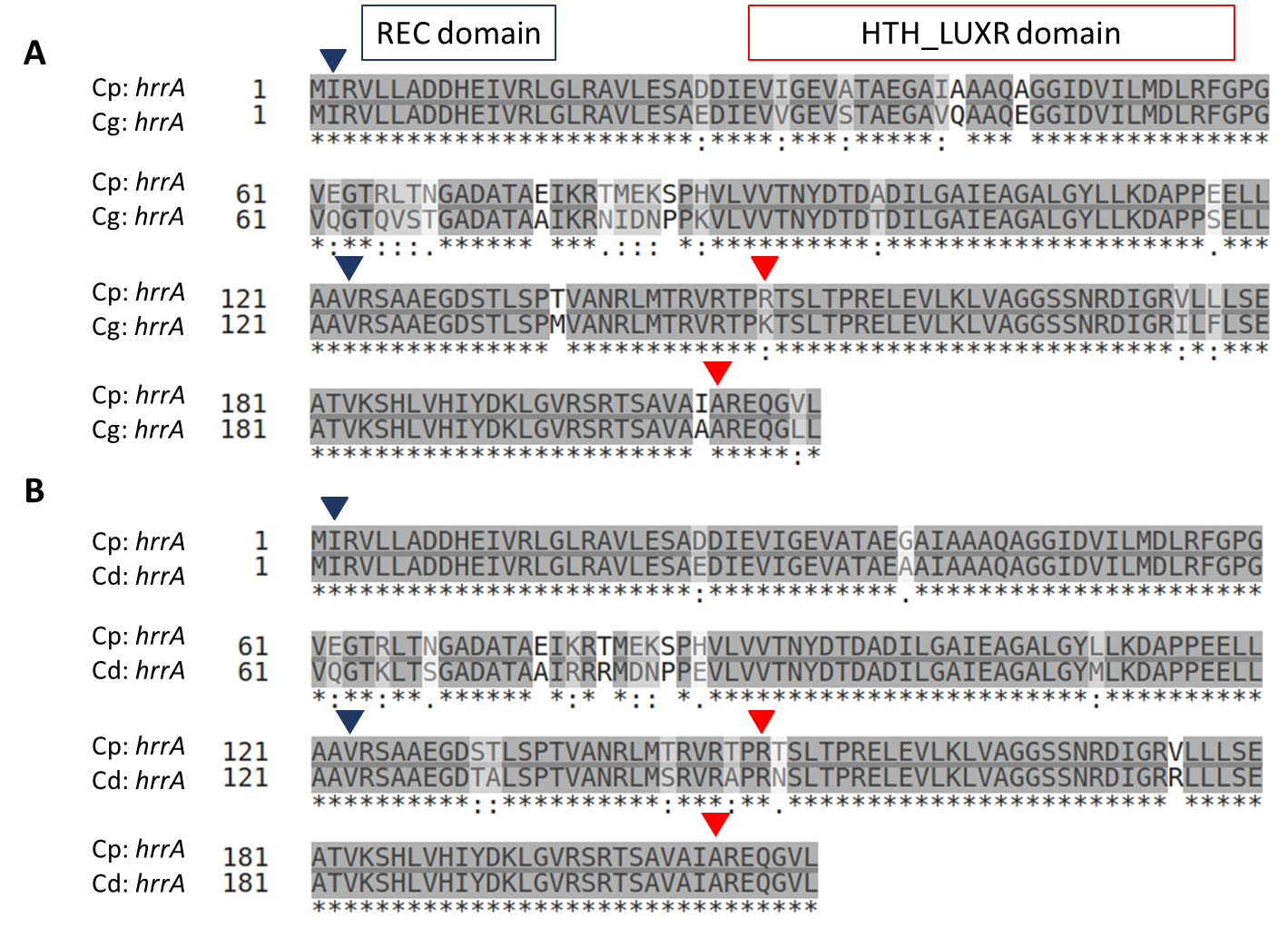


Additional file 6: Figure S8. Alignment of the amino acid sequences of the DNA-binding regulator *hrrA* of the two-component regulatory system *hrrSA* between *C. pseudotuberculosis* (Cp), *C. glutamicum* (Cg) and *C. diphtheriae* (Cd). Asterisks indicate sequence identity and similarity is indicate by colons and period. The two domains of *hrrA* are outlined: REC signal receiver regulatory domain is indicated by blue arrow; red arrow indicates HTH_LUXR domain. (**A**) Cp and Cg alignment with 87.73% amino acid sequence identity; (**B**) Cp and Cd alignment with 91.03% amino acid sequence identity.

Additional file 6: Table S11. Genomic Island (GI) predictions

|  |  |  | Island location | |
| --- | --- | --- | --- | --- |
|  | Genomic islands | Genes located within the islands | start | end |
| T1 strain | Putative Genomic Island 1 | CpT1_RS01715-CpT1_10555 | 359869 | 365559 |
|  | Putative Genomic Island 2 | CpT1_RS02765-CpT1_02850 | 575431 | 604574 |
|  | Putative Genomic Island 3* | CpT1_07140-CpT1_07325 | 1579138 | 1619078 |
|  | Putative Genomic Island 4* | CpT1_08725-CpT1_10755 | 1913406 | 1921880 |
|  | Putative Genomic Island 5* | CpT1_09180-CpT1_09230 | 2013332 | 2022451 |
|  | Putative Genomic Island 6* | CpT1_09890-CpT1_09925 | 2193668 | 2204043 |
|  | Putative Genomic Island 7 | CpT1_10290-CpT1_10355 | 2291646 | 2305455 |
|  |  |  |  |  |
| 1002B strain | Putative Genomic Island 1 | Cp1002B_01700-Cp1002B_10545 | 359878 | 365568 |
|  | Putative Genomic Island 2 | Cp1002B_02750-Cp1002B_02835 | 575437 | 604583 |
|  | Putative Genomic Island 3* | Cp1002B_02990-Cp1002B_03010 | 637219 | 642946 |
|  | Putative Genomic Island 4* | Cp1002B_03040-Cp1002B_03075 | 648004 | 658378 |
|  | Putative Genomic Island 5* | Cp1002B_10625-Cp1002B_04255 | 930172 | 938645 |
|  | Putative Genomic Island 6* | Cp1002B_05640-Cp1002B_05830 | 1232999 | 1272932 |
|  | Putative Genomic Island 7 | Cp1002B_10260-Cp1002B_10325 | 2289552 | 2303361 |

Note: Island prediction was carried out using the T1 and 1002B reference genomes against a closely related nonpathogenic genome (NC_003450). Island location and locus identification of genes within these islands are shown. 7 GIs were predicted in the genome of both strains. *Indicates a putative island harboring differentially expressed genes identified under iron restriction. The genes *Cp_10540* and *Cp_10545* were identified in the genomic island 1; *Cp_10265* island 3; *htaG-htaF* island 4; IlvB1 island 5; *Cp3070-Cp3075* island 6.
